# Methylene-Linked Triazolylidenes as Cooperative Ligands in Manganese-Catalyzed *N*‑Alkylation

**DOI:** 10.1021/acs.inorgchem.6c01357

**Published:** 2026-04-16

**Authors:** Maria Batuecas, Beatriz Garcia, Chiara Saviozzi, Maria S. Viana, Feliu Maseras, Beatriz Royo

**Affiliations:** † Instituto de Tecnologia Química e Biológica António Xavier, ITQB NOVA, 98819 Universidade Nova de Lisboa, Avenida da República, Oeiras 2780-157, Portugal; ‡ Departamento de Química Inorgánica, Instituto de Síntesis Química y Catálisis Homogénea (ISQCH), Facultad de Ciencias, CSIC, 16765 Universidad de Zaragoza, Zaragoza 50009, Spain; § Department of Chemistry and Industrial Chemistry, 9310 University of Pisa, Via G. Moruzzi 13, Pisa I-56124, Italy; ∥ Institute of Chemical Research of Catalonia (ICIQ-CERCA), The Barcelona Institute of Science and Technology, Tarragona 43007, Spain

## Abstract

We elucidate the mechanism of the manganese-catalyzed *N*-alkylation of aniline with benzyl alcohol mediated by a bis­(1,2,3-triazolylidene) Mn­(I) complex through a combination of experimental studies and density functional theory (DFT) calculations. Activation of the precatalyst by a base leads to the formation of an anionic alkoxo complex featuring a deprotonated methylene bridge, which is identified as the catalytically active species. Notably, the methylene linker exhibits previously unrecognized noninnocent behavior, undergoing reversible deprotonation and participating directly in proton-transfer steps of the catalytic cycle. Kinetic isotope effects and deuterium-labeling experiments support the involvement of both hydride transfer and alcohol-assisted proton processes in the rate-determining steps. These findings uncover a new mode of metal–ligand cooperation in triazolylidene-based manganese catalysts and provide mechanistic guidelines for the design of cooperative ligands in base-metal-borrowing hydrogen catalysis.

## Graphical abstract

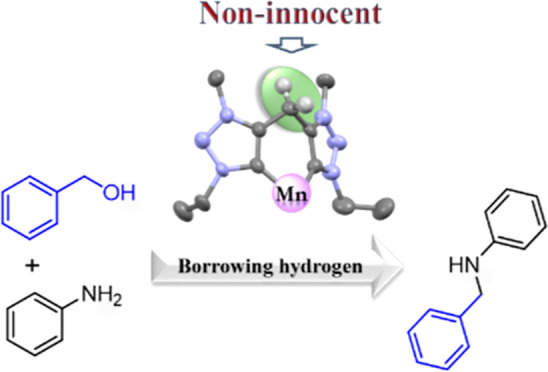

## Introduction

Borrowing hydrogen (BH) has emerged as a powerful and atom economy strategy for C–N bond formation, enabling the direct use of benign and readily available alcohols as alkylating agents.^1–3^ By avoiding preactivated electrophiles and minimizing waste, BH has become an attractive approach for sustainable amine synthesis. While early advances relied predominantly on noble-metal catalysts, recent efforts have focused on developing BH systems based on earth-abundant 3d transition metals.^4–9^ Among these, manganese has gained particular prominence owing to its abundance, low toxicity, and promising catalytic versatility.^10–21^

Manganese complexes supported by N-Heterocyclic carbene (NHC) ligands have recently been shown to mediate BH reactions through nonbifunctional, outer-sphere pathways.^22^ Prior to these developments, our group reported the first Mn­(I) bis-NHC tricarbonyl complexes and demonstrated their high efficiency in a variety of reduction processes.^23–28^ Building on this foundation, we subsequently explored Mn­(I) tricarbonyl complexes supported by bis­(1,2,3-triazolyl-5-ylidene) ligands,^29,30^ a scaffold that has previously been employed in ruthenium-catalyzed BH chemistry.^31^ Despite their structural resemblance to classical NHC ligands, the cooperative potential of triazolylidene systems in Mn-based BH catalysis remains largely unexplored.

In our recent studies, we identified a Mn­(I) tricarbonyl complex supported by a methylene-bridged bis­(1,2,3-triazolyl-5-ylidene) ligand as a highly efficient catalyst for borrowing hydrogen (BH) transformations.^29,30^ This system exhibited remarkable activity in the *N*-alkylation of amines, enabling the synthesis of 1,2,3,4-tetrahydroquinoxalines as well as the selective *N*,*N*′-dialkylation of *o*-phenylenediamines with alcohols. Moreover, the catalyst was capable of promoting both the selective monoalkylation and dialkylation of a broad range of aniline derivatives using aliphatic diols as alkylating agents. Given this broad reactivity profile and high selectivity, we sought to gain deeper insight into the mechanism operating in these transformations and to elucidate the key steps involved in catalysis by this Mn­(I) bis­(triazolylidene) complex.^30^

Herein, we report a combined experimental and computational investigation of the *N*-alkylation of aniline with benzyl alcohol catalyzed by a bis­(1,2,3-triazolylidene) Mn­(I) complex, providing detailed mechanistic insight into the role of ligand participation in the catalytic cycle.

## Results and Discussion

To initiate our study, we investigated the reaction of [Mn­(bis-Trz)­(CO)_3_Br] (**1**) with *t*-BuOK. The reaction was performed in THF at room temperature for 1 h. Under these conditions, complex **1** underwent a clean transformation into the corresponding dimeric Mn­(I) complex **2**, resulting from a single deprotonation of the methylene bridge (Scheme 1).

**Scheme 1:**
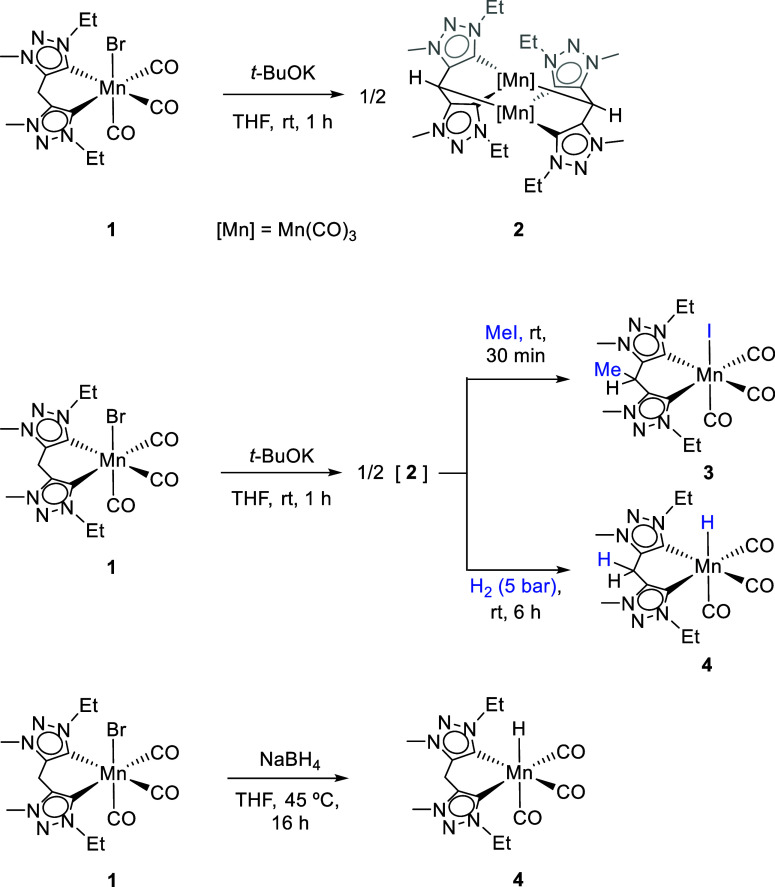
Synthesis of Mn­(I) Complexes **2–4**

Complex **2** was characterized by means of NMR and IR spectroscopy. The ^1^H NMR spectrum in THF-*d*_8_ of **2** shows the resonance corresponding to the CH moiety as a singlet at δ 3.58 ppm. The ^13^C­{^1^H} carbon shift of the metalated carbon appears at δ 14.1 ppm, high-field shifted as a consequence of the metal center shielding. These data are in agreement with previously chemical shifts reported for Mn­(I)–methine complexes.^32,33^ The IR spectrum of **2** shows the typical pattern for *fac*-tricarbonyl complexes, with three intense carbonyl stretches at 1955, 1862, and 1829 cm^–1^. These stretches appear at wavenumbers lower than those for **1**, indicating stronger back-donation from the metal center to the CO ligands. Single crystal X-ray diffraction analysis confirmed the dimeric structure of **2** (Figure 1). The asymmetric unit contains two distinct halves of dimers cocrystallized with THF (Figure S7). Each Mn center is coordinated to the bis-triazolylidene fragment, displaying Mn–C­(triazolylidene) bond lengths between 2.040(8) and 2.063(9) Å, which are consistent with those reported for other Mn-(triazolylidene) complexes.^29^ Additionally, each Mn atom is bonded to the carbon atom of the deprotonated methylene bridge with a Mn–C6 bond length of 2.270(8) Å. This Mn–C6 bond distance is slightly longer than Mn–C­(methine) distances reported for a picolyl–phosphine complex (2.205(2) Å)^32^ and for a related picolyl-NHC dimer, recently reported by Song (2.210(3) Å).^33^

**Figure 1:**
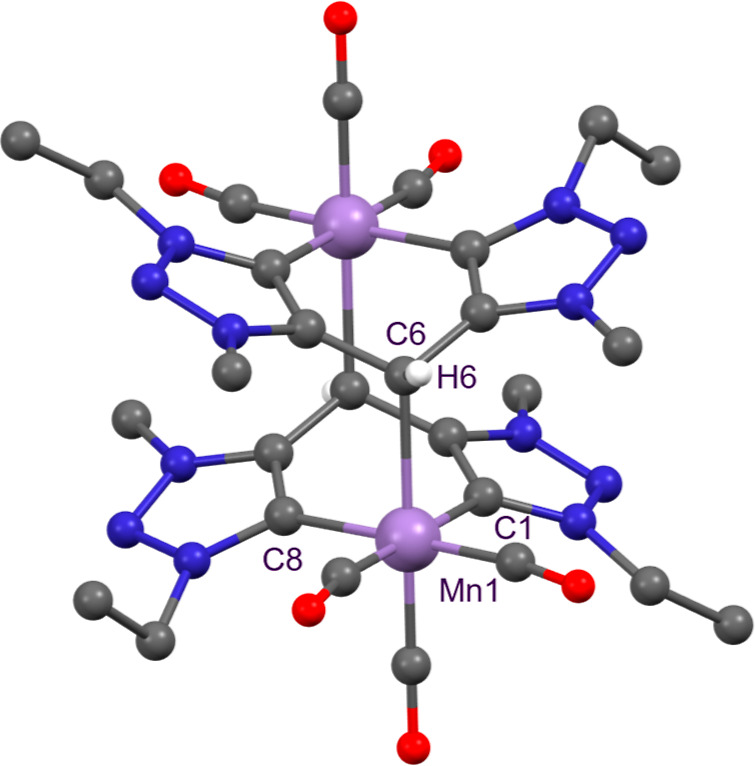
Molecular structure of complex **2**. One THF molecule and H atoms (except H6) have been omitted for clarity.

Notably, we report here the first example of methylene-bridge deprotonation in a bis-triazolylidene ligand coordinated to a manganese center. A related transformation was independently identified in a W­(bis-triazolylidene)­(CO)_4_ system studied in parallel in our laboratory, further underscoring the inherent noninnocent character of the methylene-bridged bis-triazolylidene framework.^34^ Sarkar and co-workers previously reported a related single deprotonation in a methylene-bridged bis-triazolium salt, which, upon redox-induced radical dimerization under formal dihydrogen release, led to the formation of a tetratriazoliumethylene species.^35^ The observation of ligand-centered reactivity across distinct metal platforms highlights the potential for metal–ligand cooperation (MLC) in these systems.

This deprotonation at the methylene bridge paves the way for the modular synthesis of manganese complexes featuring bis-triazolylidene ligands with tailored substituents at the bridging position. As a proof of concept, treatment of complex **1** with *t*-BuOK to generate **2** in situ, followed by the addition of one equivalent of MeI, afforded complex **3** via selective methylation at the carbon atom linking the two triazolylidene fragments (Scheme 1). The CH resonance of the methylated bridge appears as a quartet (^3^J_HH_ = 6.3 Hz) at δ 4.75 ppm in the ^1^H NMR spectrum, while in the ^13^C­{^1^H} NMR spectrum, the corresponding carbon appears at δ 28.5 ppm, resembling chemical shifts to those reported for complex **1**.^29^

To evaluate whether deprotonation of complex **1** is reversible, it was initially treated with *t*-BuOK in THF and subsequently exposed to H_2_ (5 bar) for 6 h. This procedure led to the clean formation of the Mn­(I) hydride species [Mn­(bis-Trz)­(CO)_3_H] (**4**) (Scheme 1), as evidence by a characteristic hydride signal^23–25,33,36–38^ at −6.44 ppm observed in the ^1^H NMR spectrum and the bridging –CH_2_– carbon appearing at δ 22.9 ppm in the ^13^C­{^1^H} NMR spectrum. This reactivity implies heterolytic cleavage of H_2_ by dimer species **2** promoted by both the ligand and metal center. Alternatively, complex **4** could be synthesized by treating **1** with an excess of NaBH_4_ in THF at room temperature (Scheme 1). Under these conditions, **4** was isolated as a crystalline red solid in high yield, and its structure was unambiguously confirmed by single crystal X-ray diffraction analysis (Figure 2). The molecular structure reveals a Mn–H bond length of 1.48(5) Å, which is shorter than those reported for related [Mn­(bis-NHC)­(CO)_3_H] (1.79(3) Å)^15,36^ and picolyl–NHC Mn hydride complex (1.59(3) Å).^33^ The dihedral angle between the triazolylidene rings increased from 33.50(18)° in complex **1** ^29^ to 40.6(4)° and 43.4(4)° in complex **2** and 53.3(3)° in complex **4**. This increase may be attributed to the reduced steric hindrance associated with hydride in the axial position.

**Figure 2:**
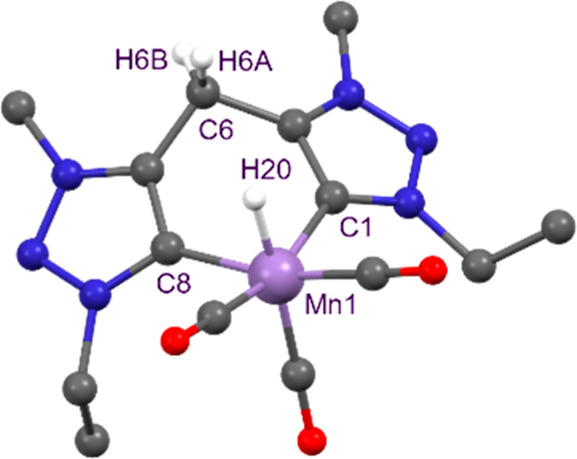
Molecular structure of complex **4**. One THF molecule and H atoms (except H20, H6A, and H6B) have been omitted for clarity.

The catalytic activity of Mn complexes **2** and **4** was evaluated in the *N*-alkylation of *p*-toluidine with benzyl alcohol under the reaction conditions previously established by our group in order to compare their performance with **1** (1.5 mol % of [Mn] and 50 mol % of *t*-BuOK, neat, at 100 °C for 2 h).^29^ Under these conditions, complexes **1**, **2**, and **4** all displayed comparable reactivity, each affording *N*-alkylated amine in quantitative yield (Table S2). In contrast, when the same reactions were conducted in the absence of base, no product formation was observed in any case (Table S3). In order to elucidate the role of the base in the reaction, we tested whether dimer **2** can operate in the presence of catalytic amounts of base. Complex **2** was generated in situ and its catalytic activity was evaluated in the model reaction between aniline and benzyl alcohol using 4.5 mol % of *t*-BuOK, 1.5 mol % of **2** in THF at 100 °C. Under these conditions, the *N*-alkylated amine was obtained in 58% yield after 2 h (reaching quantitative yield in 20 h, Figure S1). These results indicate that the primary role of the base is to activate **1**, leading to formation of the catalytically active species, and importantly also show that the amount of base can be reduced to catalytic amounts which is highly desirable to improve the sustainability and practicality of the process.

To probe the operational robustness of the catalytic system, consecutive substrate additions were performed using the same reaction mixture without a further catalyst and base addition. Fresh portions of aniline and benzyl alcohol were introduced every 2 h under the standard reaction conditions. In the first four runs, a complete conversion was consistently reached within 2 h. The reaction mixture was then stirred for 20 h, after which a further portion of aniline and benzyl alcohol was added. After an additional 2 h, the *N*-alkylated amine was obtained in 84% yield, showing that the active species retains significant catalytic activity after prolonged operation, albeit with partial loss of performance (Figure S2).

Based on the experimental results and to gain insight into the mechanism of the Mn-catalyzed *N*-alkylation of amines with alcohols, as well as to evaluate the potential noninnocent behavior of the bis-triazolylidene ligand, detailed DFT calculations were undertaken.

We first examined the formation of the catalytic species. Once complex **2** is formed by reaction of **1** with *t*-BuOK under catalytic conditions, two scenarios are possible: **2** may react with benzylic alcohol to give the alcohol coordinated species **Int1A** or it may react with the corresponding potassium alkoxide (generated in situ by deprotonation of benzyl alcohol with *t*-BuOK) to afford the anionic intermediate **Int1** (Scheme 2a). Thermodynamically, the formation of **Int1** is favored by 4.6 kcal mol^–1^. From dimer **2**, formation of **Int1** is exergonic by 3.2 kcal mol^–1^, while formation of **Int1A** is slightly endergonic, whereas formation of either species is exergonic when starting from precatalyst **1** (Scheme 2a). Notably, the calculated interconversion between **Int1A** and **Int1** is thermodynamically and kinetically accessible. In **Int1A**, the deprotonated methylene bridge can be protonated by the coordinated alcohol via a six-membered transition state (TSA, Δ*G*_373K_^‡^ = +7.2 kcal mol^–1^) to afford the alkoxo intermediate **IntA**, which then undergoes bridge deprotonation by an external base to furnish **Int1** (Scheme 2b).

**Scheme 2:**
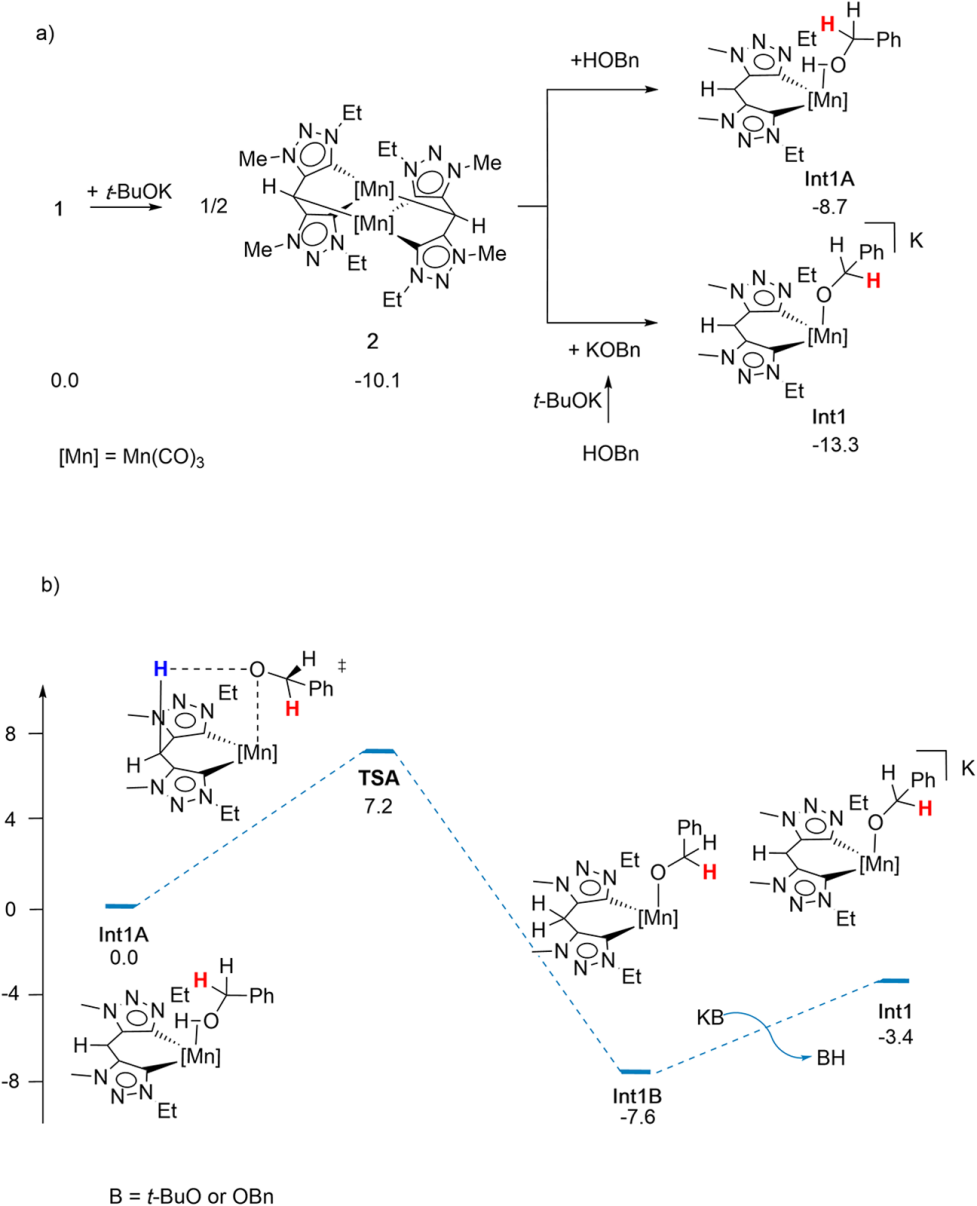
(a) Calculated Gibbs Free Energies for Transformation of **1** into **Int1** and **Int1A**. (b) Proposed Conversion of **Int1A** into **Int1**

In line with these results, the proposed mechanism depicted in Figure 3 begins with **Int1** as the active catalytic species, as it is the most thermodynamically favorable intermediate formed from precatalyst **1**.

**Figure 3:**
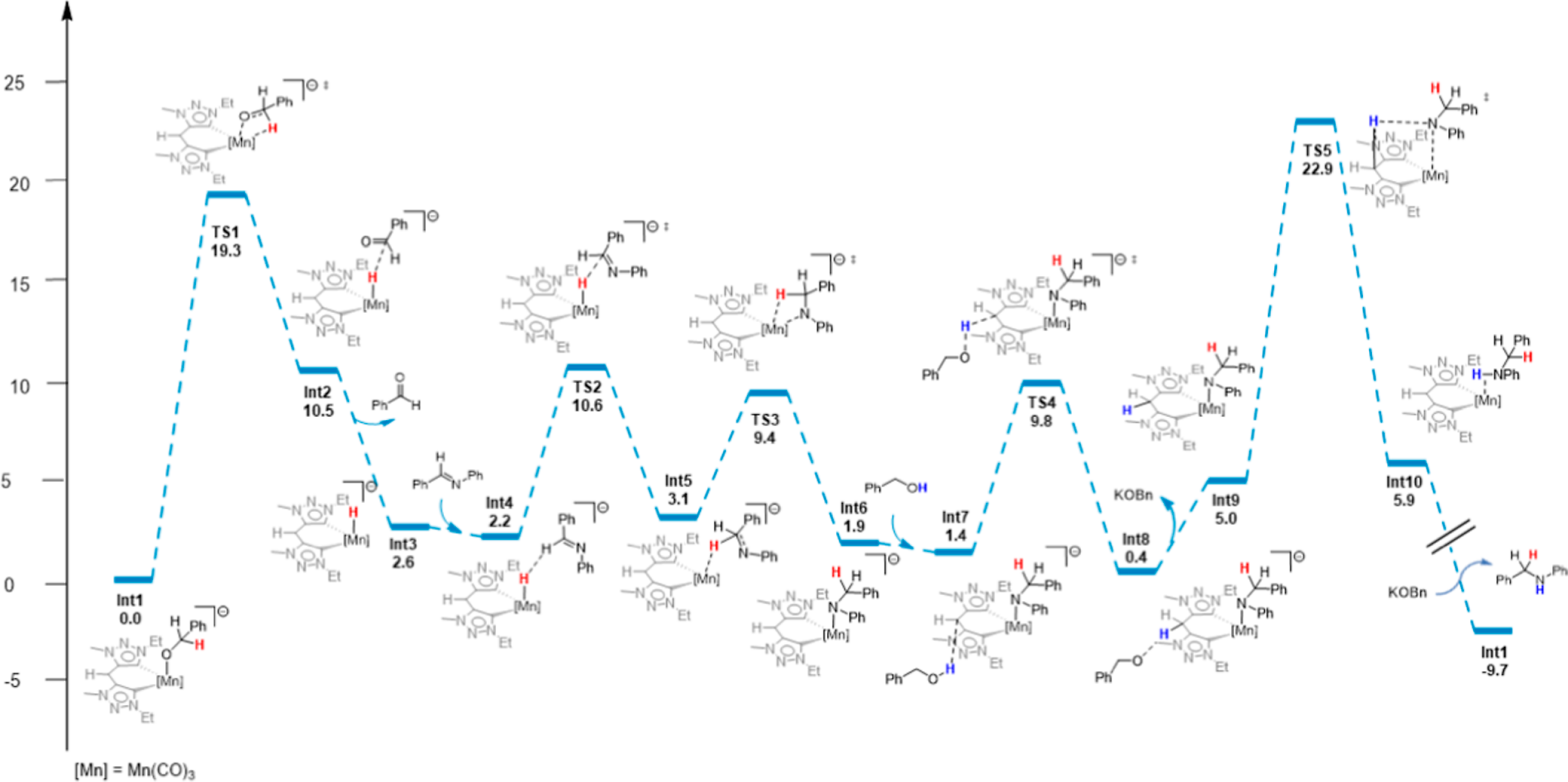
DFT-calculated mechanism for *N*-alkylation of aniline with benzyl alcohol catalyzed by **1**.

As shown in Figure 3, **Int1** is first converted into the encounter intermediate **Int2** via β-hydride elimination, with an associated energy barrier of 19.3 kcal mol^–1^ (TS1). Subsequent aldehyde dissociation affords the hydride species **Int3**, which is the deprotonated analogue of complex **4**.[Fn fn1] The hydride is then transferred from encounter intermediate **Int4** to the C atom of the C=N double bond of the imine through **TS2** (Δ*G*_373K_^‡^ = +10.6 kcal mol^–1^), yielding intermediate **Int5**. This is followed by a change in the ligand coordination mode via **TS3** (Δ*G*_373K_^‡^ = +9.4 kcal mol^–1^), leading to the formation of **Int6**, consistent with a formal hydride insertion step. Subsequently, a molecule of alcohol protonates the methylene bridge carbon of **Int7** via **TS4** (Δ*G*_373K_^‡^ = +9.8 kcal mol^–1^) to generate **Int8**, which, after release of a KOBn molecule, furnishes the neutral species **Int9**. Protonation of the amide nitrogen in **Int9** then forms **Int10** via **TS5** (Δ*G*_373K_^‡^ = +22.9 kcal mol^–1^). Finally, reaction of **Int10** with KOBn releases the *N*-alkylated amine product and regenerates the active catalytic species **Int1**, thus completing the catalytic cycle.

Overall, this catalytic cycle is exergonic by 9.7 kcal mol^–1^, with the highest activation free-energy barriers calculated as +19.3 for β-hydride elimination (**TS1**) and +21.5 kcal mol^–1^ for bridge-assisted proton transfer (**TS5**), in good agreement with the experimentally applied reaction temperature of 100 °C. This mechanistic picture is robust and remains consistent across a range of tested DFT functionals (Table S7).

To gain mechanistic insight into the catalytic cycle, kinetic isotope effect (KIE) studies were performed using isotopically benzyl alcohol derivatives (Figure S3). When benzyl alcohol-OD (PhCH_2_OD) was employed, a KIE value of 2.3 was observed, while the use of benzyl-α-d alcohol (PhCD_2_OH) gave a KIE of 1.9. The significant isotope effect observed for the O–D-substituted alcohol indicates that proton transfer involving the H/D atom of the hydroxyl group is kinetically relevant in the catalytic cycle. At the same time, the measurable isotope effect for the benzylic C–H/D bond suggests that benzylic C–H activation during alcohol dehydrogenation also contributes to the overall rate. The observation of comparable KIE values for both positions indicates that the catalytic turnover does not proceed through a single isolated rate-determining step. Instead, the results point to shared rate control involving both alcohol dehydrogenation (β-hydride elimination, **TS1**) and alcohol-assisted proton-transfer processes (**TS5**). These findings are fully consistent with the DFT-derived metal–ligand cooperative borrowing hydrogen mechanism in which both hydride transfer and proton shuttling steps present the highest energy barriers of the proposed mechanism, 19.3 and 22.9 kcal mol^–1^, respectively.

Because **Int1** can interconvert with its bridge-protonated form **Int1B** under catalytic conditions (Scheme 2b), we also evaluated a mechanism initiated by **Int1B** (Figure S14). This pathway features a β-hydride elimination barrier 9.6 kcal mol^–1^ higher than that of the main mechanism, making it kinetically unlikely.

In order to provide experimental evidence for the involvement of the ligand bridge in the catalytic reaction and support the ligand-assisted pathway suggested by the DFT studies, we performed a deuterium labeling experiment. Complex **4-D** was generated by reaction of the in situ formed dimer **2** with D_2_, which resulted in full conversion to the deuterated species. The ^2^H NMR spectrum of **4-D** in THF-*h*_8_ shows deuterium incorporation both at the ligand methylene bridge and at the metal–deuteride position, whereas these signals are absent in the ^1^H NMR spectrum recorded in a deuterated solvent.

The in situ generated complex **4-D** was subsequently employed as a precatalyst (20 mol %) in the *N*-alkylation of aniline with benzyl alcohol (Scheme 3, Figure S6). Quantitative ^1^H NMR analysis of the reaction mixture after heating at 100 °C for 2 h showed full conversion to the product and revealed 30% deuterium incorporation at the benzylic methylene group of the *N*-benzylaniline. Since the catalyst loading was 20 mol %, the observed deuterium incorporation exceeding this value indicates that not only the metal deuteride but also the deuterium located at the ligand methylene bridge participates in hydrogen transfer during catalysis.

**Scheme 3:**
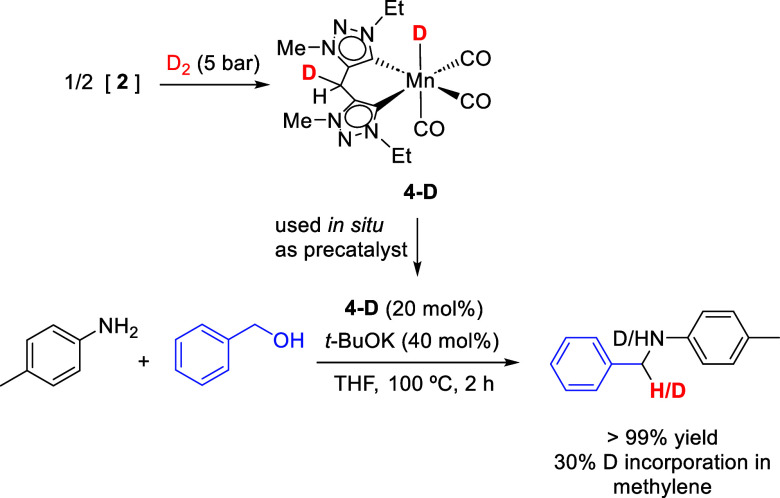
Deuterium-Labeling Experiment Demonstrating Transfer of Deuterium from the Ligand Methylene Bridge of Complex **4-D** during Mn-Catalyzed *N*-Alkylation of Aniline with Benzyl Alcohol

Taking into account the experimental evidence and DFT results, we propose the simplified catalytic cycle depicted in Figure 4. The reaction begins with the activation of the precatalyst **1** by deprotonation of the methylene bridge, followed by reaction with KOBn to generate anionic alkoxo active species **Int1**. A subsequent β-hydride elimination affords the hydride derivative **Int3** together with a molecule of aldehyde, which then condenses with the amine to form the corresponding imine.[Fn fn2] **Int3** undergoes hydride migratory insertion into the C=N bond to give **Int6**. Protonation of the methine (−CH−) fragment of **Int6** by an external alcohol produces **Int8** along with a molecule of KOBn. The amide fragment of **Int8** is then protonated by the methylene bridge of the ligand to afford **Int10**, which reacts with the previously generated KOBn to release the *N*-alkylated amine product and regenerate the active species **Int1**.

**Figure 4:**
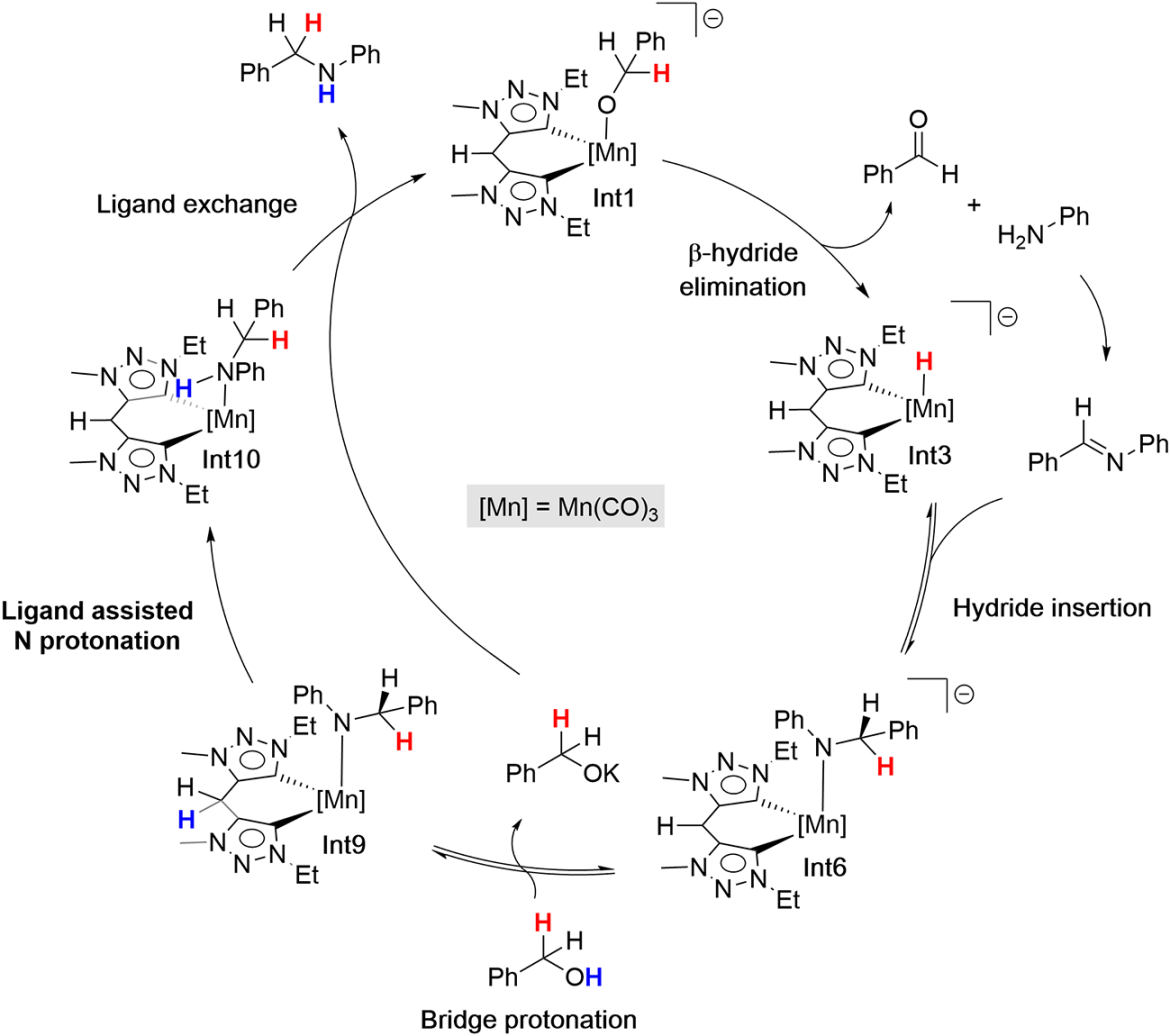
Simplified proposed catalytic cycle for *N*-alkylation of aniline with benzyl alcohol catalyzed by **1**.

## Conclusions

In this work, we show that methylene-bridged bis­(1,2,3-triazolylidene) ligands can engage in previously unrecognized modes of metal–ligand cooperation, broadening the conceptual scope of triazolylidene chemistry. We identify the anionic Mn-alkoxo species bearing a deprotonated methylene bridge as the active catalyst, demonstrating that the methylene linker acts as a noninnocent site capable of reversible proton exchange. This cooperative behavior enables alternative reactivity pathways that are inaccessible to purely metal-centered mechanisms, providing a new strategy to modulate key steps in BH transformations and highlighting the broader potential of ligand-assisted processes in base-metal catalysis.

## Experimental Section

### General Procedures and Materials

All reactions and manipulations for the syntheses of ligands and metal complexes and catalytic experiments were performed under the exclusion of air and moisture using standard Schlenk techniques and a glovebox. Solvents were purified using appropriate drying agents and stored under molecular sieves under a nitrogen atmosphere. Deuterated solvents were degassed and stored in molecular sieves. All other reagents were purchased from commercial suppliers and used without further purification. Infrared spectra were recorded on samples as KBr pellets using a Bruker IFS 66/S instrument or as solids using an ATR-FTIR spectrometer. ^1^H and ^13^C NMR spectra were recorded on a Bruker Avance III 300 MHz, Bruker Avance III 400 MHz, and Bruker Avance III 800 MHz spectrometers. Chemical shifts are expressed as δ (parts per million) relative to residual solvent signals, and *J* values are given in Hertz. Data were processed using MestReNova software. When needed, chemical shifts were assigned with the assistance of 2D NMR (HSQC, HMBC, and COSY) spectra. Manganese complex **1** was prepared following the procedure previously reported by us.^29^

### Synthesis and Characterization of **2**

In a Schlenk flask, [Mn­(bis-Trz)­(CO)_3_Br] (**1**) (100 mg, 0.22 mmol) and *t*-BuOK (72 mg, 0.66 mmol) were combined and subjected to vacuum for 10 min. Subsequently, dry THF (5 mL) was added, and the resulting mixture was stirred at room temperature for 1 h. The dark-red solution was then filtered through a pad of Celite, and the filtrate was concentrated to approximately 0.3 mL. Addition of hexane induced the precipitation of a brown solid, which was collected and washed with hexane (2 × 3 mL), affording complex **2** as a brown solid in 66% yield (54 mg, 0.072 mmol). Single crystals suitable for X-ray diffraction analysis were obtained by layering hexane over a concentrated THF solution of **2** and storing the mixture at −20 °C for 1 week. ^1^H NMR (400 MHz, 298 K, THF-*d*_8_): δ 4.44 (m, 8H, 4 × C*H*_2_CH_3_), 4.02 (s, 12H, 4 × NC*H*_3_), 3.58 (s, 2H C*H*, overlapping signal with THF-*d*_8_), 1.40 (t, ^3^ *J*_*HH*_ = 7.7 Hz, 12H, 4 × CH_2_C*H*_3_). ^13^C­{^1^H} NMR (201 MHz, 298 K, THF-*d*_8_): δ 225.9 (4 × *C*O), 224.7 (2 × *C*O), 170.9 (2 × *C*-Mn), 158.7 (4 × C_trz_-NCH_3_), 48.3 (4 × *C*H_2_CH_3_), 35.4 (4 × N*C*H_3_), 16.7 (4 × CH_2_ *C*H_3_), 14.1 (2 × *C*H). Selected IR data (KBr): ν (CO) 1955 s, 1862 s, 1829 s cm^–1^. Note: Because of its extreme sensitivity, elemental analysis of complex **2** could not be performed.

### In Situ Generation of Mn Complex **3**

[Mn­(bis-Trz)­(CO)_3_Br] (**1**) (10 mg, 0.022 mmol) and *t*-BuOK (7.2 mg, 0.066 mmol) were placed in an NMR tube inside a glovebox. The NMR tube was sealed and removed from the glovebox after which THF-*d*_8_ (0.4 mL) was added. The reaction mixture was allowed to stand at room temperature for 1.5 h. A ^1^H NMR spectrum was then recorded, confirming the formation of **2**. Subsequently, MeI (2.8 μL, 0.044 mmol) was added via a syringe, and the reaction was kept at room temperature for 30 min. A ^1^H NMR spectrum recorded after this time confirmed the complete formation of **3**. Compound **3** was characterized by NMR and IR spectroscopy. ^1^H NMR (300 MHz, 298 K, THF-*d*_8_): δ 5.01 and 4.88 (both m, 2H each, 2 × C*H*_2_CH_3_), 4.75 (q, ^3^ *J*_*HH*_ = 6.3 Hz, 1H, CH_3_C*H*), 4.22 (s, 6H, 2 × NC*H*_3_), 1.64 (overlapping signals, 3H, C*H*_3_CH), 1.62 (overlapping signals, 6H, 2 × CH_2_C*H*_3_). ^13^C­{^1^H} NMR (75 MHz, 298 K, THF-*d*_8_): δ 176.3 (*C*–Mn), 145.9­(2 × C_trz_-NCH_3_), 50.5 (2 × *C*H_2_CH_3_), 36.5 (2 × N*C*H_3_), 28.5 (CH_3_ *C*H), 20.0 (*C*H_3_CH), 16.1 (2 × CH_2_ *C*H_3_). Selected IR data (ATR): ν (CO) 1984 and 1871 s cm^–1^.

### Synthesis and Characterization of **4**

[Mn­(bis-Trz)­(CO)_3_Br] (**1**) (50 mg, 0.11 mmol) and NaBH_4_ (42 mg, 1.11 mmol) were added to a Schlenk flask and placed under vacuum for 10 min. Dry THF (4 mL) was then added, and the mixture was stirred at 45 °C for 12 h. The resulting red solution was filtered through a pad of Celite, and the filtrate was concentrated to approximately 0.3 mL: Hexane was then added to induce the precipitation of a dark-red solid. The solid was collected by filtration and washed with hexane (2 × 3 mL), affording **4** in 61% yield (25 mg, 0.067 mmol). Crystals suitable for X-ray diffraction analysis were obtained by layering hexane over a THF concentrated solution of **4** and storing the mixture at −20 °C for 1 week. ^1^H NMR (400 MHz, 298 K, THF-*d*_8_): δ 4.65 (m, 4H, 2 × C*H*_2_CH_3_), 4.02 (overlapping signals, 6H, 2 × NC*H*_3_ and 2H −C*H*_2_−), 1.53 (t, ^3^ *J*_*HH*_ = 7.2 Hz, 6H, 2 × CH_2_C*H*_3_), −6.44 (s, 1H, Mn*H*). ^13^C­{^1^H} NMR (100 MHz, 298 K, THF-*d*_8_): δ 188.4 (*C*–Mn), 141.8 (2 × C_trz_-NCH_3_), 48.4 (2 × *C*H_2_CH_3_), 35.7 (2 × N*C*H_3_), 22.9 (−*C*H_2_–), 16.7 (2 × CH_2_ *C*H_3_). Selected IR data (KBr): ν (CO) 1998 and 1887 s cm^–1^. HR-MS (ESI^+^, *m*/*z*): calc. for C_14_H_18_MnN_6_O_3_, [M–H]^+^ = 373.0821; found = 373.0778. Note: Because of its extreme sensitivity, elemental analysis of complex **4** could not be performed.

### In Situ Generation of Mn Hydride **4**

[Mn­(bis-Trz)­(CO)_3_Br] (**1**) (10 mg, 0.022 mmol) and *t*-BuOK (7.2 mg, 0.066 mmol) were placed in a J. Young NMR tube inside a glovebox. The tube was sealed and removed from the glovebox, and THF-*d*_8_ (0.4 mL) was added. The reaction mixture was left to stand at room temperature for 1.5 h, after which a ^1^H NMR spectrum was recorded, confirming the formation of deprotonated intermediate **2**. The solution was then frozen by immersing the tube in liquid nitrogen. The headspace was evacuated, and H_2_ gas (∼5 bar) was introduced. The reaction was monitored by ^1^H NMR spectroscopy until full conversion to the Mn hydride **4** was observed after 6 h.

### General Procedure for the Catalytic *N*-Alkylation of *p*-Toluidine with Benzyl Alcohol

The corresponding manganese complex (1.5 mol %), *p*-toluidine (0.5 mmol), *t*-BuOK (0.25 mmol), and benzyl alcohol (0.75 mmol) were added to a 10 mL Schlenk tube under a nitrogen atmosphere. The tube was then sealed with a screw cap, and the reaction mixture was stirred for 2 h at 100 °C. After this time, the reaction mixture was analyzed by ^1^H NMR using 1,3,5-trimethoxybenzene as an internal standard. Product formation was quantified by comparison with previously reported data.^29^

### Single-Crystal X-ray Diffraction

Suitable crystals for X-ray Diffraction studies were obtained for complexes **2** and **4** by slow crystallization from a THF/hexane solution at low temperatures under an inert atmosphere. Single crystals were placed on a Fomblin (polyfluoro ether oil) droplet and selected and then mounted on a nylon loop. The X-ray diffraction data was collected at 113 K on a Bruker D8 Venture diffractometer equipped with a Photon II detector, using graphite monochromated Mo–Kα radiation (λ = 0.71073 Å). The data was processed using the APEX4 suite software package, which includes integration and scaling (SAINT), absorption corrections (SADABS),^39^ and space group determination (XPREP). Structure solution and refinement were done using direct methods with the programs SHELXT 2018/2 and SHELXL (version 2019/2)^40,41^ inbuilt in APEX and WinGX—Version 2023.1 software packages.^39^ Absorption correction was performed by using a multiscan procedure. All non-hydrogen atoms were refined anisotropically. Hydrogen atoms were added in idealized positions and refined with riding constraints. Atom H20 of complex **4**, the hydride was located from Fourier differences map and refined with the restrained isotropic thermal parameter (Uiso­(H) = 0.1). The Platon SQUEEZE routine^42^ was used for complex **2** in a second THF molecule, which could not be modeled. Molecular diagrams were drawn with Mercury.^43^ Crystal and structure refinement data are listed in Table S4. Crystallographic data have been deposited in the Cambridge Crystallographic Data Centre (CCDC) and FIZ Karlsruhe deposition service as CCDC 2469746 and 2469747. These can be obtained free of charge from the Cambridge Crystallographic Data Centre via www.ccdc.cam.ac.uk/data_request/cif.

### Computational Details

DFT calculations were performed with Gaussian 16 using an ultrafine integration grid (int = ultrafine).^44^ Geometry optimizations and frequency calculations were performed using the M06l functional^45^ with LANL2DZ (Mn) and def2SVP (C, H, N, O, Br) basis set, with solvent corrections (PCM, thf, ε = 7.4257) and an empirical dispersion correction (Grimme, GD3). Frequency analyses for all stationary points were performed by using the enhanced criteria to confirm the nature of the structures as either minima (no imaginary frequency) or transition states (only one imaginary frequency). The electronic energies of the optimized geometries were calculated using the M06l functional with def2tzvp for all atoms at 373 K. The Gibbs free-energy correction from the frequency calculation was added to this electronic energy to generate Gibbs free-energy values for the calculated stationary points. Intrinsic reaction coordinate (IRC) calculations were used to connect transition states and minima located on the potential energy surface allowing a full energy profile (calculated at 373 K, 1 atm) of the reaction to be constructed.^46^ Functional testing was performed with the B3LYP^47^ and ωB97XD functionals with def2tzvp basis set for all atoms at 373 K, with solvent corrections (PCM, thf, ε = 7.4257) and an empirical dispersion correction (Grimme, GD3: B3LYP).

## Supplementary Material



## Footnotes

a Experimental studies confirm that the isolated hydride complex **4** is catalytically active only in the presence of *t*-BuOK, which supports the proposed **Int3** as the active species (Tables S2 and S3).

b Imine formation occurs simply by heating benzaldehyde at 100 °C, without requiring either a base or catalyst (Table S1).

## Notes

^1^H, ^13^C NMR spectra of all compounds, IR and HRMS spectra, crystallographic data for complexes **2** and **4**, catalytic experiments, and computational studies (PDF)
