# Supplementary material for: Methylene-Linked Triazolylidenes as Cooperative Ligands in Manganese-Catalyzed N‑Alkylation
Source: Inorg Chem. 2026 Apr 16;65(16):9265–73. doi: 10.1021/acs.inorgchem.6c01357 (PMC13126626; doi:10.1021/acs.inorgchem.6c01357)
Supplement: Supplementary file 1 [file ic6c01357_si_001.pdf]

Supporting Information

Methylene-Linked Triazolylidenes as Cooperative Ligands in Manganese-Catalyzed *N*-Alkylation

Maria Batuecas,*[a,b] Beatriz Garcia,[a] Chiara Saviozzi,[a,c] Maria S. Viana,[a] Feliu Maseras,[d] and Beatriz Royo*[a]

[a] Dr. M. Batuecas, B. Garcia, Dr. C. Saviozzi, M. Viana, Dr. B. Royo,
Instituto de Tecnologia Química e Biológica António Xavier, ITQB NOVA,
Universidade Nova de Lisboa, Avenida da República, 2780-157 Oeiras (Portugal)
E-mail: broyo@itqb.unl.pt

[b] Dr. M. Batuecas
Departamento de Química Inorgánica – Instituto de Síntesis Química y Catálisis Homogénea (ISQCH),
Universidad de Zaragoza – CSIC, Facultad de Ciencias, 50009 Zaragoza (Spain)
E-mail: mbatuecas@unizar.es

[c] Dr. C. Saviozzi
Department of Chemistry and Industrial Chemistry
University of Pissa, Via G. Moruzzi, 13, I-56124, Pisa (Italy)

[d] Dr. F. Maseras
Institute of Chemical Research of Catalonia (ICIQ-CERCA), the Barcelona Institute of Science and Technology, 43007 Tarragona (Spain)

Contents

1 General Information S3

2 Synthetic Procedures S4
2.1 Synthesis of complex **2** S4
2.2 *In situ* generation of the complex **3** S4
2.3 Synthesis of complex **4** S5
2.4 *In situ* generation of the Mn hydride complex **4** S6
2.5 Imine formation S6

3 Catalytic Experiments S7
3.1 General procedure for the catalytic *N*-alkylation of *p*-toluidine with benzyl alcohol S7
3.2 Evaluation of the catalytic activity of **1**, **2**, and **4** under standard conditions S7
3.3 Evaluation of the catalytic activity of **1**, **2**, and **4** without base S7
3.4 Catalytic activity of **2** with catalytic amount of base S8
3.5 Catalyst reuse and long-term stability S9
3.6 Experimental procedure for determining KIE S11
3.7 Catalytic experiment with **4-D** S12

4 Single Crystal X-Ray Diffraction S15

5 Density Functional Theory Calculations S19
5.1 Computational methods S19
5.2 Calculated stationary points S20
5.3 Functional testing on key stationary points S24

6 NMR Spectra S25

7 Infrared Spectra S30

8 Computational Coordinates S32

9 References S90

1 General Information

All reactions and manipulations for the syntheses of ligands and metal complexes and catalytic experiments were performed under the exclusion of air and moisture using standard Schlenk techniques and a glovebox. Solvents were purified using appropriate drying agents and stored under molecules sieves under nitrogen atmosphere. Deuterated solvents were degassed and stored over molecular sieves. All other reagents were purchased from commercial suppliers and used without further purification. Infrared spectra were recorded on samples as KBr pellets using a Bruker IFS 66/S or as solids using an ATR-FTIR spectrometer. ^{1}H and ^{13}C NMR spectra were recorded on a Bruker Avance III 300 MHz, Bruker Avance III 400 MHz and Bruker Avance III 800 MHz spectrometers. Chemical shifts are expressed as δ (parts per million) relative to residual solvent signals, and *J* values are given in Hertz. Data were processed in MestReNova software. When needed, chemical shifts were assigned with the assistance of 2D NMR (HSQC, HMBC, COSY) spectra. The preparation of the Mn complex **1** was performed following the procedures previously reported by us.[1]

2 Synthetic Procedures

2.1 Synthesis of Mn complex **2**

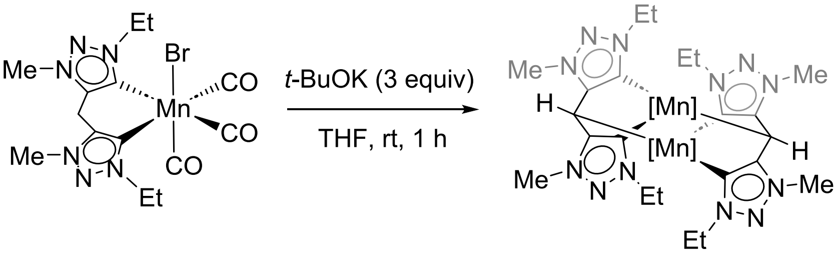

1 [Mn] = $Mn(CO)_3$ **2**

In a Schlenk flask, [Mn(bis-Trz)$(CO)_3$Br] (**1**) (100 mg, 0.22 mmol) and *t*-BuOK (72 mg, 0.66 mmol) were combined and subjected to vacuum for 10 minutes. Subsequently, dry THF (5 mL) was added, and the resulting mixture was stirred at room temperature for 1 hour. The dark red solution was then filtered through a pad of Celite, and the filtrate was concentrated to approximately 0.3 mL. Addition of hexane induced the precipitation of a brown solid, which was collected and washed with hexane (2 x 3 mL), affording complex **2** as a brown solid in 66% yield (54 mg, 0.072 mmol). Single crystals suitable for X-ray diffraction analysis were obtained by layering hexane over a concentrated THF solution of **2** and storing the mixture at -20 ºC for one week.

^{1}H NMR (400 MHz, 298 K, THF-d_8): δ 4.44 (m, 8H, 4 x C$H_2$$CH_3$), 4.02 (s, 12H, 4 x NC$H_3$), 3.58 (s, 2H C*H*, overlapping signal with THF-d_8), 1.40 (t, $^{3}J_{HH}$ = 7.7 Hz, 12H, 4 x CH_2CH_3).

^{13}C{^{1}H} NMR (201 MHz, 298 K, THF-d_8): δ 225.9 (4 x *C*O), 224.7 (2 x *C*O), 170.9 (2 x *C*-Mn), 158.7 (4 x C_{trz}-NCH_3), 48.3 (4 x *C*H_2CH_3), 35.4 (4 x N*C*H_3), 16.7 (4 x CH_2*C*H_3), 14.1 (2 x *C*H).

Selected IR data (KBr): ν (CO) 1955 s, 1862 s, 1829 s cm^{-1}.

Note: Due to its extreme sensitivity, further characterization of complex **2** was not possible.

2.2 *In Situ* generation of the Mn complex **3**

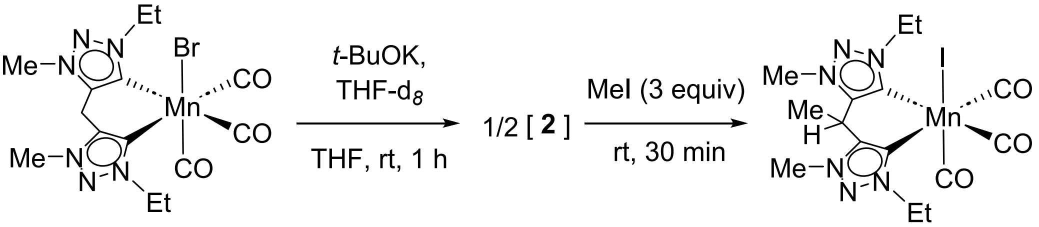

1 **3**

[Mn(bis-Trz)$(CO)_3$Br] (**1**) (10 mg, 0.022 mmol) and *t*-BuOK (7.2 mg, 0.066 mmol) were placed in a NMR tube inside a glovebox. The NMR tube was sealed and removed from the glovebox,

after which THF-d_8 (0.4 mL) was added. The reaction mixture was allowed to stand at room temperature for 1.5 hours. A ^{1}H NMR spectrum was then recorded, confirming the formation of **2**. Subsequently, MeI (2.8 µL, 0.044 mmol) was added via syringe, and the reaction was kept at room temperature for 30 minutes. A ^{1}H NMR spectrum recorded after this time confirmed the complete formation of **3**. Compound **3** was characterized by NMR and IR spectroscopy.

^{1}H NMR (300 MHz, 298 K, THF-d_8): δ 5.01 and 4.88 (both m, 2H each, 2 x CH_2CH$_3$), 4.75 (q, $^3J_{HH}$ = 6.3 Hz, 1H, CH$_3$CH), 4.22 (s, 6H, 2 x NCH_3),1.64 (overlapping signals, 3H, CH_3CH) 1.62 (overlapping signals, 6H, 2 x CH$_2$CH_3).

^{13}C{^{1}H} NMR (75 MHz, 298 K, THF-d_8): δ 176.3 (C-Mn), 145.9(2 x C$_{trz}$-NCH$_3$), 50.5 (2 x CH$_2$CH$_3$), 36.5 (2 x NCH$_3$), 28.5.9 (CH$_3$$C$H), 20.0 ($CH_3$CH), 16.1 (2 x CH$_2$$CH_3$).

Selected IR data (ATR): ν (CO) 1984 s, 1871 s cm^{-1}.

2.3 Synthesis of Mn complex **4**

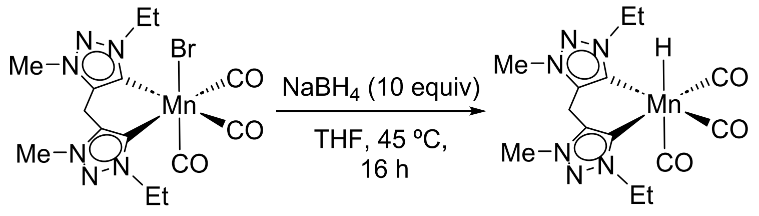

1 **4**

[Mn(bis-Trz)(CO)$_3$Br] (**1**) (50 mg, 0.11 mmol) and NaBH$_4$ (42 mg, 1.11 mmol) were added to a Schlenk flask and placed under vacuum for 10 minutes. Dry THF (4 mL) was then added, and the mixture was stirred at 45 ºC for 12 hours. The resulting red solution was filtered through a pad of Celite, and the filtrate was concentrated to approximately 0.3 mL: Hexane was then added to induce precipitation of a dark red solid. The solid was collected by filtration and washed with hexane (2 x 3 mL), affording **4** in 61% yield (25 mg, 0.067 mmol). Crystals suitable for X-ray diffraction analysis were obtained by layering hexane over a THF concentrated solution of **4** and storing the mixture at -20 ºC for one week.

^{1}H NMR (400 MHz, 298 K, THF-d_8): δ 4.65 (m, 4H, 2 x CH_2CH$_3$), 4.02 (overlapping signals, 6H, 2 x NCH_3 and 2H -CH_2-), 1.53 (t, $^3J_{HH}$ = 7.2 Hz, 6H, 2 x CH$_2$CH_3), -6.44 (s, 1H, MnH).

^{13}C{^{1}H} NMR (100 MHz, 298 K, THF-d_8): δ 188.4 (C-Mn), 141.8 (2 x C$_{trz}$-NCH$_3$), 48.4 (2 x CH$_2$CH$_3$), 35.7 (2 x NCH$_3$), 22.9 (-CH$_2$-) 16.7 (2 x CH$_2$$CH_3$).

Selected IR data (KBr): ν (CO) 1998 s, 1887 s cm^{-1}.

HR-MS (ESI$^+$, *m/z*): calc. for $C_{14}H_{18}MnN_6O_3$, [M–H]$^+$ = 373.0821; found = 373.0778.

2.4 *In situ* generation of the Mn hydride complex **4**

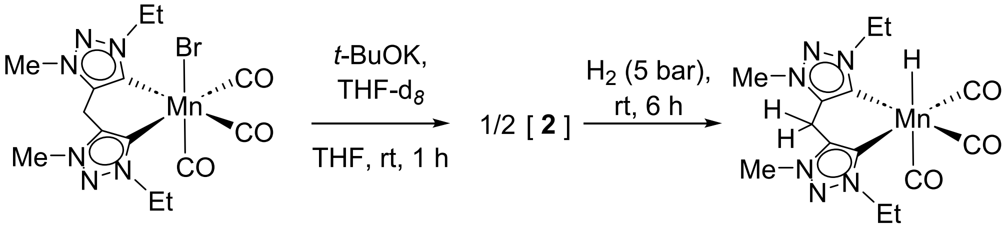

1 **4**

[Mn(bis-Trz)(CO)$_3$Br] (**1**) (10 mg, 0.022 mmol) and *t*-BuOK (7.2 mg, 0.066 mmol) were placed in a J-Young NMR tube inside a glovebox. The tube was sealed and removed from the glovebox and THF-d_8 (0.4 mL) was added. The reaction mixture was left to stand at room temperature for 1.5 hours, after which a ^{1}H NMR spectrum was recorded, confirming the formation of deprotonated intermediate **2**. The solution was then frozen by immersing the tube in liquid nitrogen. The headspace was evacuated, and H_2 gas (~ 5 bar) was introduced. The reaction was monitored by ^{1}H NMR spectroscopy until full conversion to the Mn hydride **4** was observed after 6 hours.

2.5 Imine formation

p-Toluidine (0.5 mmol), benzyl alcohol (0.75 mmol) and the corresponding additive, were added to a 10 mL Schlenk tube under a nitrogen atmosphere. The tube was then sealed with a screw cap, and the reaction mixture was stirred for 2 hours at 100 °C. After this time, the reaction mixture was analysed by ^{1}H NMR using 1,3,5-trimethoxybenzene as internal standard. Product formation was quantified by comparison with previously reported data.[1]

Imine formation under different conditions

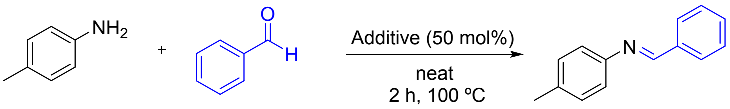

Table S1. Imine formation

Additive	Yield (%)
t-BuOK	>99
t-BuOH	>99
-	>99

3 Catalytic Experiments

3.1 *General Procedure for the Catalytic N-Alkylation of p-Toluidine with Benzyl Alcohol*. The corresponding manganese complex (1.5 mol %), *p*-toluidine (0.5 mmol), *t*-BuOK (0.25 mmol), and benzyl alcohol (0.75 mmol) were added to a 10 mL Schlenk tube under a nitrogen atmosphere. The tube was then sealed with a screw cap, and the reaction mixture was stirred for 2 hours at 100 °C. After this time, the reaction mixture was analyzed by ^{1}H NMR using 1,3,5-trimethoxybenzene as internal standard. Product formation was quantified by comparison with previously reported data.[1]

3.2 *Evaluation of the catalytic activity of **1**, **2**, and **4** under standard conditions*

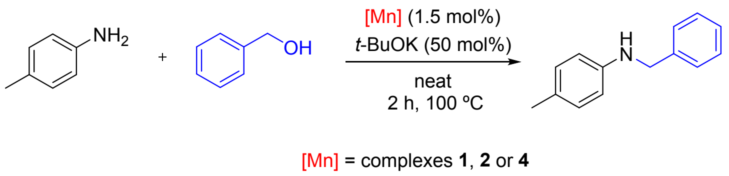

Table S2. Catalytic activity of **1**, **2**, and **4**

[Mn]	Yield (%)
**1**	>99
**2**	>99
**4**	>99

3.3 *Evaluation of the catalytic activity of **1**, **2**, and **4** without base*

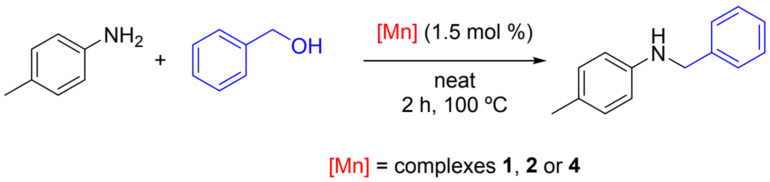

Table S3. Catalytic activity of **1**, **2**, and **4**

[Mn]	Yield (%)
**1**	0
**2**	0
**4**	0

3.4 *Catalytic activity of **2** with catalytic amount of base*

[Mn(bis-Trz)(CO)$_3$Br] (**1**) (3.4 mg, 0.0075 mmol) and *t*-BuOK (2.5 mg, 0.023 mmol) were placed in a 10 mL Schlenk tube under a nitrogen atmosphere. Subsequently, dry THF (0.2 mL) was added, and the reaction mixture turned into a red solution, confirming the formation of **2**. Then, aniline (45.7 µL, 0.5 mmol) and benzyl alcohol (78 µL, 0.75 mmol) were added, the tube was sealed with a screw cap, and the reaction mixture was stirred at 100 °C and monitored by ^{1}H NMR spectroscopy.

Time (h)	Yield (%)
0,08	2
0,17	5
0,25	10
0,33	14
0,42	17
0,50	21
0,75	31
1	39
1,25	48
1,50	54
1,75	56
2	58
3	66
4	71
5	76
6	79
7	82
10	88
17	96
20	>99

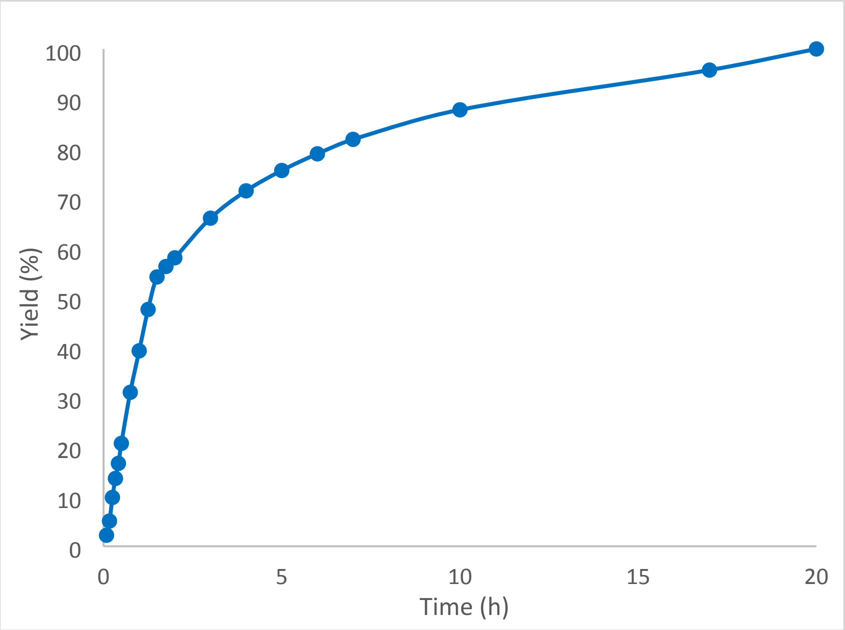

Figure S1. Kinetic profile of the *N*-alkylation of aniline with benzyl alcohol using **2** as catalyst with catalytic amount of base.

3.5 *Catalyst reuse and long-term stability*

[Mn(bis-Trz)$(CO)_3$Br] (**1**) (3.4 mg, 0.0075 mmol) and *t*-BuOK (28 mg, 0.25 mmol) were placed in a 10 mL Schlenk tube under a nitrogen atmosphere. Dry THF (0.2 mL) was then added, resulting in the formation of a red solution consistent with the *in situ* generation of complex **2**. Aniline (45.7 µL, 0.5 mmol) and benzyl alcohol (78 µL, 0.75 mmol) were subsequently added, the tube was sealed with a screw cap, and the reaction mixture was stirred at 100 °C for 2 h. The progress of the reaction was monitored by ^{1}H NMR spectroscopy.
After 2 h, fresh portions of aniline (45.7 µL, 0.5 mmol) and benzyl alcohol (78 µL, 0.75 mmol) were added, and the progress was again monitored by ^{1}H NMR spectroscopy. This procedure was repeated over four consecutive runs (cycles 1-4). The reaction mixture was then left stirring for 20 h, after which an additional portion of aniline and benzyl alcohol was introduced and the reaction was allowed to proceed for a further 2 h (cycle 5).
Note: The reaction was performed using 50 mol% of base to allow completion in 2 h. When catalytic amounts of base (4.5 mol%) are employed, full conversion requires approximately 20 h.

	time / h	yield / %		time / h	yield / %
**cycle 1**	0,0	0	**cycle 2**	0,0	0
	0,5	44		0,5	40
	1,0	78		1,0	76
	1,5	96		1,5	94
	2,0	100		2,0	100

	time / h	yield / %		time / h	yield / %
**cycle 3**	0,0	0	**cycle 4**	0,0	0
	0,5	34		0,5	30
	1,0	72		1,0	68
	1,5	84		1,5	82
	2,0	100		2,0	100

	time / h	yield / %
**cycle 5**	0,0	0
	0,5	12
	1,0	44
	1,5	68
	2,0	84

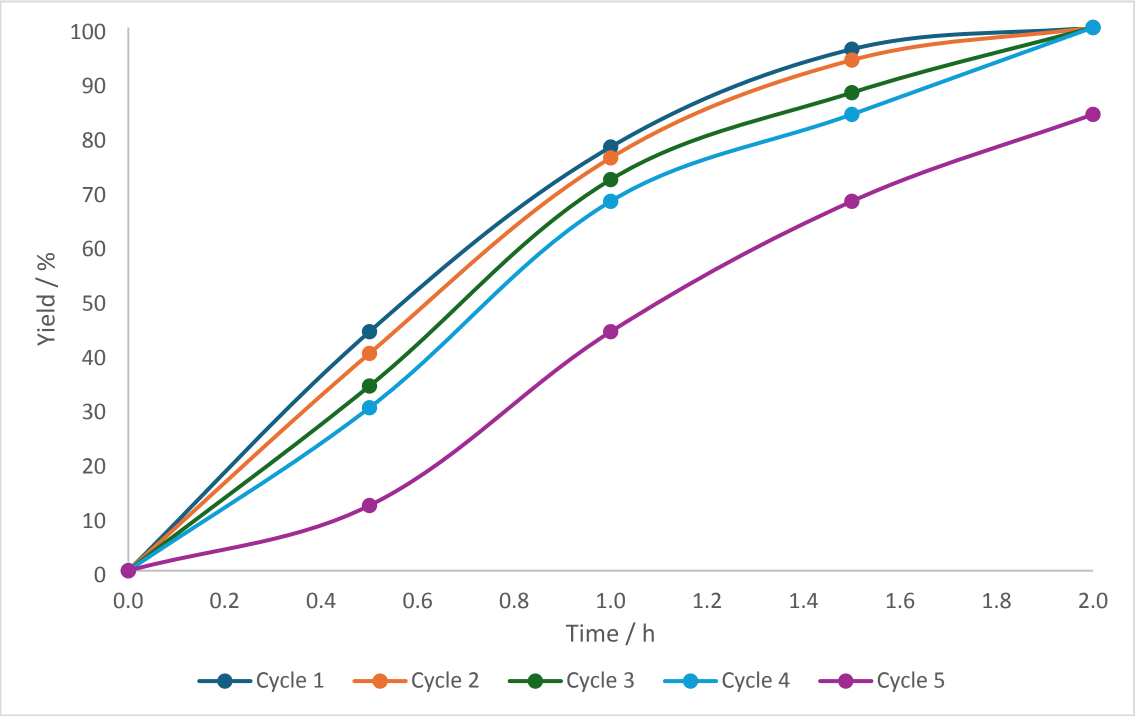

Figure S2. Stability and reuse of the Mn catalyst under consecutive catalytic cycles.

3.6 *Experimental Procedure for Determining KIE*

Parallel reactions were carried out for the *N*-alkylation of aniline using non-deuterated benzyl alcohol, benzyl-α-d alcohol, and benzyl alcohol-OD under similar conditions. [Mn(bis-Trz)$(CO)_3$Br] (**1**) (3.4 mg, 0.0075 mmol) and *t*-BuOK (2.5 mg, 0.023 mmol) were placed in a 10 mL Schlenk tube under a nitrogen atmosphere. Subsequently, dry THF (0.2 mL) was added, and the reaction mixture immediately turned into a clear red solution, confirming the formation of **2**. Then, aniline (45.7 µL, 0.5 mmol) and the corresponding alcohol (0.75 mmol) were added, the tubes were sealed with a screw cap, and placed on pre-heated (100 ºC) oil-baths, and the progress of the reaction was monitored by ^{1}H NMR spectroscopy.

benzyl alcohol-OD:

$$KIE = \frac{k_H}{k_D} = \frac{0.0039}{0.0017} \approx 2.3$$

benzyl-α-d alcohol:

$$KIE = \frac{k_H}{k_D} = \frac{0.0039}{0.0021} \approx 1.9$$

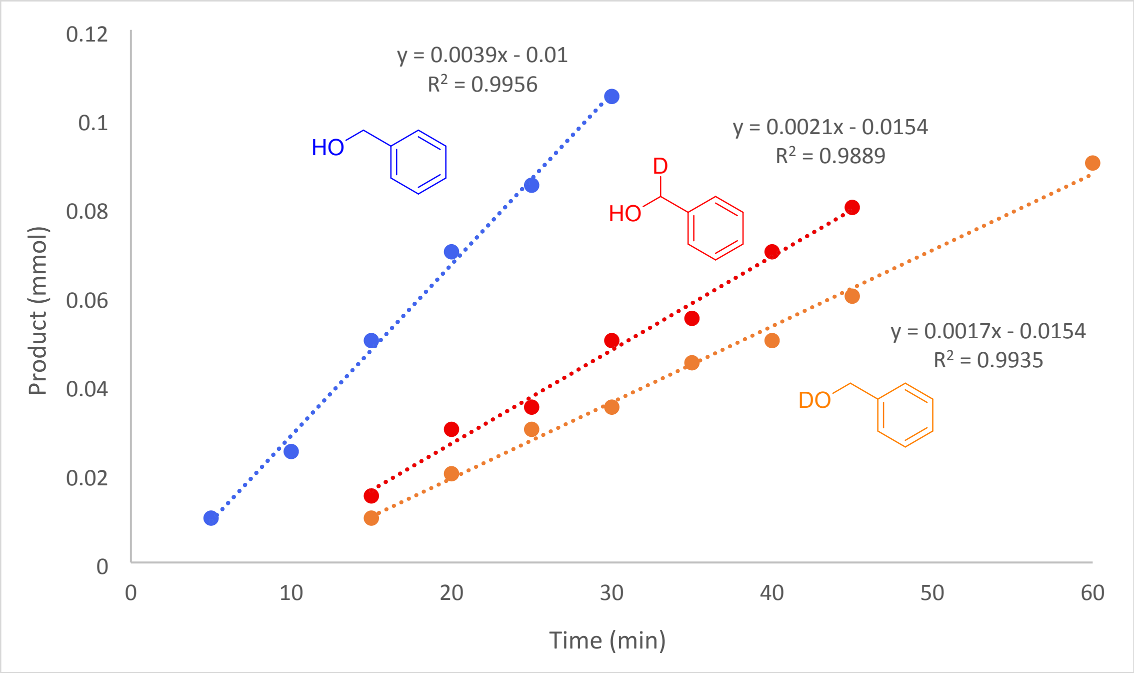

Figure S3. Concentration [Product] vs time plot for benzyl alcohol, benzyl-α-d alcohol, and benzyl alcohol-OD using **2** as catalyst.

3.7 Catalytic experiment with **4-D**

In situ generation of the deuteride Mn complex **4-D**

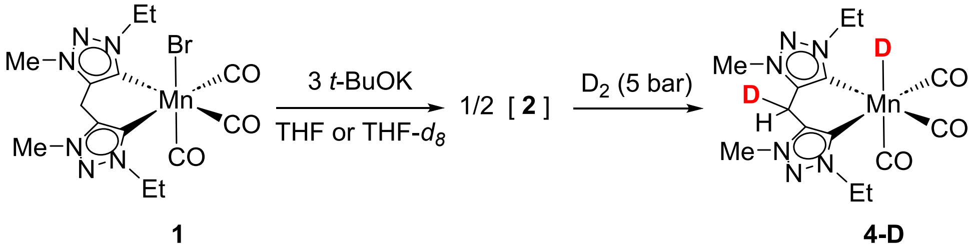

[Mn(bis-Trz)(CO)$_3$Br] (**1**) (10 mg, 0.022 mmol), *t*-BuOK (7.2 mg, 0.066 mmol) and trimethoxybenzene as internal standard (20 mg, 0.12 mmol) were placed in duplicate in two J-Young NMR tube inside a glovebox. The tubes were sealed and removed from the glovebox and THF-h_8 (0.4 mL) was added to one tube and THF-d_8 was added to the other tube. The reaction mixtures were left to stand at room temperature for 1.5 hours, after which a ^{1}H NMR spectrum of the tube with deuterated solvent was recorded, confirming the full formation of dimer **2**. The solutions were then frozen by immersing the tubes in liquid nitrogen. The headspace of both tubes was evacuated, and D_2 gas (~ 5 bar) was introduced. The reaction was monitored by ^{1}H NMR spectroscopy until no presence of complex **2** was detected in the tube with THF-d_8 as solvent. To confirm the deuterium incorporation in the formed product, a ^{2}H NMR spectrum was recorded for the reaction mixture in THF-h_8 (Figure S4).

^{2}H NMR (61.4 MHz, 298 K, THF-h_8): δ 3.94 (s, -C(H/*D*)$_2$-), -6.18 (s, Mn-*D*).

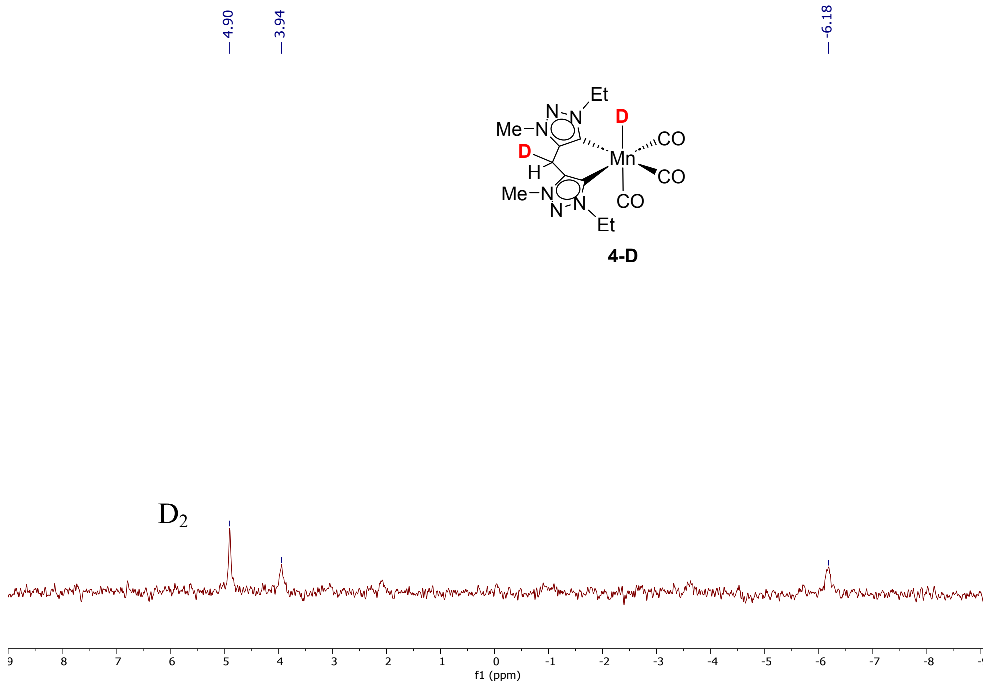

Figure S4. ^{2}H NMR spectrum of complex **4-D** in THF-h_8.

*Catalytic activity of **4-D** in THF*

Reaction mixtures in THF-h_8 and THF-d_8 containing **4-D** (ca. 0.022 mmol) were degassed by freeze-pump-thaw method 3 times. Then, aniline (10 μL, 0.11 mmol) and benzyl alcohol (17 μL, 0.16 mmol) were added to each NMR tubes under an argon atmosphere and a ^{1}H NMR spectrum of the mixture in THF-d_8 was recorded before reaction started (t0 NMR experiment). Then, the reaction mixtures were heated for 2 hours at 100 °C. After this time, the reaction mixture in THF-d_8 was analyzed by ^{1}H NMR showing full conversion to the benzylaniline. Quantitative ^{1}H NMR integration of the methylene signals of the product against signals of the internal standard, previously calibrated with the t0 NMR experiment, showed a deuterium incorporation of 30% (Figure S5). In the ^{2}H NMR spectrum of the reaction mixture in THF-h_8 a broad signal at 4.44 ppm, corresponding to the methylene fragment of the product, is observed (Figure S6).

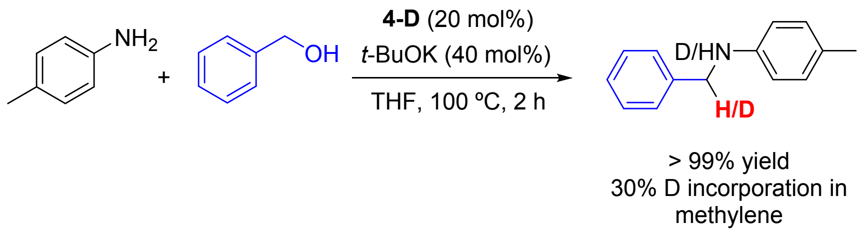

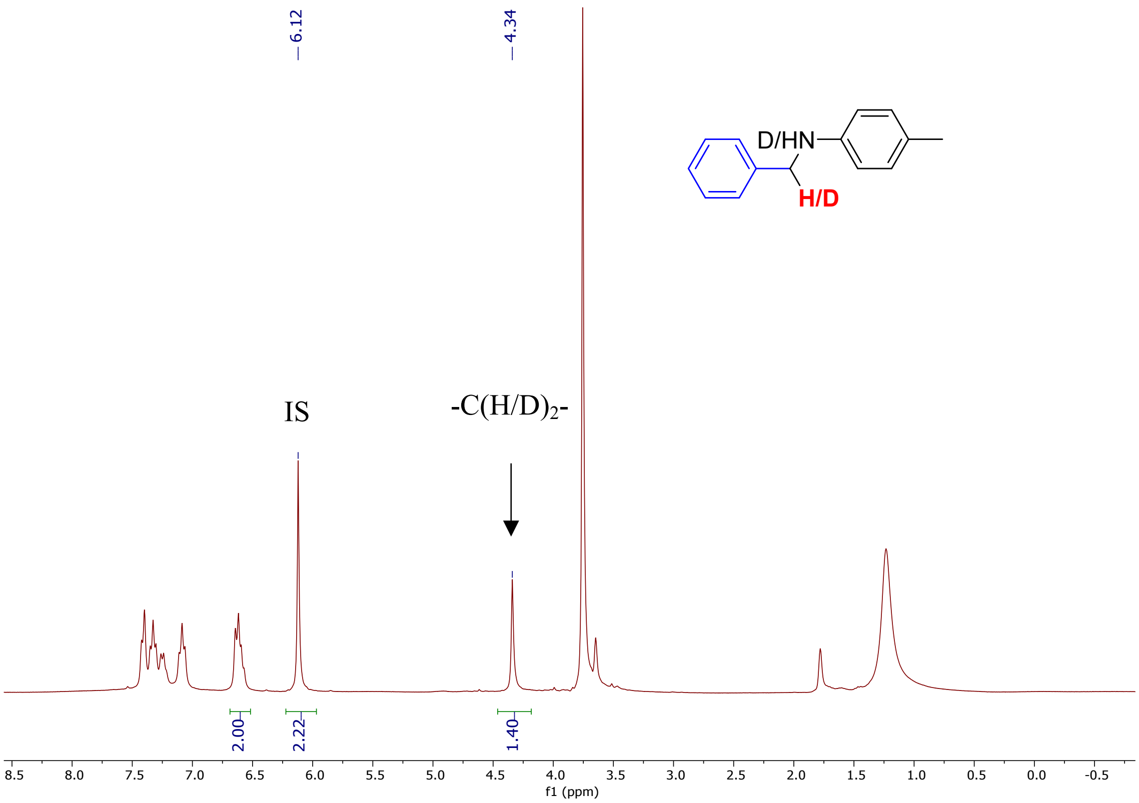

Figure S5. ^{1}H NMR spectrum the catalytic mixture using 20% mol of **4-D** as catalyst in THF-d_8.

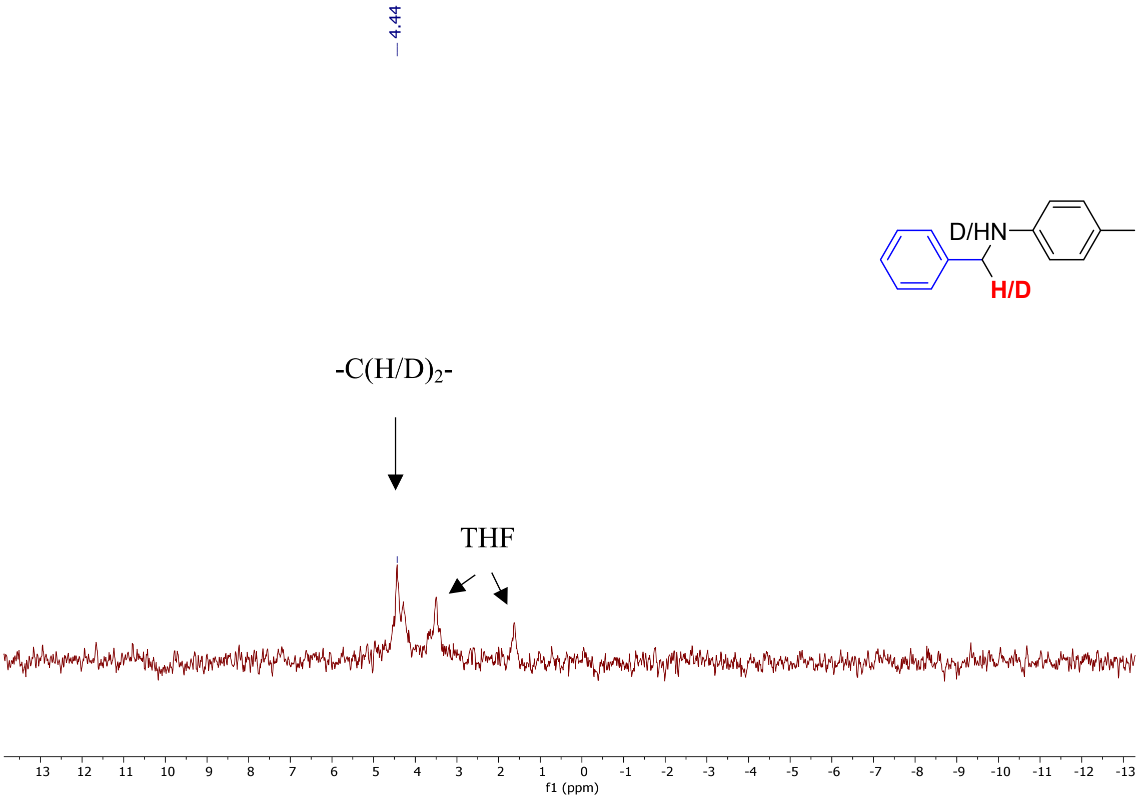

Figure S6. ^{2}H NMR spectrum the catalytic mixture using 20% mol of **4-D** as catalyst in THF-h_8. Residual signals of deuterated solvent were used as reference.

4 Single Crystal X-Ray Diffraction

Suitable crystals for X-Ray Diffraction studies were obtained for complexes **2** and **4** by slow crystallisation from a THF/hexane solution at low temperatures under inert atmosphere. Single crystals were placed on a Fomblin (polyfluoro ether oil) droplet and selected, then mounted on a nylon loop. The X-ray diffraction data was collected at 113K on a Bruker D8 Venture diffractometer equipped with a Photon II detector, using graphite monochromated Mo-Kα radiation (λ=0.71073 Å). The data was processed using the APEX4 suite software package, which includes integration and scaling (SAINT), absorption corrections (SADABS)2[2] and space group determination (XPREP). Structure solution and refinement were done using direct methods with the programs SHELXT 2018/2 and SHELXL (version 2019/2)[3,4] inbuilt in APEX, and WinGX - Version 2023.1 software packages.[2] Absorption correction was performed using a multiscan procedure. All non-hydrogen atoms were refined anisotropically. Hydrogen atoms were added in idealized positions and refined with riding constraints. Atom H20 of complex **4**, the hydride was located from Fourier differences map and refined with the restrained isotropic thermal parameter (Uiso(H) = 0.1). The Platon SQUEEZE routine[5] was used for complex **2** in a second THF molecule, which could not be modelled. Molecular diagrams were drawn with Mercury.[6] Crystal and structure refinement data are given in Table S4. Crystallographic data have been deposited in the Cambridge Crystallographic Data Centre (CCDC) and FIZ Karlsruhe deposition service as CCDC 2469746 and 2469747. These can be obtained free of charge from the Cambridge Crystallographic Data Centre viawww.ccdc.cam.ac.uk/data_request/cif.

Table S4. Crystal data and structure refinement for complexes **2** and **4**.

	2	***4***
Empirical formula	$C_{28}H_{34}Mn_2N_{12}O_6$, C_4H_8O	$C_{14}H_{19}MnN_6O_3$, C_4H_8O
Formula weight	816.65	446.39
T (K)	113(2)	113(2)
λ (Å)	0.71073	0.71073
Crystal system	Triclinic	Triclinic
Space group	*P*-1	*P*-1
a (Å)	9.8321(15)	8.734(4)
b (Å)	11.1713(18)	9.576(5)
c (Å)	20.236(3)	14.425(7)
α (°)	88.615(5)	109.152(16)
β (°)	86.520(5)	95.473(16)
γ (°)	64.899(5)	108.227(17)
Volume (Å^3)	2009.0(5)	1055.8(9)
Z	2	2
ρ_{calc} (g.cm^{-3})	1.350	1.404
μ (mm^{-1})	0.687	0.661
Crystal size (mm^3)	0.080 x 0.050 x 0.040	0.100 x 0.080 x 0.050
Crystal colour	yellow	yellow
Crystal description	prism	prism
θ_{max} (°)	25.680	26.104
Total reflections	84,999	34,294
Unique reflections	7,607	4,057

Data completeness (%)	99.9	96.8
R_{int}	0.1999	0.4398
R1	0.1214	0.1198
wR2	0.2835	0.2831
Goodness of fit	1.144	1.057
ρ_{min} and ρ_{max}	-1.145 and 1.652	-0.778 and 0.652

Both complexes **2** and **4** crystallised in a triclinic *P*-1 crystal system and space group, respectively. They adopt a distorted octahedral geometry as seen in Figures S7 and S8. Relevant distances and angles are presented in Table S5.

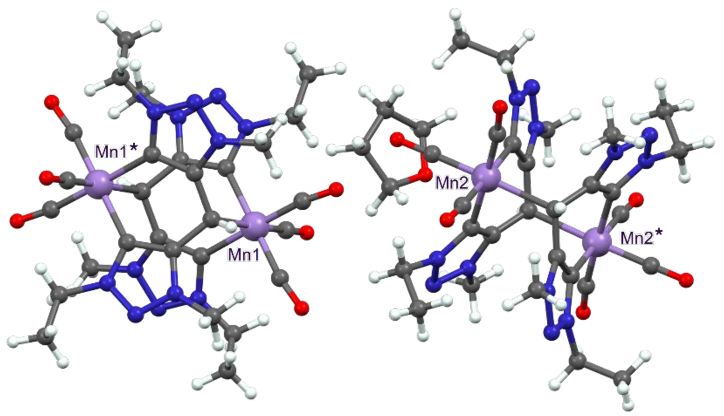

Figure S7. Single crystal structures of complex **2** with Mn atoms labelled containing two dimers and one co-crystallised THF molecule.

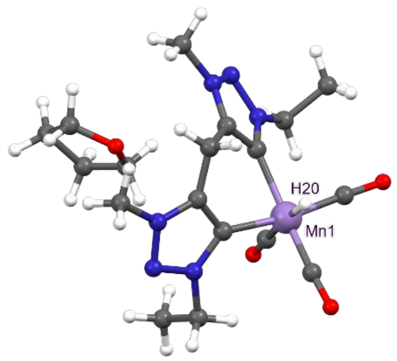

Figure S8. Single crystal structure of complex **4** with Mn and H20 atoms labelled containing one complex and one co-crystallised THF molecule.

Complex **2** shows half of two independent molecules and a co-crystallised THF in its asymmetric unit, as represented in Figure S9. The dimers are then formed by symmetry operations (1-x,1-y,1-z and -x,1-y,2-z, respectively).

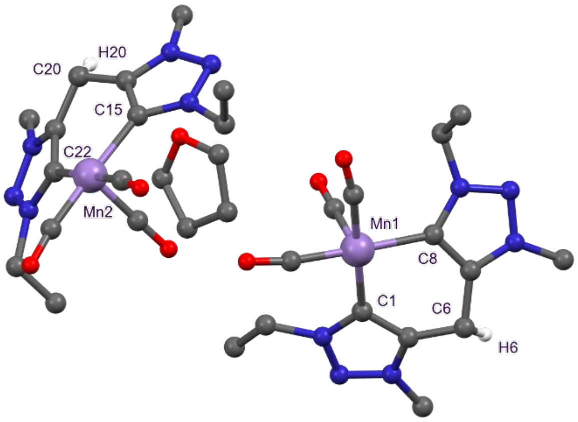

Figure S9. Asymmetric unit of complex **2** with selected atoms labelled. Non-relevant hydrogens omitted for clarity.

Table S5. Selected bond distances (Å) and angles (º), and other relevant structural parameters for complexes **2** and **4**.

	2		***4***
	(Mn1)	*(Mn2)*	
Mn-C(NHC)	2.040(8)<br>2.046(8)	2.058(9)<br>2.063(9)	2.044(8)<br>2.015(8)
Mn-CO	1.781(9)<br>1.807(9)<br>1.794(9)	1.791(10)<br>1.788(9)<br>1.789(10)	1.777(10)<br>1.785(10)<br>1.769(9)
Mn-CH(bridge)	2.270(8)	2.270(9)	---
Mn-H	---	---	1.48(5)
Mn-C-O [a]	179.6(9)<br>170.8(7)<br>171.5(7)	178.9(8)<br>173.7(8)<br>171.9(8)	176.2(7)<br>176.2(7)<br>179.0(8)
C-Mn-C[b]	83.6(3)	84.5(3)	82.7(3)
ω[c]	43.4(4)	40.6(4)	53.3(3)

[a] Angle centred at the carbon atom of carbonyl bonds. [b] Bite angle of triazolylidene ligand. [c] Dihedral angle between the triazolylidene ring planes.

Packing of complexes **2** and **4** present only non-classical hydrogen bonds. In both cases, the interactions have an aliphatic C–H as the donor. Although they co-crystallised with THF, the solvent does not have any relevant interaction with the complexes. These results are presented in Table S6 and Figures S10 (complex **2**) and S11 (complex **4**).

Table S6. Hydrogen bonds present in the packing of complexes **2** and **4**.

Complex	interaction	D···A (Å)	H···A (Å)	D-H···A (º)	Symmetry operation
**2**	(CH_2CH_3)C-H···O(CO)	3.1360(5)	2.53	119.06	1-x,1-y,1-z
	(CH_2CH_3)C-H···N(NHC)	3.3782(5)	2.55	141.46	-x,1-y,1-z

	$(CH_3)C\text{-}H\cdots N(NHC)$	3.3733(5)	2.40	170.99	1-x,1-y,-z
**4**	$(CH_2CH_3)C\text{-}H\cdots N(NHC)$	3.4929(18)	2.51	170.13	1+x,y,z

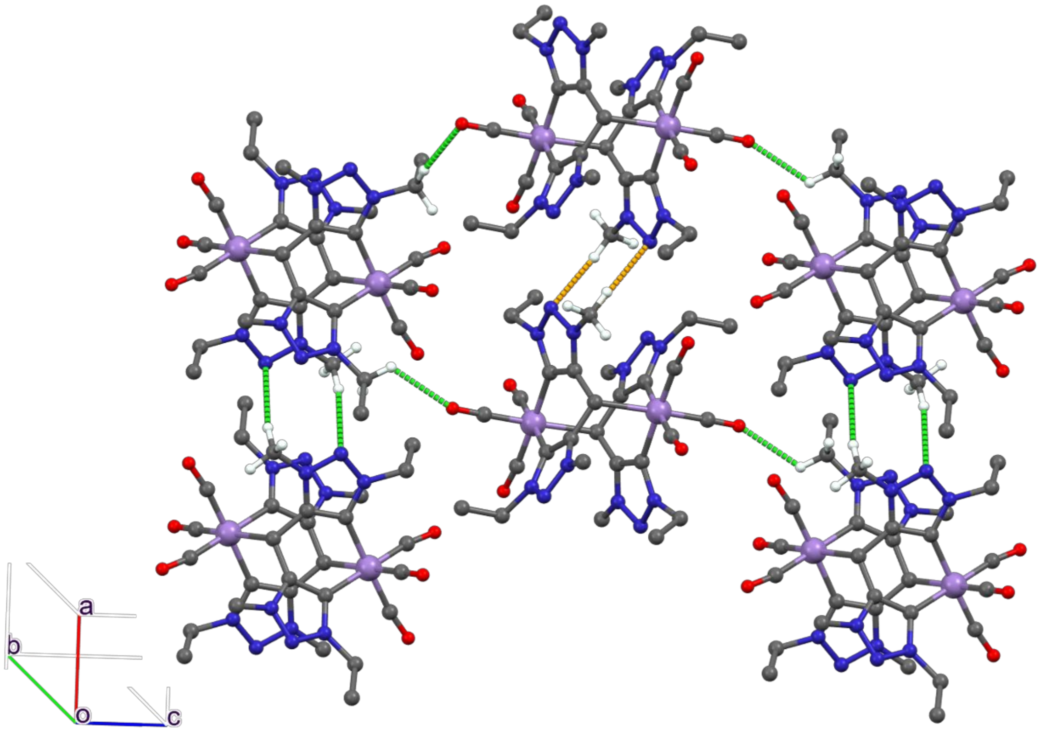

Figure S10. Mercury drawing of the interactions between neighbouring molecules in the crystal packing of complex **2**. THF molecules and non-relevant H atoms omitted for clarity. ethyl group interactions in green dashed lines and methyl group interactions in orange dashed lines.

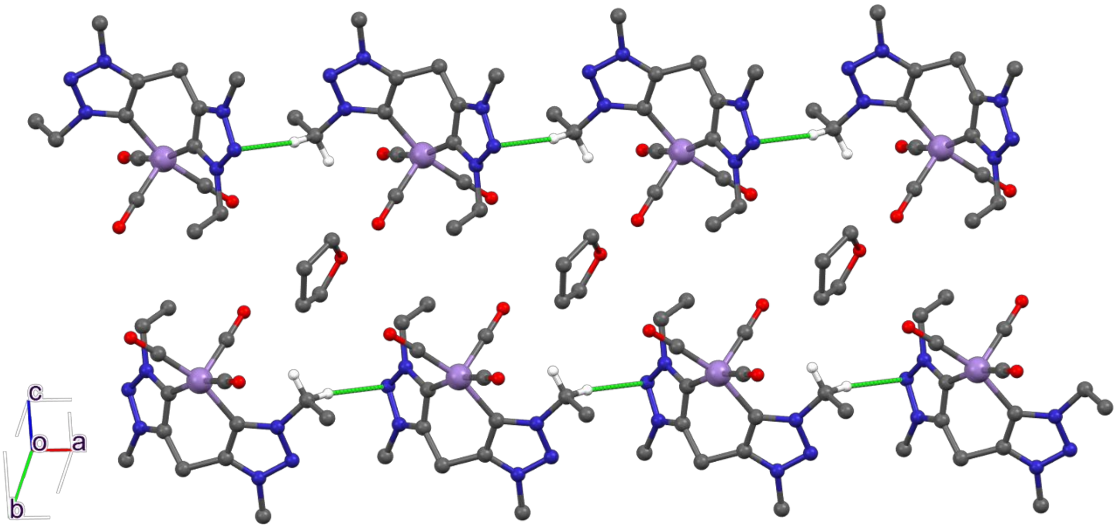

Figure S11. Mercury drawing of the interactions between neighbouring molecules in the crystal packing of complex **4**. Non-relevant H atoms omitted for clarity.

5 Density Functional Theory Calculations

5.1 Computational methods

DFT calculations were performed with Gaussian 16 using an ultrafine integration grid (int=ultrafine).[7] Geometry optimisations and frequency calculations were performed using the M06l functional[8] with LANL2DZ (Mn) and def2SVP (C, H, N, O, Br) basis set, with solvent corrections (PCM, thf, ε = 7.4257) and an empirical dispersion correction (Grimme, GD3). Frequency analyses for all stationary points were performed using the enhanced criteria to confirm the nature of the structures as either minima (no imaginary frequency) or transition states (only one imaginary frequency). The electronic energies of the optimised geometries were calculated using the M06l functional with def2tzvp for all atoms at 373 K. The Gibbs free energy correction from the frequency calculation was added to this electronic energy to generate Gibbs free energy values for the calculated stationary points.

Intrinsic reaction coordinate (IRC) calculations were used to connect transition states and minima located on the potential energy surface allowing a full energy profile (calculated at 373 K, 1 atm) of the reaction to be constructed.[9]

Functional testing was performed with the B3LYP,[10] and ωB97XD functionals with def2tzvp basis set for all atoms at 373 K, with solvent corrections (PCM, thf, ε = 7.4257) and an empirical dispersion correction (Grimme, GD3: B3LYP).

5.2 Calculated stationary points

Calculated transformation of **1** into **Int1** and **Int1A**.

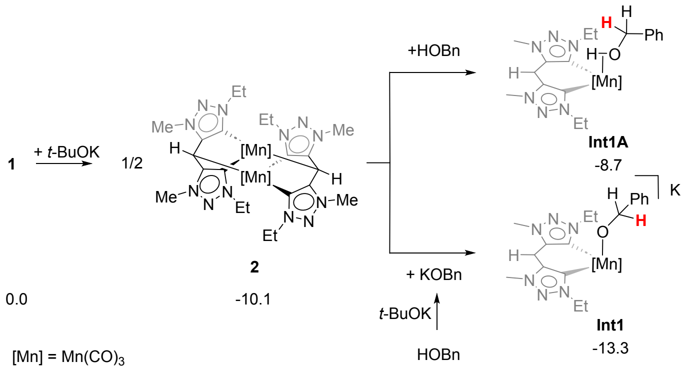

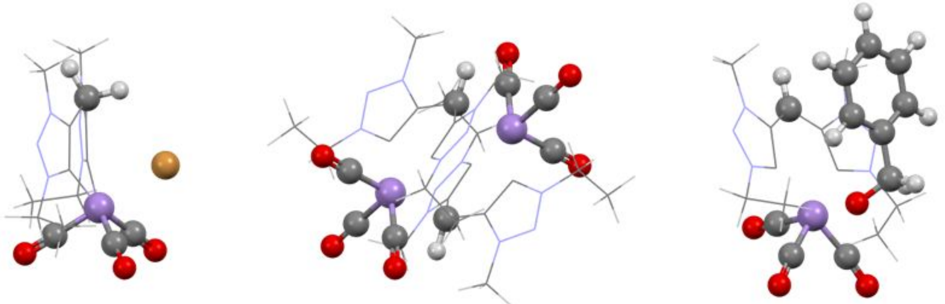

1 **2** **Int1**

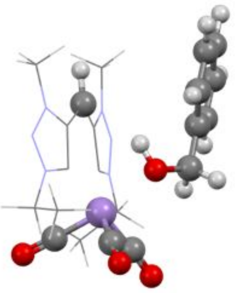

Int1A

Figure S12. Calculated transformation of **1** into **Int1** and **Int1A**. All energies in kcalmol[-1].

Calculated transformation of **Int1A** into **Int1**

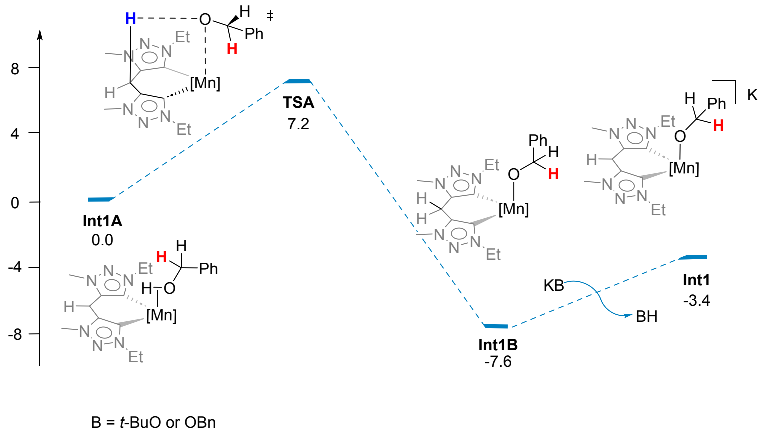

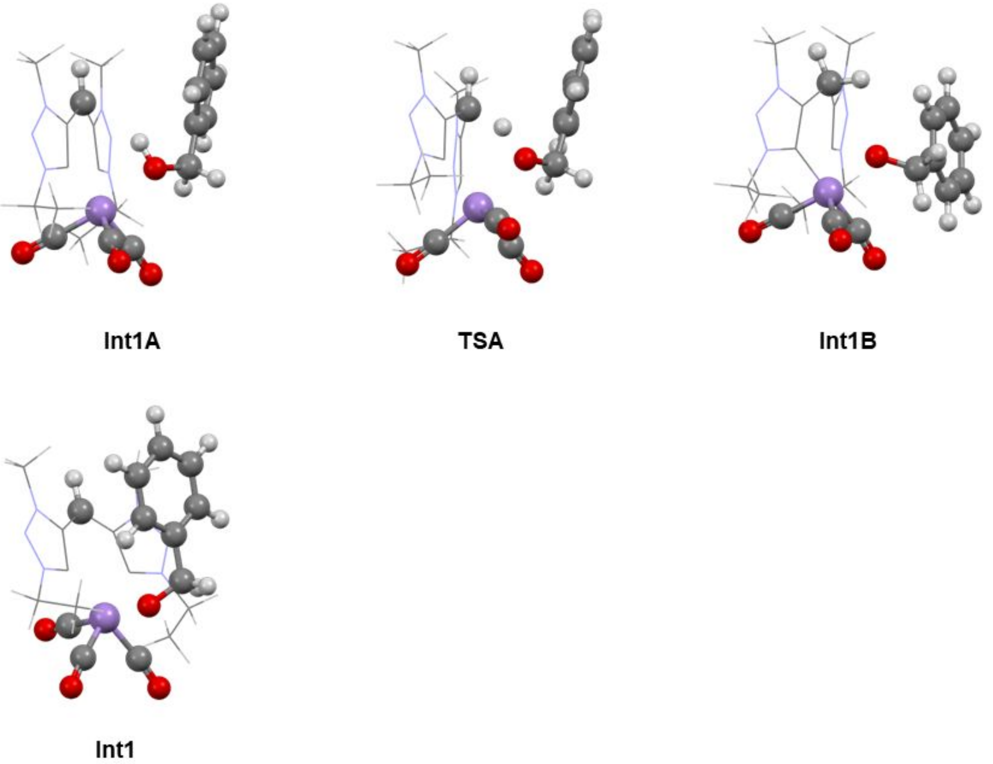

Figure S13. Calculated transformation of **Int1A** into **Int1**. All energies in kcalmol^{-1}.

Calculated mechanisms for *N*-alkylation of aniline with benzyl alcohol

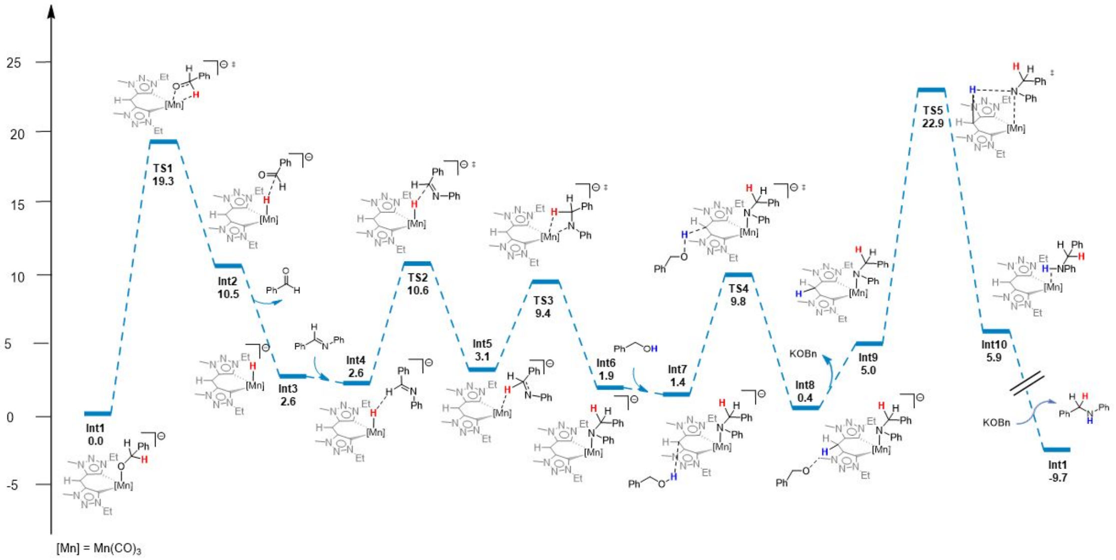

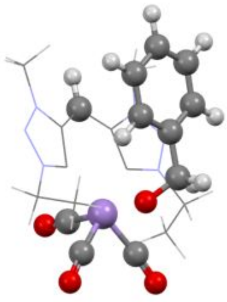

Int1

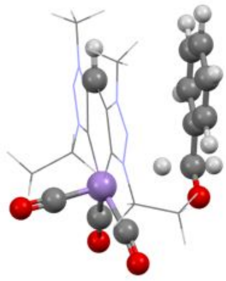

TS1

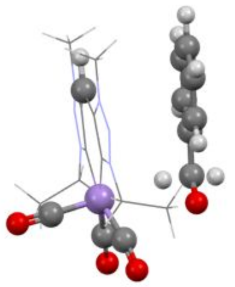

Int2

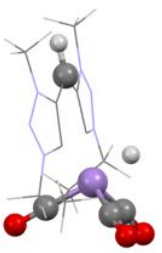

Int3

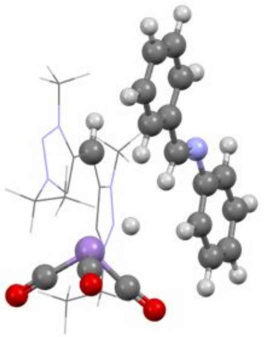

Int4

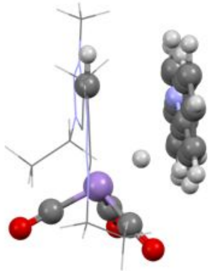

TS2

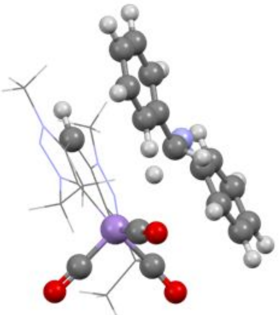

Int5

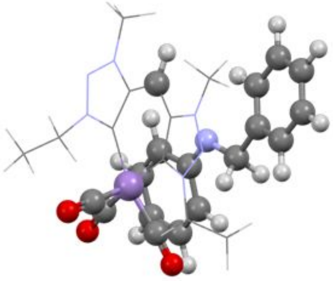

TS3

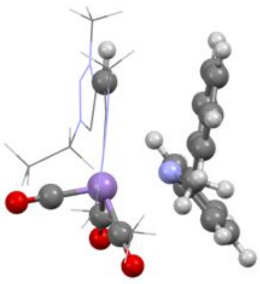

Int6

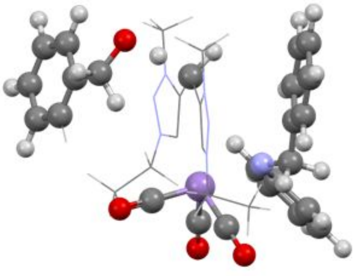

Int7

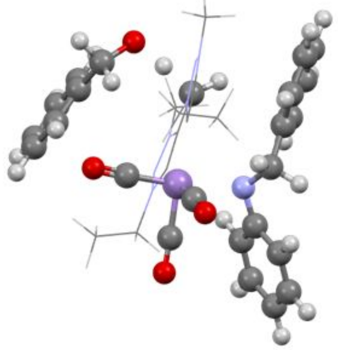

TS4

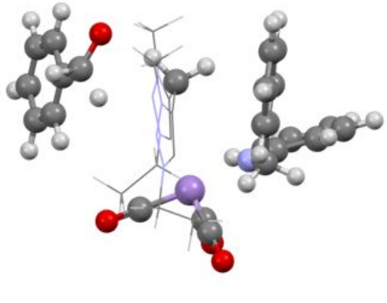

Int8

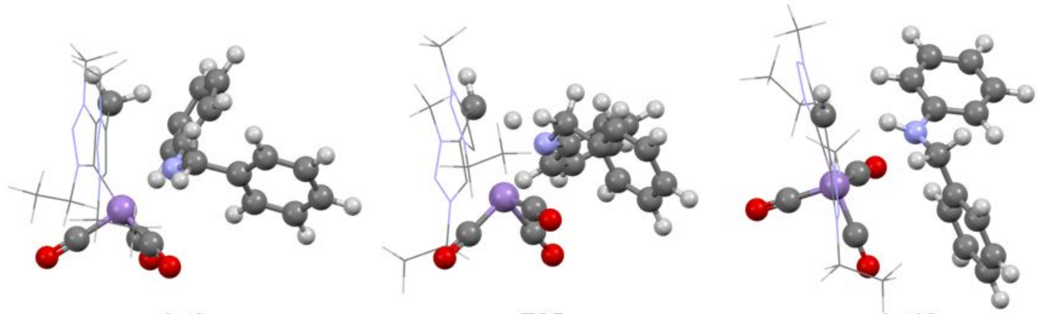

Int9 TS5 Int10

Figure S14. Calculated mechanisms for *N*-alkylation of aniline with benzyl alcohol All energies in kcalmol^{-1}.

<u>Alternative mechanism for *N*-alkylation of aniline with benzyl alcohol</u>

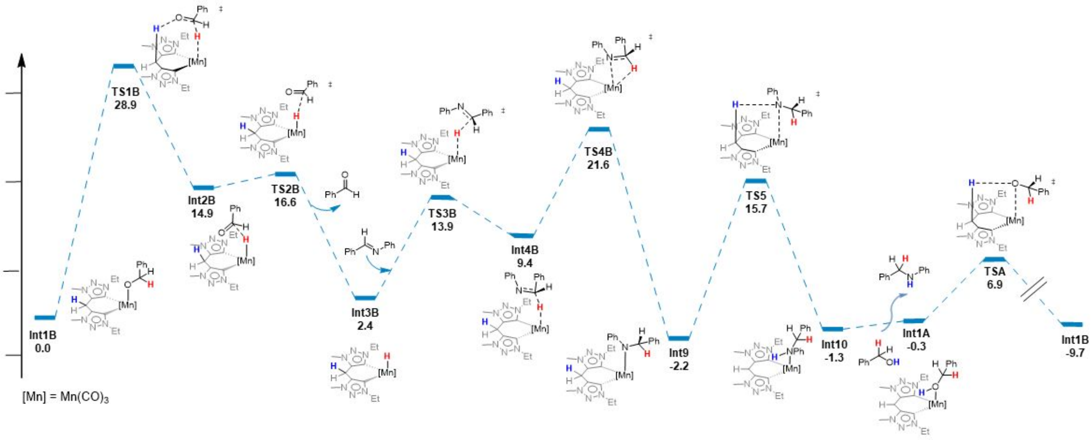

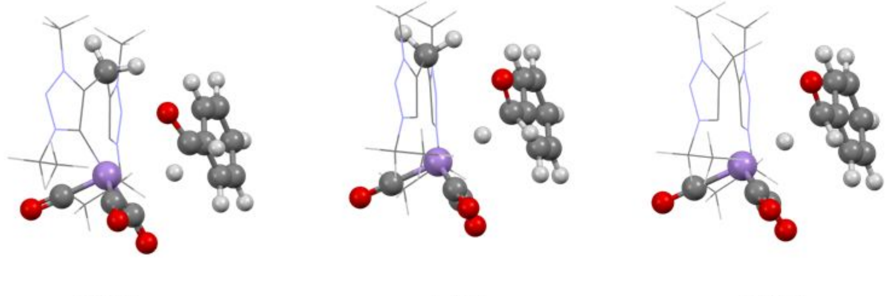

TS1B Int2B TS2B

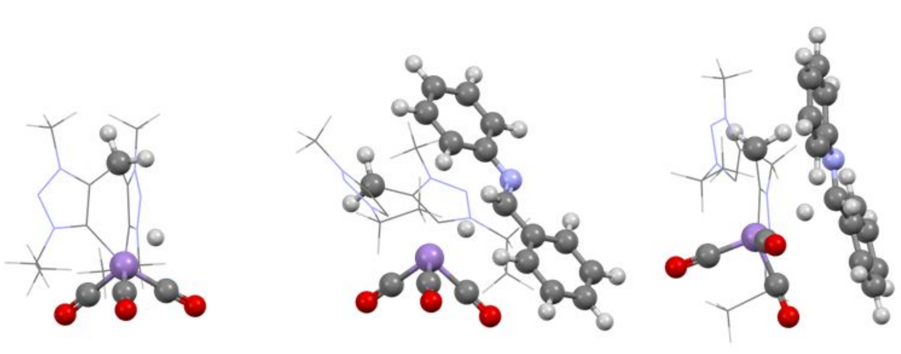

Int3B TS3B Int4B

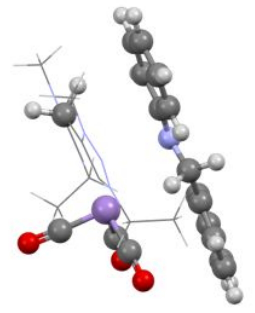

TS4B

Figure S15. Alternative calculated mechanisms for *N*-alkylation of aniline with benzyl alcohol All energies in kcalmol^{-1}.

Organic molecules

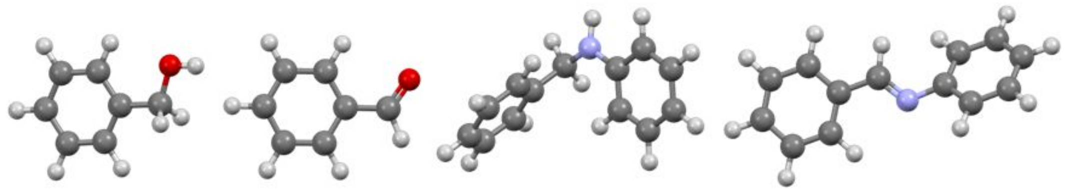

$PhCH_2OH$ PhCHO $PhCH_2NHPh$ PhCHNPh

Figure S16. Representation of the calculated organic molecules.

5.3 Functional testing on key stationary points

Functional testing was performed on key stationary points within the calculated mechanism. All Gibbs free energies provided in kcal mol^{-1} relative to **Int1**.

	**TS1**	**TS2**	**TS3**	**TS4**	**TS5**
M06L	19.3	10.6	9.4	9.8	22.9
B3LYP	16.9	9.9	7.7	9.2	16.8
WB97XD	20.0	14.6	10.5	4.7	16.8

Table S7. All Gibbs free energies provided in kcalmol^{-1}. Gibbs free energies relative to **Int1**.

6 NMR Spectra

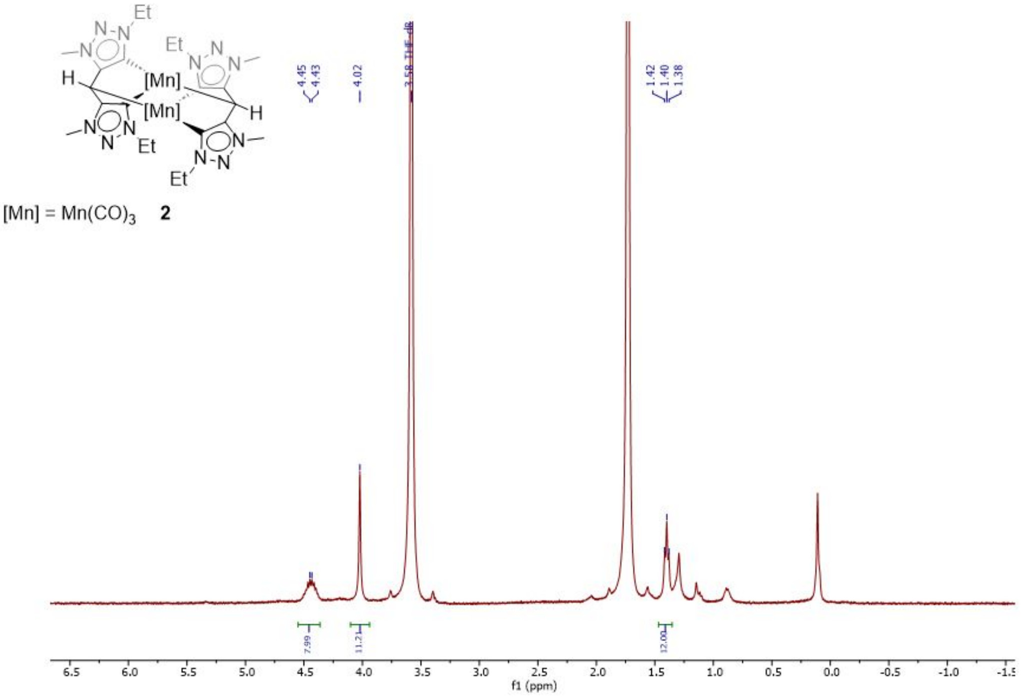

Figure S17. ^{1}H NMR spectrum (THF-d_8, 400 MHz) of **2**.

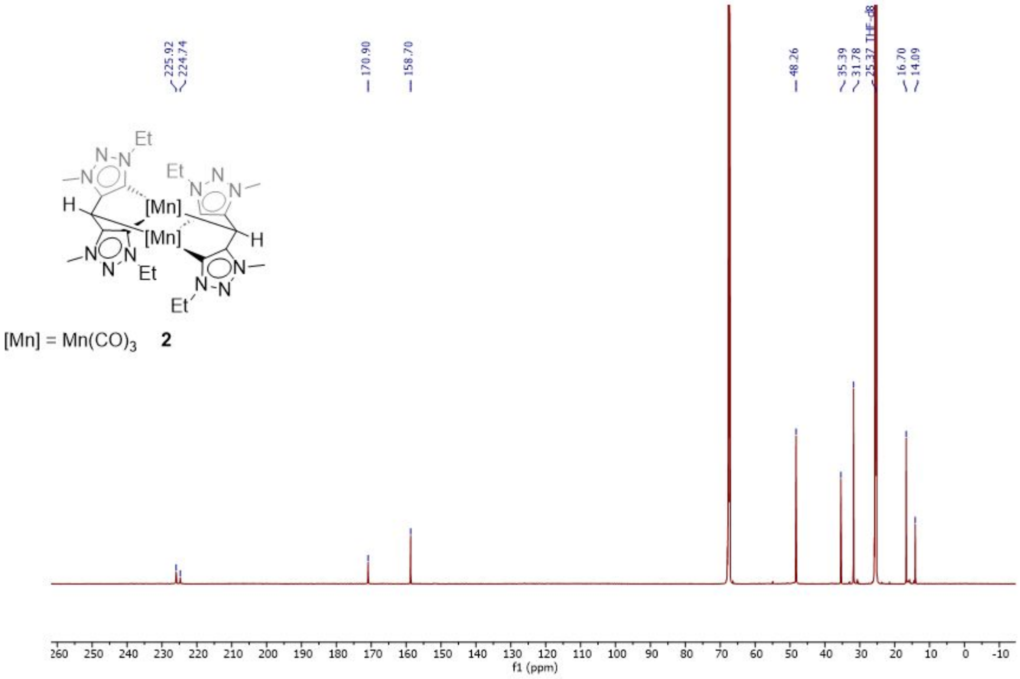

Figure S18. ^{13}C NMR spectrum (THF-d_8, 100 MHz) of **2**.

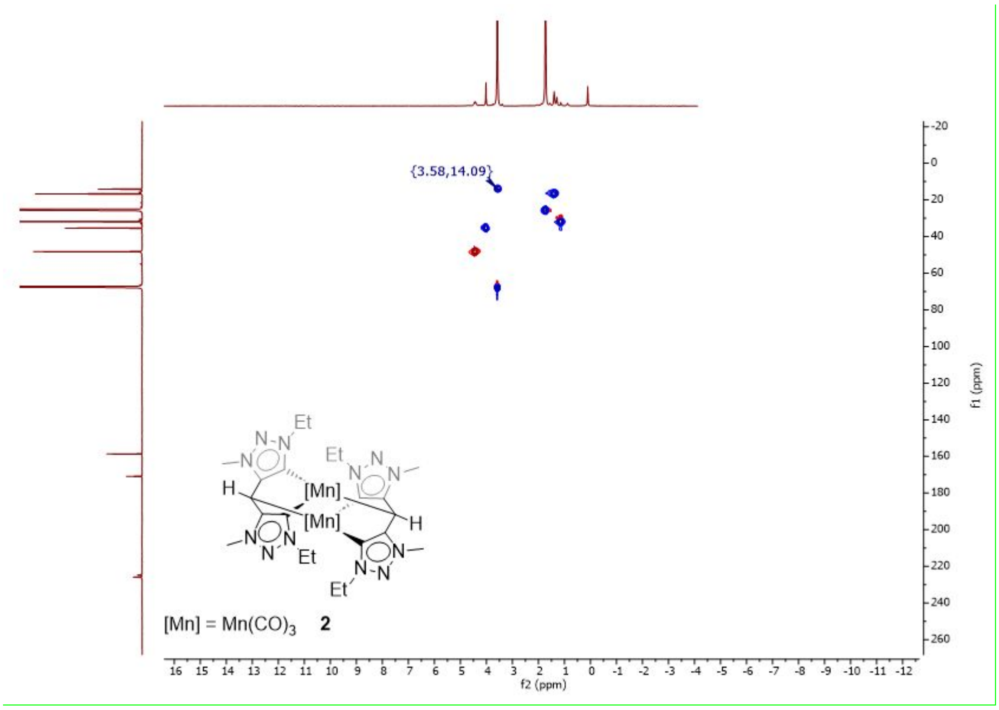

Figure S19. ^{1}H-^{13}C HSQC NMR spectrum (THF-d_8,) of **2**.

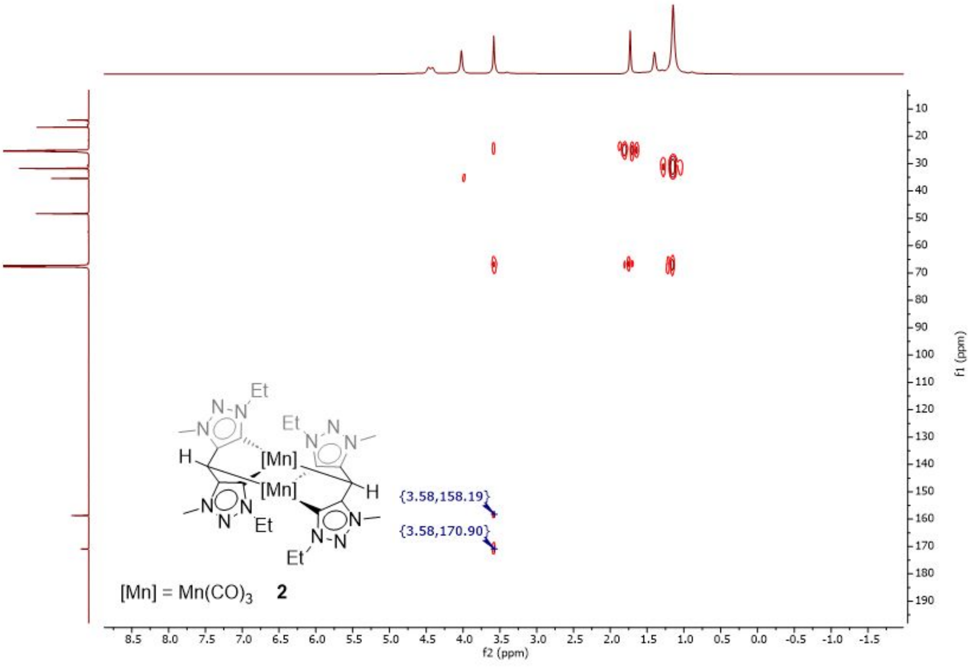

Figure S20. ^{1}H-^{13}C HMBC NMR spectrum (THF-d_8,) of **2**.

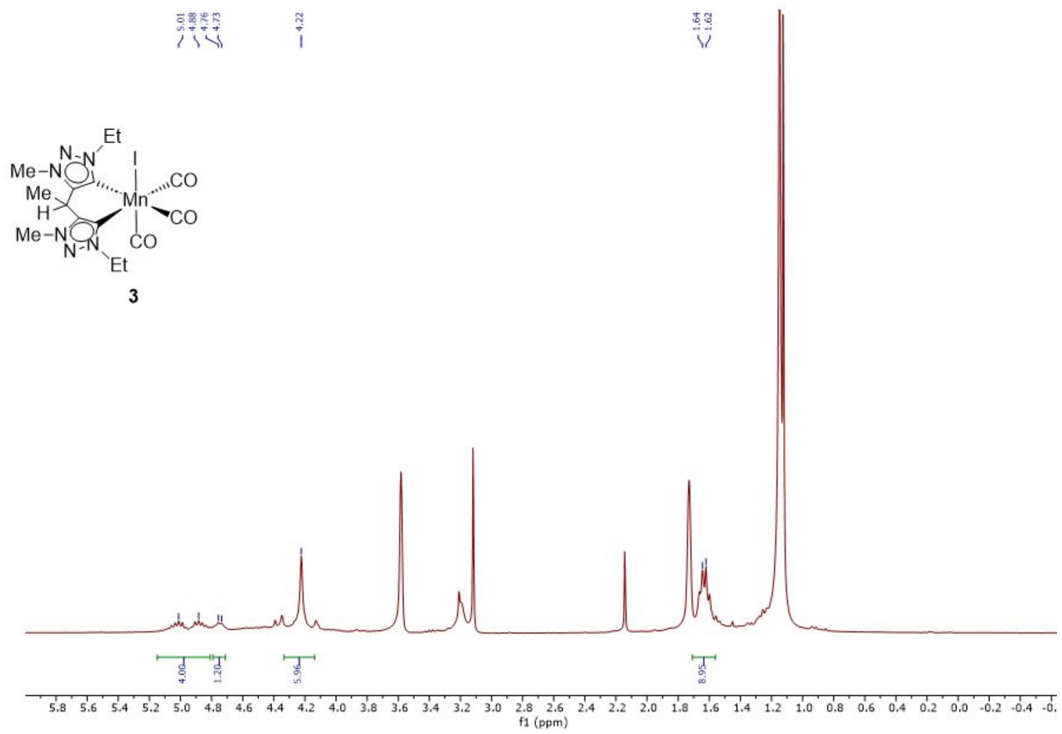

Figure S21. ^{1}H NMR spectrum (THF-d_8, 300 MHz) of **3**.

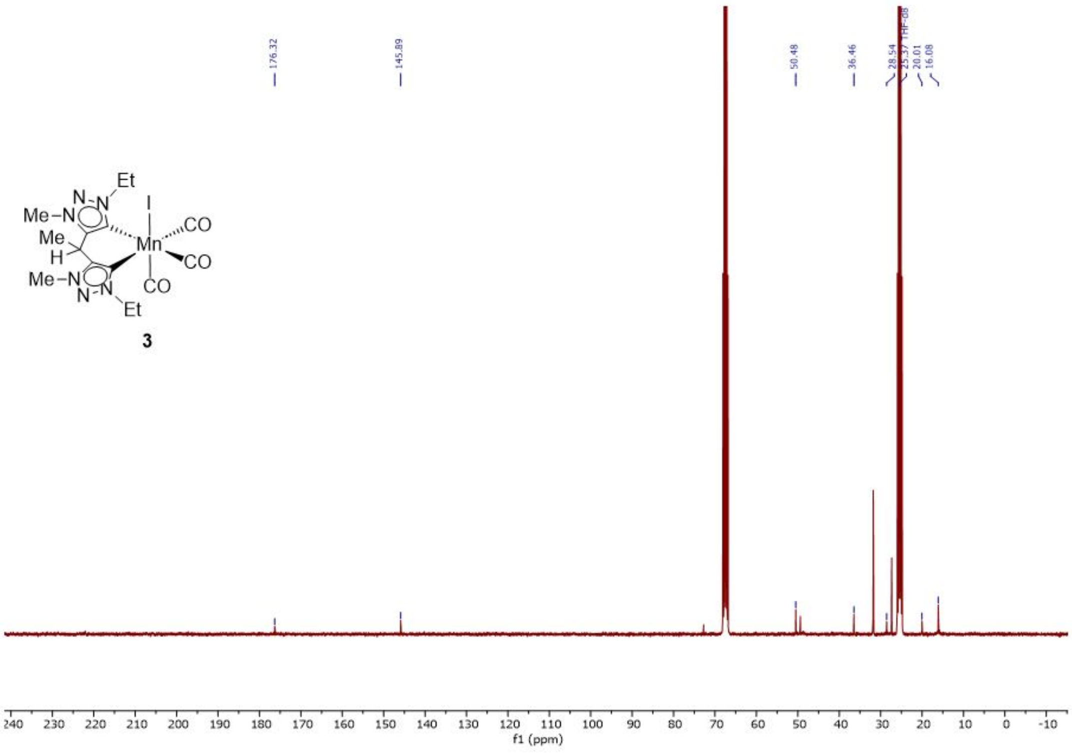

Figure S22. ^{13}C NMR spectrum (THF-d_8, 75 MHz) of **3**.

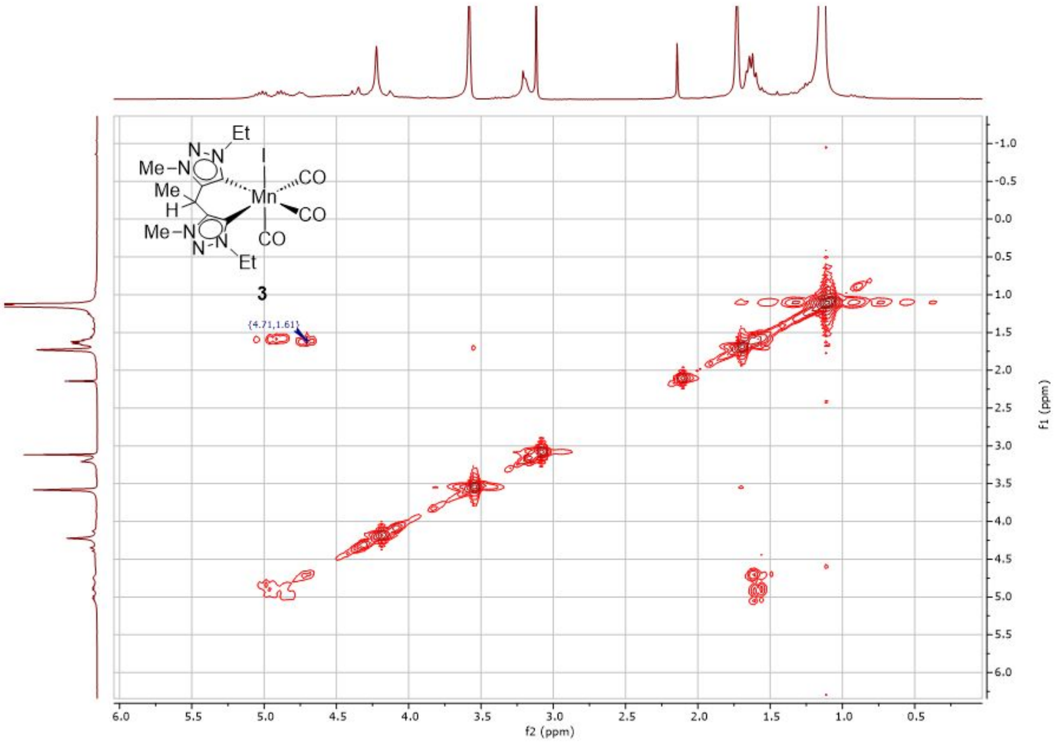

Figure S23. ^{1}H-^{1}H COSY NMR spectrum (THF-d_8) of **3**.

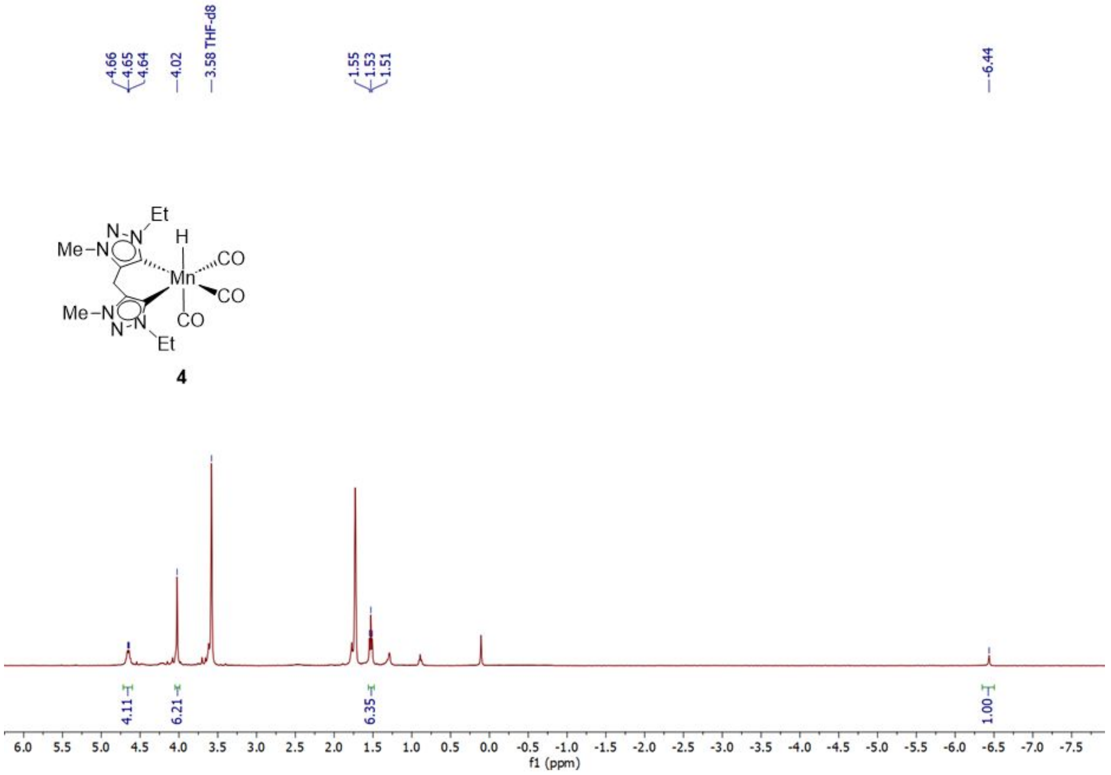

Figure S24. ^{1}H NMR spectrum (THF-d_8, 400 MHz) of **4**.

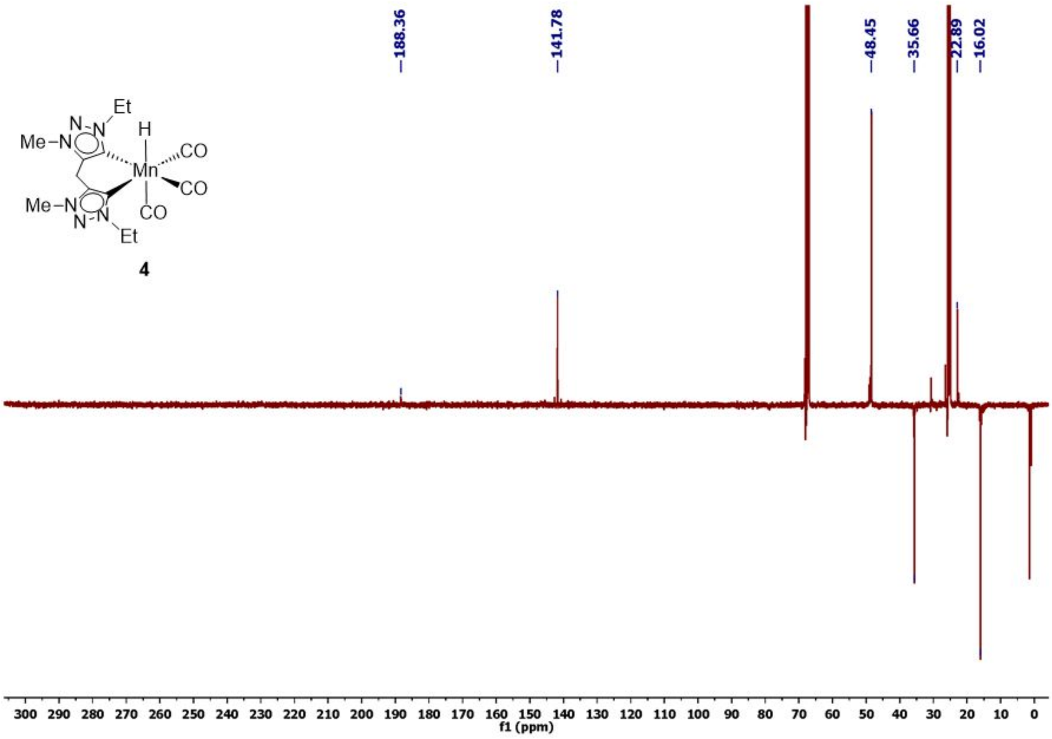

Figure S25. ^{13}C NMR spectrum (THF-d_8, 100 MHz) of **4**.

7 IR Spectra

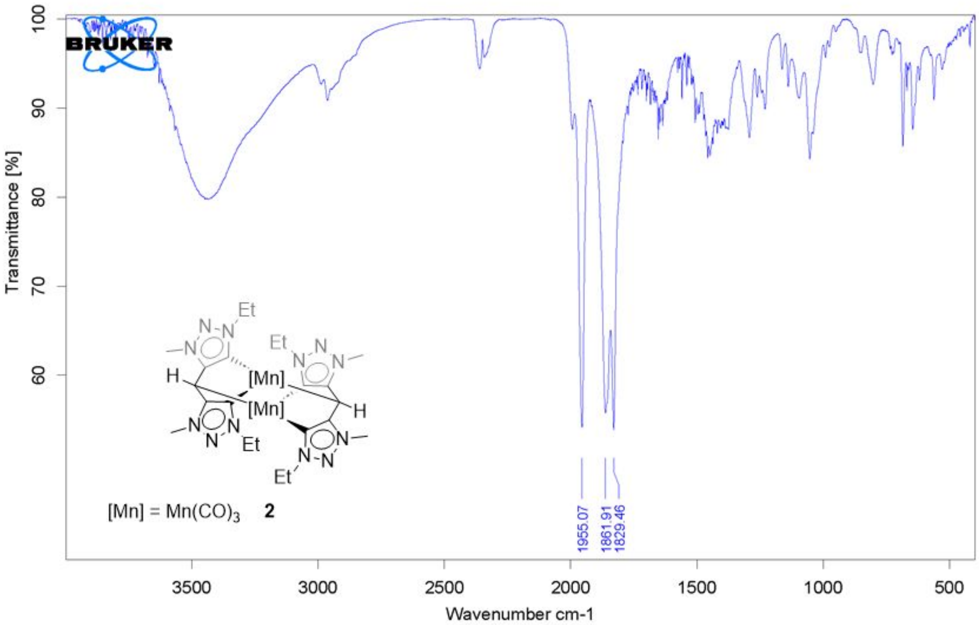

Figure S26. IR spectrum of **2**.

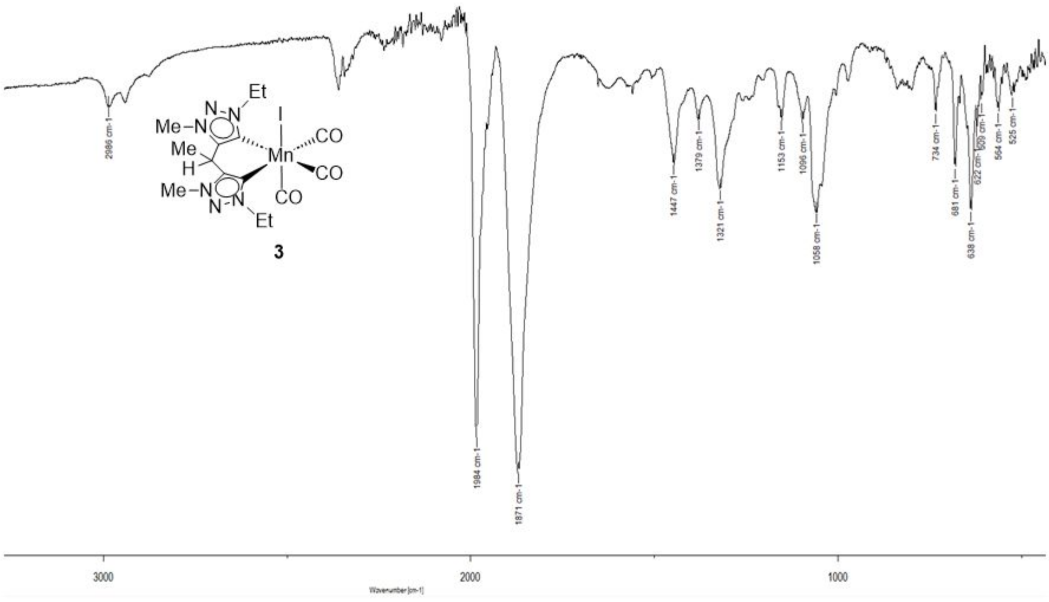

Figure S27. IR spectrum of **3**.

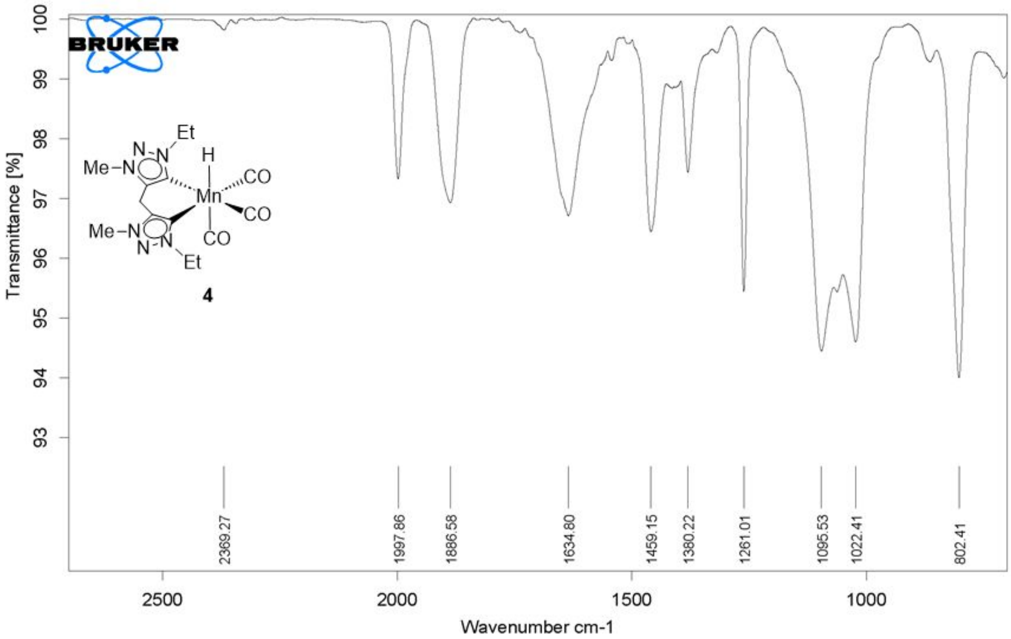

Figure S28. IR spectrum of **4**.

8 Computational coordinates

1

C	1.48029600	0.20407800	-0.14998000
C	1.41724000	1.56656700	0.14958100
C	3.50143000	-1.13210900	-0.92792500
H	2.85822100	-1.98550400	-0.69230000
H	4.42951500	-1.24636500	-0.35139900
C	3.77064000	-1.03614600	-2.41134100
H	4.28494800	-1.94078300	-2.75870700
H	2.83540900	-0.94712900	-2.97947000
H	4.40732600	-0.17266100	-2.64606000
C	3.13279100	3.40982300	0.32476800
H	4.21952600	3.43246300	0.21110500
H	2.68119600	4.11373800	-0.38591000
H	2.86700100	3.70842600	1.34610400
C	0.22604700	2.39527100	0.46810800
H	0.37923300	3.43664100	0.13622500
H	0.07136300	2.41826900	1.56433900
C	-0.97172500	1.80798600	-0.18753200
C	-1.22693000	0.47500300	-0.52147700
C	-3.27355300	-0.48575300	-1.68578200
H	-2.61040000	-1.35437600	-1.77343300
H	-3.54797600	-0.18005200	-2.70557400
C	-4.49511300	-0.77833300	-0.84807200
H	-5.05137900	-1.62127200	-1.27640800
H	-4.21878700	-1.04908000	0.17848800
H	-5.16546300	0.09036800	-0.81040200
C	-2.26785000	3.95988700	-0.42604500
H	-1.52854300	4.53004600	-1.00311800
H	-3.26933000	4.19157600	-0.79725700
H	-2.19683700	4.24337400	0.63105800
C	-1.49911600	-2.17618900	0.34143900

C	0.14477000	-1.71799500	-1.63996500
C	0.99643500	-2.37490100	0.76851800
Mn	-0.06804100	-1.13324900	0.00370300
N	2.81779500	0.05441800	-0.42039000
N	3.55824600	1.15007900	-0.29094100
N	2.67883300	2.06469700	0.06275300
N	-2.04663100	2.54150800	-0.57765300
N	-2.97478400	1.80758200	-1.15665600
N	-2.45681300	0.58455800	-1.11956500
O	-2.39866500	-2.87389500	0.55913600
O	0.29378100	-2.09563300	-2.73423900
O	1.66570200	-3.17567400	1.27030800
Br	-0.36797800	-0.23708200	2.46428200

2

C	1.64297600	-1.00791900	-0.87998000
C	1.31730700	0.18050900	-1.56088700
C	3.90326400	-2.08410300	-0.52009700
H	3.44324400	-3.05348300	-0.75685500
H	3.90244400	-2.00070600	0.57676500
C	5.30348400	-1.99372000	-1.06356500
H	5.90249000	-2.81117100	-0.64451100
H	5.32163000	-2.08328100	-2.15685800
H	5.78601700	-1.04633700	-0.79513000
C	2.69852500	1.75357400	-3.00156000
H	1.74427700	2.23712700	-3.22013500
H	3.38378100	2.50499500	-2.59358200
H	3.11982200	1.35498500	-3.93177800
C	0.02433300	0.81369700	-1.72639900
C	-1.05067600	-0.15640600	-1.77273000
C	-1.12382300	-1.43287900	-1.17887200
C	-2.97482000	-3.17689800	-1.42473500

H	-2.18566900	-3.88294100	-1.14652900
H	-3.36399600	-3.50050200	-2.39929300
C	-4.07675600	-3.10886100	-0.39685700
H	-4.51559700	-4.10201700	-0.24026900
H	-3.69796300	-2.75072800	0.56747100
H	-4.87535800	-2.42759000	-0.71982100
C	-2.60742000	1.10512200	-3.33308400
H	-1.72333600	1.65914700	-3.66211500
H	-3.12557400	0.70336100	-4.20900600
H	-3.27652800	1.79475000	-2.80576600
C	0.60996600	-3.47188500	-1.35305500
C	-0.84278900	-3.43956300	0.81152300
C	1.54538000	-3.18250200	1.02078400
N	3.00109100	-1.05852800	-1.03985300
N	3.54026100	-0.06836800	-1.73677100
N	2.48948700	0.67513100	-2.06134400
N	-2.20374700	0.00814700	-2.48542000
N	-3.00973000	-1.04239700	-2.40809600
N	-2.33253300	-1.88345500	-1.63816700
O	0.79155300	-4.18722200	-2.25505200
O	-1.52638600	-4.20569100	1.36872700
O	2.24865000	-3.84525900	1.67835100
Mn	0.32986000	-2.36758900	0.00144200
H	-0.00383900	1.49617900	-2.58233900
C	-1.64297900	1.00792900	0.87998300
C	-1.31731900	-0.18050400	1.56088600
C	-3.90326200	2.08411900	0.52008700
H	-3.44323100	3.05349500	0.75684100
H	-3.90244800	2.00072200	-0.57677600
C	-5.30348000	1.99375000	1.06356100
H	-5.90248100	2.81120500	0.64450700
H	-5.32162100	2.08331400	2.15685300

H	-5.78602300	1.04637100	0.79512900
C	-2.69855300	-1.75357400	3.00153700
H	-1.74430500	-2.23712100	3.22013000
H	-3.38379700	-2.50499900	2.59354600
H	-3.11986900	-1.35498800	3.93174800
C	-0.02434700	-0.81369400	1.72640800
C	1.05066700	0.15640200	1.77274100
C	1.12382400	1.43287300	1.17887800
C	2.97483900	3.17687300	1.42472500
H	2.18569500	3.88292800	1.14652600
H	3.36403300	3.50047500	2.39927600
C	4.07676200	3.10881600	0.39683300
H	4.51561900	4.10196500	0.24023900
H	3.69795100	2.75068900	-0.56749000
H	4.87535500	2.42753100	0.71978800
C	2.60740200	-1.10512700	3.33310600
H	1.72331900	-1.65918600	3.66208300
H	3.12549500	-0.70336000	4.20906200
H	3.27656200	-1.79472400	2.80581600
C	-0.60995100	3.47188900	1.35306600
C	0.84279600	3.43955900	-0.81152200
C	-1.54536900	3.18251600	-1.02077600
N	-3.00109600	1.05853800	1.03984100
N	-3.54027400	0.06837400	1.73674800
N	-2.48950400	-0.67512800	2.06132700
N	2.20373600	-0.00815600	2.48543300
N	3.00972700	1.04238200	2.40810400
N	2.33253700	1.88344100	1.63817000
O	-0.79152200	4.18722500	2.25506700
O	1.52639400	4.20568200	-1.36873200
O	-2.24863500	3.84527900	-1.67834200
Mn	-0.32985600	2.36759400	-0.00143400

H	0.00381700	-1.49617800	2.58234700

Int1A

C	-0.49370500	0.88005600	-0.83634300
C	-1.07975100	-0.34611300	-1.25560300
C	-1.44825600	3.23497200	-1.04258300
H	-1.33304700	3.47311500	0.02313400
H	-2.45240200	3.56534600	-1.33756200
C	-0.38453000	3.90531100	-1.87590900
H	-0.44452000	4.99423900	-1.75395700
H	0.62418900	3.59234100	-1.58150800
H	-0.51280600	3.67372100	-2.94185600
C	-3.28180400	-0.93244100	-2.30196900
H	-4.15509000	-0.35965400	-2.62764300
H	-2.86934900	-1.48732000	-3.15739800
H	-3.58889500	-1.65269000	-1.52905600
C	-0.57946500	-1.66219200	-1.17438800
H	-1.16882300	-2.49876900	-1.55104700
H	-0.35977500	-0.89952300	1.06116000
C	0.79100900	-1.82138800	-0.88913700
C	1.71320500	-0.87021800	-0.37958400
C	4.22050100	-1.12040400	-0.02311300
H	4.17137400	-0.02878700	0.05079800
H	4.88849900	-1.35335600	-0.86440200
C	4.70905900	-1.76230100	1.25328600
H	5.69041300	-1.35646400	1.52968900
H	4.02275100	-1.56809900	2.08699500
H	4.80798800	-2.84886900	1.13306100
C	1.02306700	-4.20622000	-1.63440800
H	0.55745700	-4.03733900	-2.61656500
H	1.86349100	-4.89720800	-1.74527400
H	0.27125000	-4.65391700	-0.96858600

C	2.52478000	0.93877300	1.57964000
C	2.26409100	1.71898000	-0.88151700
C	0.77709800	2.59262800	1.03961000
Mn	1.22775000	1.00250900	0.33776000
N	-1.46284100	1.78199500	-1.18833000
N	-2.55827000	1.28676300	-1.74659900
N	-2.30651000	-0.02333300	-1.77538800
N	1.51131100	-2.97044400	-1.09622600
N	2.81026600	-2.83086700	-0.81191400
N	2.88554200	-1.57511200	-0.40138500
O	3.33310800	0.95408700	2.41236200
O	2.95928200	2.14917300	-1.70748000
O	0.58647900	3.63420500	1.52269300
O	-0.12429700	-0.22038700	1.71972500
C	-1.33410900	0.38921400	2.17195100
H	-1.25223900	0.47438600	3.26924200
H	-1.40009500	1.41567600	1.77435500
C	-2.56329800	-0.37675200	1.78757500
C	-2.62087700	-1.76801000	1.95010100
C	-3.67788100	0.28587200	1.26126300
C	-3.77138700	-2.47528100	1.60785000
H	-1.75561100	-2.29952300	2.35752300
C	-4.83268500	-0.41903800	0.92531700
H	-3.63472100	1.36768800	1.10391600
C	-4.88350700	-1.80179300	1.10101700
H	-3.80369300	-3.55861500	1.74640300
H	-5.69404900	0.11510400	0.51676000
H	-5.78844700	-2.35578600	0.83997800

Int1

C	-0.99181500	0.73249900	-0.73921400
C	-1.42487200	-0.49939900	-1.30819800

C	-2.34206300	2.85064800	-0.37112600
H	-1.73253400	3.05044500	0.51712100
H	-3.39290200	2.95205400	-0.06848400
C	-2.00984200	3.78527600	-1.51144400
H	-2.10559700	4.83198800	-1.19513900
H	-0.98088500	3.63056200	-1.86389200
H	-2.68643500	3.62076200	-2.36118300
C	-3.65037300	-1.36173200	-2.06154900
H	-3.32996900	-1.71097400	-3.05383100
H	-3.66615200	-2.22804200	-1.38171100
H	-4.65887400	-0.94490900	-2.13807900
C	-0.70966400	-1.68287300	-1.54736100
H	-1.22342100	-2.56783900	-1.92587700
C	0.59187400	-1.78716000	-1.02742500
C	1.42555300	-0.79221800	-0.44556900
C	3.71577100	-1.07576700	0.61056600
H	4.10891000	-0.19996800	0.08165100
H	4.45661200	-1.87704300	0.48917000
C	3.44334100	-0.77351600	2.06547700
H	4.32320800	-0.30930500	2.53149600
H	2.57647800	-0.09965000	2.16433500
H	3.21893000	-1.69819700	2.61798400
C	0.82137900	-4.25798400	-1.35612300
H	-0.13885300	-4.49232400	-0.87165400
H	0.67165200	-4.29327600	-2.44526700
H	1.56244800	-5.01094600	-1.07224300
C	2.64819700	1.72109500	0.18652900
C	1.19009100	1.50934600	-1.94335700
C	0.63069100	2.94997500	0.17732800
Mn	0.97615400	1.24338300	-0.21720500
N	-2.14995300	1.45483900	-0.73715500
N	-3.23945000	0.85214700	-1.21160900

N	-2.76346400	-0.35147500	-1.56746200
N	1.28313400	-2.96723300	-0.94008100
N	2.48210100	-2.83295100	-0.35314500
N	2.52029400	-1.52523100	-0.09190800
O	3.69536400	2.18894400	0.41865500
O	1.27908100	1.67474400	-3.09844400
O	0.55723800	4.07584100	0.48372100
O	0.62850100	0.86400200	1.84991700
C	-0.63860900	0.66466500	2.28811200
H	-0.69948900	0.83453200	3.39794000
H	-1.39409600	1.38922600	1.88501100
C	-1.21094100	-0.72526300	2.06766100
C	-0.36393300	-1.83903300	2.06653900
C	-2.58563000	-0.93989300	1.90606800
C	-0.87297400	-3.12930300	1.92695600
H	0.71167700	-1.66319000	2.16037400
C	-3.10292100	-2.22743400	1.75922300
H	-3.25913700	-0.07478900	1.87586600
C	-2.24671200	-3.33177700	1.77380800
H	-0.19116500	-3.98614100	1.92749800
H	-4.17934500	-2.37202000	1.62463700
H	-2.64787200	-4.34260000	1.65825500

TSA

C	-0.20597800	-1.19871800	0.65283400
C	-1.01672900	-0.19552200	1.20411400
C	-0.39205900	-3.70616900	0.89286400
H	0.11707300	-3.74677700	-0.07772200
H	-1.29521100	-4.32654600	0.82041900
C	0.51102400	-4.16505200	2.01382200
H	0.82329300	-5.20372700	1.84802700
H	1.41764800	-3.54910100	2.07335300

H	-0.00607900	-4.11610200	2.98151400
C	-3.15714100	-0.21119000	2.51813300
H	-3.72664700	-0.99492800	3.02497600
H	-2.80574800	0.52167300	3.25553800
H	-3.80182800	0.29689300	1.78665400
C	-0.84691300	1.23410500	0.96658200
H	-1.58842100	1.90606500	1.41266200
H	-0.80921800	1.09333100	-0.35281800
C	0.55451800	1.61910000	1.06380500
C	1.60414600	0.92022500	0.45448900
C	4.07146800	1.47489300	0.38369800
H	4.09884400	0.48372700	-0.08489000
H	4.66341700	1.41297300	1.30865700
C	4.59604700	2.54996600	-0.53695400
H	5.63570600	2.33425800	-0.81305400
H	4.00786700	2.60103700	-1.46214500
H	4.56833100	3.53451100	-0.05269100
C	0.42036500	3.77540200	2.35041400
H	-0.28473100	3.34169600	3.07021900
H	1.16530900	4.37322600	2.88257700
H	-0.13479000	4.42187900	1.65769400
C	2.33199000	-0.01291900	-2.00811500
C	2.51088500	-1.65798500	0.00932000
C	0.78000800	-2.06374800	-1.84664400
Mn	1.24119200	-0.70807900	-0.75631700
N	-0.83704300	-2.33035600	1.07723800
N	-1.94035400	-2.14539000	1.79736700
N	-2.03089600	-0.81973100	1.85632700
N	1.09849500	2.72194300	1.64001400
N	2.41632900	2.79518900	1.46744200
N	2.67984700	1.69736000	0.76459800
O	3.04521300	0.43878500	-2.80227900

O	3.34061400	-2.27358500	0.54740200
O	0.52067500	-2.94590600	-2.55563600
O	-0.41018600	0.54837600	-1.47862800
C	-1.51649900	-0.10910300	-2.01728900
H	-1.42999000	-0.14446300	-3.12461600
H	-1.58167900	-1.17050500	-1.69400800
C	-2.81922800	0.55380400	-1.64102200
C	-2.97824700	1.93980100	-1.77962200
C	-3.88098800	-0.18863000	-1.11306600
C	-4.16438900	2.56301900	-1.40187500
H	-2.14568100	2.52676600	-2.17758500
C	-5.07744800	0.43022500	-0.74433200
H	-3.75966400	-1.26937100	-0.97920300
C	-5.22088900	1.80992600	-0.88344400
H	-4.27184500	3.64482900	-1.51676600
H	-5.89855600	-0.16831100	-0.34027800
H	-6.15410700	2.29855900	-0.59283500

Int1B

C	-0.65762100	0.31436600	0.86960100
C	-0.57511100	1.67498500	0.56782200
C	-2.52713200	-0.90320100	2.09617900
H	-2.59430300	-1.64744800	1.29256600
H	-3.55200600	-0.57680500	2.31434700
C	-1.84224400	-1.45565800	3.32106300
H	-2.39655500	-2.32428300	3.69802800
H	-0.81896800	-1.78403900	3.10309100
H	-1.79682900	-0.70814800	4.12500700
C	-2.07902600	3.66023100	0.97127700
H	-1.37248900	4.30682300	1.50790700
H	-2.11876300	3.96685700	-0.08216000
H	-3.07253600	3.76635100	1.41509600

C	0.44710800	2.41960300	-0.21254900
H	0.52069500	3.46336500	0.14052100
H	0.13845200	2.45418600	-1.27450400
C	1.76223500	1.74384700	-0.11002100
C	2.03510600	0.39356100	0.09536700
C	4.29618600	-0.74417200	0.32909100
H	3.64574800	-1.59225100	0.57111400
H	4.90796600	-0.53960500	1.21980500
C	5.15306900	-1.01346200	-0.88386100
H	5.78477900	-1.89279700	-0.70736000
H	4.53569800	-1.21786400	-1.76765800
H	5.80920400	-0.16174200	-1.10562900
C	3.19844200	3.80071800	-0.39831100
H	2.73426800	4.12857100	-1.33641100
H	2.79093400	4.39310400	0.43093900
H	4.27877300	3.95661900	-0.45403800
C	1.67903000	-2.08606600	-0.99348100
C	1.25967500	-1.73281500	1.60286300
C	-0.60283700	-2.40073900	0.01221800
Mn	0.62830500	-1.09479400	0.07597700
N	-1.85154100	0.26801400	1.54423300
N	-2.48658300	1.42686600	1.67794300
N	-1.68525800	2.27696600	1.07174600
N	2.95408600	2.39424800	-0.18441000
N	3.98108000	1.58281600	-0.03236600
N	3.40472300	0.39836600	0.13888200
O	2.32521900	-2.70838200	-1.72808700
O	1.71691500	-2.13534900	2.59840600
O	-1.29619800	-3.33580100	0.01307700
O	0.11822100	-0.07788500	-1.66938200
C	-0.86544900	-0.58443700	-2.47878900
H	-0.78270000	-0.12833200	-3.49526900

H	-0.78189400	-1.68362700	-2.66036600
C	-2.27419300	-0.30433000	-1.98757200
C	-2.68551400	1.02084200	-1.77946400
C	-3.19030700	-1.32278700	-1.70626900
C	-3.95931300	1.31888800	-1.30435800
H	-1.98021800	1.82661200	-2.01447500
C	-4.46933300	-1.03324000	-1.22312900
H	-2.89380700	-2.36333800	-1.86682400
C	-4.85817800	0.28811500	-1.01574600
H	-4.26534600	2.36030900	-1.16158300
H	-5.16601000	-1.84822900	-1.00781300
H	-5.85806700	0.51846400	-0.63965800

TS1

C	-1.11845800	0.89003300	-0.94143200
C	-1.61907200	-0.39067900	-1.32509500
C	-2.39066000	3.10887800	-0.93313400
H	-1.95641100	3.38890100	0.03305700
H	-3.46886200	3.29894400	-0.86352700
C	-1.77714800	3.87376100	-2.08220100
H	-1.85603200	4.95427300	-1.90737100
H	-0.71477500	3.62695300	-2.20782100
H	-2.29195000	3.64131500	-3.02455000
C	-3.87824100	-1.23436600	-2.02971900
H	-3.57726300	-1.67543200	-2.99032500
H	-3.91392800	-2.03455100	-1.27692900
H	-4.87072600	-0.78745200	-2.13298300
C	-0.97050900	-1.62502500	-1.38361900
H	-1.50692900	-2.52977200	-1.66941600
C	0.34969200	-1.68304400	-0.93200100
C	1.19621200	-0.64454700	-0.44232600
C	3.60693100	-0.87167900	0.38821200

H	3.78778400	0.12826500	-0.02052800
H	4.39171700	-1.52591000	-0.01517200
C	3.57781500	-0.88349200	1.89846900
H	4.48288800	-0.40642400	2.29807500
H	2.68705800	-0.34898200	2.27761800
H	3.54949000	-1.91876800	2.26989500
C	0.62933100	-4.15985200	-1.22421900
H	-0.25862900	-4.42898900	-0.63361600
H	0.36557100	-4.20337600	-2.29030500
H	1.43120600	-4.87506900	-1.02103300
C	2.21814500	1.72872700	0.74104100
C	1.49651800	1.75099800	-1.77147900
C	0.40106100	3.05929300	0.11826600
Mn	0.75873600	1.35049800	-0.25813000
N	-2.24801000	1.66639900	-1.09144300
N	-3.34582600	1.04354100	-1.48625400
N	-2.94917800	-0.21725500	-1.62598600
N	1.07735700	-2.84454400	-0.86650100
N	2.30716500	-2.65177000	-0.39998100
N	2.35020100	-1.34892200	-0.18090800
O	3.13235800	2.16646900	1.31481700
O	1.96525800	1.99978600	-2.81539800
O	0.34362900	4.19488200	0.39486800
O	0.93731000	0.69389500	2.91899500
C	-0.35199600	0.61050000	2.63509700
H	-1.05992000	1.01580500	3.43631200
H	-0.69036800	1.23953300	1.73503600
C	-0.92395500	-0.76898400	2.32110000
C	-0.10108400	-1.89724600	2.36663700
C	-2.27523800	-0.94727800	1.99082300
C	-0.60664800	-3.16829800	2.08488600
H	0.95283100	-1.73302400	2.61231800

C	-2.79067100	-2.21175900	1.71746700
H	-2.93093300	-0.06714000	1.94259900
C	-1.95346600	-3.33347900	1.75894700
H	0.05739900	-4.03952900	2.11410800
H	-3.84971600	-2.33031300	1.46600200
H	-2.35280300	-4.32846000	1.54041100

Int2

C	-1.12535861	0.81396770	-0.93484355
C	-1.62597261	-0.46674430	-1.31850655
C	-2.39756061	3.03281270	-0.92654555
H	-1.96331161	3.31283570	0.03964545
H	-3.47576261	3.22287870	-0.85693855
C	-1.78404861	3.79769570	-2.07561255
H	-1.86293261	4.87820770	-1.90078255
H	-0.72167561	3.55088770	-2.20123255
H	-2.29885061	3.56524970	-3.01796155
C	-3.88514161	-1.31043130	-2.02313055
H	-3.58416361	-1.75149730	-2.98373655
H	-3.92082861	-2.11061630	-1.27034055
H	-4.87762661	-0.86351730	-2.12639455
C	-0.97740961	-1.70109030	-1.37703055
H	-1.51382961	-2.60583730	-1.66282755
C	0.34279139	-1.75910930	-0.92541255
C	1.18931139	-0.72061230	-0.43573755
C	3.60003039	-0.94774430	0.39480045
H	3.78088339	0.05219970	-0.01393955
H	4.38481639	-1.60197530	-0.00858355
C	3.57091439	-0.95955730	1.90505745
H	4.47598739	-0.48248930	2.30466345
H	2.68015739	-0.42504730	2.28420645
H	3.54258939	-1.99483330	2.27648345

C	0.62243039	-4.23591730	-1.21763055
H	-0.26552961	-4.50505430	-0.62702755
H	0.35867039	-4.27944130	-2.28371655
H	1.42430539	-4.95113430	-1.01444455
C	2.21124439	1.65266170	0.74762945
C	1.48961739	1.67493270	-1.76489055
C	0.39416039	2.98322770	0.12485445
Mn	0.75183539	1.27443270	-0.25154155
N	-2.25491061	1.59033370	-1.08485455
N	-3.35272661	0.96747570	-1.47966555
N	-2.95607861	-0.29332030	-1.61939755
N	1.07045639	-2.92060930	-0.85991255
N	2.30026439	-2.72783530	-0.39339255
N	2.34330039	-1.42498730	-0.17431955
O	3.12545739	2.09040370	1.32140545
O	1.95835739	1.92372070	-2.80880955
O	0.33672839	4.11881670	0.40145645
O	0.93040939	0.61782970	2.92558345
C	-0.35889661	0.53443470	2.64168545
H	-1.06682061	0.93973970	3.44290045
H	-0.69726861	1.16346770	1.74162445
C	-0.93085561	-0.84504930	2.32768845
C	-0.10798461	-1.97331130	2.37322545
C	-2.28213861	-1.02334330	1.99741145
C	-0.61354861	-3.24436330	2.09147445
H	0.94593039	-1.80908930	2.61890645
C	-2.79757161	-2.28782430	1.72405545
H	-2.93783361	-0.14320530	1.94918745
C	-1.96036661	-3.40954430	1.76553545
H	0.05049839	-4.11559430	2.12069645
H	-3.85661661	-2.40637830	1.47259045
H	-2.35970361	-4.40452530	1.54699945

Int3

C	-1.50678100	0.32337600	-0.16389300
C	-1.30190000	1.72900400	-0.28242300
C	-3.70463500	-0.92861000	0.06078600
H	-3.45908500	-1.64570700	-0.73138600
H	-4.73391100	-0.59338300	-0.12063700
C	-3.56821300	-1.55272300	1.42835300
H	-4.22412900	-2.42899800	1.51448800
H	-2.53695100	-1.87963100	1.61478000
H	-3.84831900	-0.84039500	2.21714600
C	-2.84280800	3.69871300	-0.39773600
H	-2.36901400	4.25707200	0.42352500
H	-2.47105900	4.11079000	-1.34763200
H	-3.92657900	3.83807200	-0.34731300
C	-0.10162600	2.46486600	-0.34920600
H	-0.12515600	3.55438500	-0.40575400
C	1.10755100	1.78926300	-0.10058100
C	1.33926600	0.39370700	0.04544500
C	3.47761300	-0.79709700	0.70443600
H	2.78589600	-1.63422500	0.84957300
H	3.95490200	-0.59927200	1.67567900
C	4.51505100	-1.09390800	-0.35231200
H	5.08844800	-1.99065000	-0.08342600
H	4.04644400	-1.27562900	-1.32804000
H	5.21980300	-0.25866900	-0.45851200
C	2.57140600	3.81025000	0.09422100
H	2.36440800	4.23204100	-0.90032400
H	1.93461000	4.33273200	0.82431000
H	3.62172600	3.98485900	0.34485600
C	1.24076500	-2.20423900	-0.93797500
C	0.06880300	-1.57738200	1.43450300

C	-1.15316800	-2.43901300	-0.74970500
Mn	-0.02573400	-1.14997000	-0.29801300
N	-2.86821300	0.25240100	-0.10886000
N	-3.54360900	1.40570000	-0.18766400
N	-2.54693000	2.30066900	-0.29439600
N	2.31386500	2.40120600	0.11733700
N	3.30054400	1.53752700	0.42397500
N	2.66024500	0.36447000	0.37921500
O	2.01494100	-2.92889800	-1.43837700
O	0.18937900	-1.86818300	2.56382600
O	-1.79866600	-3.37404500	-1.04974800
H	0.01681900	-0.57618300	-1.80733600

Int4

C	1.03474000	0.92128300	0.88795400
C	0.69776300	-0.36893200	1.36950800
C	3.24405800	2.14402500	1.06301900
H	2.69355400	2.89383700	0.48629900
H	4.11782300	1.83852600	0.46720000
C	3.65806200	2.69665300	2.40690700
H	4.31323500	3.56734200	2.27523700
H	2.78034600	3.01956100	2.98391400
H	4.20083300	1.94825500	2.99884000
C	2.01899800	-2.23122600	2.40471400
H	1.45904100	-2.32028400	3.34790300
H	1.65263400	-2.99550200	1.70466900
H	3.08191800	-2.40381100	2.59829800
C	-0.57149400	-0.97410600	1.43959600
H	-0.68853100	-1.97241300	1.86297900
C	-1.69880900	-0.18889200	1.13754900
C	-1.77229000	1.11986600	0.57697500
C	-3.84384000	2.47608600	0.04221700

H	-3.15099100	3.32439400	0.05825500
H	-4.65462000	2.70278800	0.74912700
C	-4.39130000	2.21081300	-1.34159000
H	-4.87558100	3.10875700	-1.74686500
H	-3.59071600	1.91951600	-2.03496000
H	-5.13483800	1.40219100	-1.31896600
C	-3.40505000	-1.84749600	1.91066900
H	-2.98748500	-2.68121100	1.32613900
H	-3.06326600	-1.94680200	2.95204000
H	-4.49734100	-1.90391200	1.88200600
C	-1.17561600	3.16240600	-1.33649500
C	0.03033500	3.47320000	0.99321900
C	1.19505500	2.48448300	-1.30343100
Mn	-0.18194900	2.19017700	-0.23505000
N	2.36061600	0.99362100	1.19416700
N	2.90746900	-0.09300700	1.74906100
N	1.85802300	-0.92220800	1.83764000
N	-2.98840600	-0.59349900	1.35363500
N	-3.89589800	0.33228500	1.01552500
N	-3.12059600	1.32870300	0.56743300
O	-1.73156300	3.85480500	-2.10486500
O	0.24605200	4.31753000	1.77791000
O	2.07064500	2.59792500	-2.07431700
H	-0.25314900	0.93215200	-1.23541800
C	4.78044800	-2.60802300	-0.89469200
C	3.47488000	-3.08076800	-0.78941900
C	2.36987100	-2.26143600	-1.09733600
C	2.63077700	-0.93777000	-1.51583400
C	3.93661300	-0.47319200	-1.62410100
C	5.02251400	-1.30078600	-1.32002800
H	5.61646500	-3.26850900	-0.64911100
H	3.27466600	-4.10636100	-0.46693900

H	1.80687700	-0.25758400	-1.74759700
H	4.10614300	0.55612900	-1.95341400
H	6.04530600	-0.92750200	-1.41081600
N	1.10713000	-2.84443200	-0.98472200
C	0.03578500	-2.13888000	-1.14518500
H	0.05200800	-1.05188900	-1.35052100
C	-1.30044400	-2.72098300	-1.14199000
C	-1.52413100	-4.09120600	-0.90411800
C	-2.40555700	-1.89765600	-1.43158500
C	-2.81208600	-4.61204200	-0.93846600
H	-0.66222900	-4.72865900	-0.69245200
C	-3.69408000	-2.42306400	-1.46925800
H	-2.23299900	-0.83045600	-1.60949400
C	-3.90424200	-3.78090400	-1.21840600
H	-2.97424500	-5.67620000	-0.74770800
H	-4.54196700	-1.76655200	-1.68323000
H	-4.91551700	-4.19422700	-1.24315900

TS2

C	1.09188100	0.44122200	1.15644100
C	0.63664700	-0.75942000	1.77374600
C	3.44754700	1.38410600	1.09851500
H	3.45932900	1.51736900	0.00971900
H	4.40845700	0.92774200	1.37048000
C	3.23822300	2.69374200	1.81897000
H	4.02052100	3.41156200	1.53922800
H	2.26506700	3.13479300	1.56569000
H	3.27649500	2.55842200	2.90913400
C	1.79746000	-2.66045600	2.91140800
H	1.21643300	-2.65996100	3.84583100
H	1.37694200	-3.42987600	2.24603700
H	2.83859300	-2.91023200	3.13628800

C	-0.66647700	-1.25990300	1.92322100
H	-0.84246700	-2.20958100	2.43052900
C	-1.74006300	-0.49647000	1.44314900
C	-1.70983600	0.71731500	0.70501100
C	-3.64135200	2.21228500	0.02338000
H	-2.82048800	2.90498100	-0.19325100
H	-4.26583400	2.68480700	0.79625500
C	-4.45821500	1.90160300	-1.20782200
H	-4.88302900	2.82471800	-1.62343100
H	-3.84137100	1.43446600	-1.98653700
H	-5.28764100	1.22220500	-0.97080000
C	-3.59433600	-1.87869600	2.40528300
H	-3.22690400	-2.83208700	2.00101800
H	-3.29536800	-1.81316000	3.46309900
H	-4.68638800	-1.86088700	2.33836700
C	-0.94703400	2.08737100	-1.54875500
C	-0.33611600	3.06642300	0.90119000
C	1.37138100	2.33778700	-0.92754300
Mn	-0.03633900	1.63345400	-0.10244100
N	2.43158600	0.39282200	1.42325100
N	2.87661200	-0.67655700	2.08360000
N	1.75154500	-1.37887800	2.27455700
N	-3.06407900	-0.77867400	1.65549400
N	-3.89013000	0.16145400	1.16474300
N	-3.03541600	1.02861700	0.62010500
O	-1.46660000	2.33821200	-2.56971700
O	-0.58234200	4.01193400	1.54817000
O	2.19661900	2.90243800	-1.54255100
H	0.02814600	0.14403000	-0.90949900
C	4.86328300	-2.00151300	-0.97587600
C	3.53576600	-2.36261300	-0.78080100
C	2.47242500	-1.59623200	-1.31507100

C	2.81972300	-0.42736800	-2.03827300
C	4.15155800	-0.06816300	-2.22261300
C	5.18634700	-0.84955400	-1.70144100
H	5.65912000	-2.62091700	-0.55215000
H	3.27814600	-3.25753100	-0.20712000
H	2.03573100	0.21335800	-2.44455400
H	4.38421800	0.84804700	-2.77434800
H	6.22969100	-0.56061700	-1.84973700
N	1.18777500	-2.02265500	-1.06785900
C	0.17817400	-1.40579900	-1.63680100
H	0.31247100	-0.79866700	-2.55197100
C	-1.19744400	-1.90771900	-1.46182100
C	-1.46030700	-3.10844800	-0.78447000
C	-2.27889900	-1.19622500	-2.00346200
C	-2.76148800	-3.58481500	-0.66219800
H	-0.61622100	-3.65433300	-0.35713000
C	-3.58476100	-1.66711100	-1.87255900
H	-2.08262700	-0.25447200	-2.52556200
C	-3.83262700	-2.86435100	-1.20147000
H	-2.94806400	-4.52680800	-0.13819600
H	-4.41461200	-1.09555500	-2.29650000
H	-4.85516700	-3.23719900	-1.09864100

Int5

C	1.06080700	0.42441900	1.17709300
C	0.58075300	-0.77331300	1.78557000
C	3.45911200	1.29312400	1.11977600
H	3.48506400	1.40724700	0.02855500
H	4.40123200	0.80712600	1.40405300
C	3.28732800	2.61234100	1.83270500
H	4.09082300	3.30288300	1.54568300
H	2.32858300	3.08441300	1.58139600

H	3.32552700	2.48285400	2.92337500
C	1.71181300	-2.72254300	2.88901600
H	1.12368000	-2.72338300	3.81754700
H	1.28857900	-3.47184700	2.20499900
H	2.74839100	-2.98575200	3.11712300
C	-0.72659400	-1.24859000	1.93540900
H	-0.92581300	-2.20513100	2.41907800
C	-1.76841800	-0.45251400	1.44927000
C	-1.69045400	0.75681800	0.70405200
C	-3.60198800	2.28698300	-0.01539600
H	-2.77014700	2.96474900	-0.23379500
H	-4.21490500	2.77364200	0.75737200
C	-4.42762900	1.98483100	-1.24277800
H	-4.83455500	2.91490100	-1.65980400
H	-3.82583900	1.50353600	-2.02447900
H	-5.27108300	1.32498900	-1.00084200
C	-3.68193900	-1.82271000	2.32436500
H	-3.39730700	-2.76406700	1.83367100
H	-3.33642400	-1.85106900	3.36758500
H	-4.77092200	-1.72378100	2.30657200
C	-0.89495100	2.10463400	-1.52296800
C	-0.31080200	3.14049200	0.81762500
C	1.42969700	2.34285600	-0.85948800
Mn	-0.00933000	1.65463900	-0.04877000
N	2.40969900	0.33385700	1.44040600
N	2.81223300	-0.75226700	2.07533900
N	1.68628700	-1.42919600	2.26963000
N	-3.10402200	-0.70441200	1.63457500
N	-3.88634000	0.24370000	1.11815800
N	-3.01732800	1.09274000	0.58743900
O	-1.41475400	2.36556400	-2.53835800
O	-0.52344000	4.12802100	1.40961400

O	2.25654900	2.92286200	-1.44984200
H	0.05372300	-0.33800600	-1.02550200
C	4.87889700	-1.95498900	-1.05073900
C	3.57935300	-2.36206700	-0.80149700
C	2.44162300	-1.64216000	-1.29556800
C	2.74587100	-0.45344900	-2.04136800
C	4.05921200	-0.05530300	-2.27561100
C	5.14552700	-0.79258900	-1.79516300
H	5.71063600	-2.54783200	-0.65482200
H	3.38323000	-3.26587300	-0.21497700
H	1.93268200	0.15831000	-2.43811300
H	4.23854700	0.86424300	-2.84443100
H	6.17249000	-0.47205200	-1.98724800
N	1.21247900	-2.11233200	-1.03157200
C	0.13687700	-1.37399700	-1.53398600
H	0.24516400	-1.05511200	-2.60102500
C	-1.20987100	-2.02776600	-1.38973200
C	-1.35131500	-3.34272000	-0.93287500
C	-2.36855400	-1.31403000	-1.72778400
C	-2.61340300	-3.92484600	-0.80952100
H	-0.44242200	-3.88804200	-0.66694100
C	-3.63274000	-1.88675100	-1.59668100
H	-2.26579500	-0.28544700	-2.08973600
C	-3.76162600	-3.19842700	-1.13406600
H	-2.70420200	-4.95544600	-0.45330900
H	-4.52318800	-1.30641400	-1.85563600
H	-4.75099900	-3.65220700	-1.03065500

TS3

C	1.26672400	0.17337400	1.57995000
C	0.57458400	-0.93660300	2.15389600
C	3.74690800	0.75195300	1.82945800

H	3.88544200	0.90457000	0.75276500
H	4.58603000	0.13274500	2.17128700
C	3.68137900	2.05091600	2.59631000
H	4.58298400	2.64713800	2.40590400
H	2.81044900	2.65029700	2.30017800
H	3.61578500	1.86987400	3.67816400
C	1.31095300	-2.93350800	3.49351500
H	0.63794300	-2.78603000	4.34961600
H	0.85989600	-3.67568500	2.82000500
H	2.27377000	-3.30773700	3.85135500
C	-0.78271200	-1.26056100	2.13432000
H	-1.16118500	-2.15140500	2.63533500
C	-1.63365900	-0.40765400	1.42883400
C	-1.31249400	0.74013300	0.64841000
C	-2.94108600	2.32611100	-0.52791500
H	-2.11251600	3.03921400	-0.46471800
H	-3.80536300	2.79644400	-0.03964800
C	-3.26616700	1.94386800	-1.95258100
H	-3.46297000	2.84151800	-2.55247600
H	-2.43344600	1.40209000	-2.42151000
H	-4.15798900	1.30379200	-1.99339000
C	-3.79242500	-1.51611000	2.08085000
H	-3.48550800	-2.52337700	1.76940900
H	-3.66652400	-1.42572900	3.16926700
H	-4.84420000	-1.36532800	1.82214200
C	0.00637400	2.27692200	-1.27761300
C	0.19406200	2.95507400	1.20191200
C	2.13395500	2.15802200	-0.24178000
Mn	0.52587400	1.53508900	0.26426300
N	2.55341900	-0.06694300	2.01357700
N	2.73773800	-1.16477500	2.72248100
N	1.52352300	-1.69622800	2.79627300

N	-3.00165100	-0.52837800	1.40078600
N	-3.58612000	0.44052100	0.70888300
N	-2.57070300	1.17363600	0.28657300
O	-0.21898800	2.79947600	-2.29753800
O	-0.04348200	3.89194400	1.86275100
O	3.07605500	2.72367200	-0.63319400
H	-0.84581300	-0.33984700	-1.90948400
C	4.22855200	-0.46394500	-1.66909200
C	3.09580700	-1.08240700	-1.17211600
C	1.79569400	-0.92312400	-1.77229200
C	1.77002000	-0.02551000	-2.90228600
C	2.92305400	0.58966100	-3.38297000
C	4.17309500	0.38222500	-2.79280100
H	5.18928100	-0.63605500	-1.16833800
H	3.16578600	-1.73862000	-0.29643500
H	0.81801800	0.19134500	-3.39367400
H	2.84042100	1.26025100	-4.24754300
H	5.07094900	0.87308900	-3.17666500
N	0.75537800	-1.59469700	-1.27553900
C	-0.48166200	-1.39997700	-1.94270800
H	-0.41821500	-1.59292800	-3.04844100
C	-1.59729600	-2.27541200	-1.43082100
C	-1.33571000	-3.41889200	-0.66673000
C	-2.93091300	-1.99337800	-1.75791500
C	-2.36916900	-4.26390200	-0.25968000
H	-0.29448300	-3.61437500	-0.40019700
C	-3.96971700	-2.82925300	-1.34916900
H	-3.15480400	-1.09630900	-2.34577000
C	-3.69219400	-3.97647800	-0.60176900
H	-2.14162100	-5.15556200	0.33276400
H	-5.00242200	-2.58425100	-1.61392500
H	-4.50332700	-4.63564700	-0.28053600

Int6

C	-1.09442900	1.60185800	0.20118800
C	-0.11071300	2.39941000	-0.45056800
C	-3.60454500	1.97517200	0.32052700
H	-3.82198000	0.93167600	0.05896200
H	-4.25608000	2.60108500	-0.30270900
C	-3.83246800	2.24767100	1.78766400
H	-4.86844900	2.00904600	2.06123300
H	-3.16932000	1.64179500	2.41848400
H	-3.65333100	3.30544900	2.02558100
C	-0.23063700	4.45174000	-1.88325900
H	0.45940800	5.06494000	-1.28446200
H	0.33214800	4.03478400	-2.73125700
H	-1.03635100	5.08583600	-2.26401100
C	1.28856900	2.28198300	-0.45932300
H	1.90322900	3.00410300	-0.99812600
C	1.88318100	1.29579400	0.34277900
C	1.27388800	0.27401600	1.12122900
C	2.34716400	-1.25469900	2.84580400
H	1.32461900	-1.30531600	3.23534100
H	2.97566500	-0.85915800	3.65610800
C	2.84897000	-2.60582300	2.39851500
H	2.83392000	-3.31012900	3.24047500
H	2.22121000	-3.02634800	1.60114800
H	3.88138500	-2.54175300	2.02866700
C	4.25318500	2.06158400	0.06883900
H	4.19520100	2.10475600	-1.02697600
H	4.14497000	3.08317800	0.46575400
H	5.23086400	1.66440200	0.35813500
C	-0.47653300	-1.89186300	1.73057300
C	-0.78371000	0.50409100	2.76027400

C	-2.46729400	-0.70518800	1.22245800
Mn	-0.74391300	-0.22101400	1.16367000
N	-2.24239200	2.27317800	-0.10495400
N	-2.12224200	3.34868000	-0.88332000
N	-0.79736700	3.40266400	-1.08856700
N	3.22916100	1.20105700	0.58509700
N	3.53691400	0.26258400	1.49266400
N	2.34418400	-0.24702300	1.79049300
O	-0.42935300	-3.00989900	2.06705700
O	-0.76372800	0.98459600	3.82705000
O	-3.54160200	-1.12949300	1.40222600
H	1.01426700	-2.54354600	-0.11613100
C	-3.83812600	-1.13133100	-2.32776400
C	-2.61187300	-0.71495500	-1.83191000
C	-1.60022800	-1.63468400	-1.42584800
C	-1.92753500	-3.01263000	-1.58660800
C	-3.16344400	-3.42068000	-2.08733100
C	-4.13992600	-2.49466200	-2.46122300
H	-4.57444500	-0.37837700	-2.63014100
H	-2.37796900	0.35002200	-1.75427500
H	-1.21320400	-3.77608600	-1.26600100
H	-3.37302900	-4.49274300	-2.17086200
H	-5.10636800	-2.82225700	-2.85175100
N	-0.42630900	-1.15059200	-0.91495900
C	0.72331800	-2.00707000	-1.04532400
H	0.51860100	-2.82566300	-1.76748100
C	1.95754700	-1.31459600	-1.57677900
C	1.85642300	-0.32640300	-2.56497900
C	3.23746800	-1.71321900	-1.17149400
C	2.99653400	0.24090600	-3.12992100
H	0.85915600	-0.00418800	-2.87744200
C	4.38350800	-1.14531000	-1.72867800

H	3.33388600	-2.48166900	-0.39800900
C	4.26749300	-0.16429600	-2.71411000
H	2.89514300	1.01231100	-3.89946700
H	5.37176000	-1.46503800	-1.38543200
H	5.16202100	0.28788400	-3.15135700

Int7

C	0.83980000	-0.84570000	0.27840000
C	1.21620000	0.23110000	1.10110000
C	1.70610000	-3.24510000	0.33300000
H	0.68720000	-3.65300000	0.36490000
H	2.28010000	-3.76660000	1.10950000
C	2.34350000	-3.42150000	-1.02260000
H	2.32670000	-4.48070000	-1.30970000
H	1.81250000	-2.85450000	-1.79820000
H	3.38860000	-3.08550000	-1.01310000
C	2.82520000	0.49850000	3.00430000
H	3.13840000	1.46560000	2.58840000
H	2.15170000	0.66550000	3.85620000
H	3.70130000	-0.06510000	3.33740000
C	0.87520000	1.64240000	1.07750000
H	0.55730000	2.04430000	2.05410000
C	-0.00700000	2.00180000	-0.01690000
C	-0.63000000	1.22290000	-1.00720000
C	-1.83010000	2.02400000	-3.11470000
H	-1.58730000	1.01750000	-3.47260000
H	-1.36290000	2.73080000	-3.81430000
C	-3.31940000	2.25640000	-3.03720000
H	-3.77100000	2.12820000	-4.02920000
H	-3.80670000	1.54640000	-2.35520000
H	-3.54340000	3.27540000	-2.69330000
C	0.24870000	4.46680000	0.36210000

H	-0.23040000	4.54690000	1.34690000
H	1.33610000	4.35820000	0.48740000
H	0.01560000	5.35600000	-0.23000000
C	-2.19720000	-0.89410000	-2.11060000
C	0.40770000	-0.71650000	-2.46900000
C	-0.82910000	-2.63200000	-1.24640000
Mn	-0.71340000	-0.84750000	-1.11750000
N	1.63810000	-1.85170000	0.76110000
N	2.42630000	-1.52880000	1.78340000
N	2.15860000	-0.24650000	1.96550000
N	-0.24110000	3.30460000	-0.34010000
N	-0.94930000	3.44440000	-1.44850000
N	-1.14540000	2.18720000	-1.83560000
O	-3.17650000	-1.04980000	-2.72620000
O	1.14460000	-0.57820000	-3.36590000
O	-0.98320000	-3.76380000	-1.48650000
H	-3.45710000	0.49370000	-0.53910000
C	-1.83830000	-4.09350000	2.16870000
C	-1.54990000	-2.80790000	1.73410000
C	-2.50610000	-1.99910000	1.05560000
C	-3.79090000	-2.58760000	0.88170000
C	-4.07300000	-3.87940000	1.32380000
C	-3.10610000	-4.65610000	1.96640000
H	-1.06380000	-4.66710000	2.68940000
H	-0.56730000	-2.37020000	1.92650000
H	-4.56670000	-2.03590000	0.34340000
H	-5.07020000	-4.29480000	1.14370000
H	-3.33380000	-5.66870000	2.30740000
N	-2.13490000	-0.75460000	0.61050000
C	-3.18550000	0.22510000	0.50290000
H	-4.13340000	-0.15810000	0.93350000
C	-2.90550000	1.51300000	1.24430000

C	-2.23700000	1.50030000	2.47520000
C	-3.37880000	2.73970000	0.76100000
C	-2.05740000	2.67420000	3.20480000
H	-1.84640000	0.54720000	2.84450000
C	-3.19560000	3.91950000	1.48270000
H	-3.89340000	2.76740000	-0.20540000
C	-2.53640000	3.89100000	2.71270000
H	-1.53430000	2.64330000	4.16520000
H	-3.56270000	4.86750000	1.07920000
H	-2.38870000	4.81310000	3.28100000
C	3.45180000	2.19450000	-0.61460000
H	3.92460000	2.86660000	-1.37190000
H	2.55130000	1.79340000	-1.15250000
C	4.39120000	1.02040000	-0.41270000
C	4.43740000	-0.04690000	-1.31950000
C	5.24630000	0.98690000	0.69460000
C	5.31440000	-1.11450000	-1.12560000
H	3.76270000	-0.04610000	-2.18310000
C	6.11830000	-0.08250000	0.89860000
H	5.20070000	1.81860000	1.40290000
C	6.15500000	-1.14100000	-0.01070000
H	5.33730000	-1.93740000	-1.84550000
H	6.77420000	-0.09260000	1.77410000
H	6.83440000	-1.98240000	0.14750000
O	3.11980000	2.86920000	0.52850000
H	2.01790000	2.29720000	0.87230000

TS4

C	0.82878100	-0.87031900	0.25376700
C	1.20514800	0.20649800	1.07654200
C	1.69505300	-3.26963400	0.30840200
H	0.67613000	-3.67756000	0.34035500

H	2.26905100	-3.79120400	1.08488700
C	2.33247000	-3.44611000	-1.04717300
H	2.31562300	-4.50533000	-1.33427000
H	1.80148200	-2.87908800	-1.82277000
H	3.37752600	-3.11010900	-1.03766000
C	2.81416300	0.47395800	2.97967400
H	3.12732000	1.44103000	2.56378800
H	2.14063500	0.64088000	3.83164600
H	3.69024200	-0.08971800	3.31282100
C	0.86415600	1.61781500	1.05287200
H	0.54621600	2.01969600	2.02951600
C	-0.01807800	1.97721600	-0.04144100
C	-0.64108500	1.19836800	-1.03179000
C	-1.84119800	1.99944800	-3.13929300
H	-1.59836000	0.99295800	-3.49714300
H	-1.37395400	2.70618000	-3.83884000
C	-3.33042300	2.23184300	-3.06176700
H	-3.78209500	2.10358800	-4.05375100
H	-3.81774600	1.52185400	-2.37981300
H	-3.55444500	3.25079200	-2.71791100
C	0.23763400	4.44217600	0.33747000
H	-0.24150400	4.52236900	1.32228000
H	1.32504400	4.33358100	0.46284200
H	0.00453100	5.33144400	-0.25454200
C	-2.20825700	-0.91865400	-2.13514700
C	0.39663500	-0.74107200	-2.49357000
C	-0.84016200	-2.65653600	-1.27100100
Mn	-0.72445900	-0.87203500	-1.14212400
N	1.62700700	-1.87624200	0.73652600
N	2.41520100	-1.55341000	1.75880000
N	2.14752700	-0.27105100	1.94088500
N	-0.25216400	3.28003100	-0.36469800

N	-0.96035300	3.41979400	-1.47310500
N	-1.15641000	2.16258000	-1.86019800
O	-3.18755900	-1.07437100	-2.75081500
O	1.13358000	-0.60275300	-3.39045900
O	-0.99429200	-3.78840700	-1.51113400
H	-3.46815100	0.46911600	-0.56365600
C	-1.84938600	-4.11811100	2.14410900
C	-1.56095000	-2.83244200	1.70954600
C	-2.51716300	-2.02368500	1.03106000
C	-3.80195300	-2.61219000	0.85710900
C	-4.08401000	-3.90395800	1.29918600
C	-3.11711200	-4.68066100	1.94180900
H	-1.07485500	-4.69164700	2.66476800
H	-0.57838200	-2.39480700	1.90192700
H	-4.57773100	-2.06051800	0.31881800
H	-5.08127400	-4.31941700	1.11914600
H	-3.34483400	-5.69330700	2.28284200
N	-2.14596700	-0.77917700	0.58594300
C	-3.19651700	0.20053900	0.47829500
H	-4.14449100	-0.18264200	0.90891200
C	-2.91659800	1.48846500	1.21973200
C	-2.24805100	1.47567800	2.45065600
C	-3.38990300	2.71507000	0.73638300
C	-2.06849400	2.64964900	3.18018100
H	-1.85749800	0.52262400	2.81992700
C	-3.20662700	3.89494300	1.45813700
H	-3.90447500	2.74280000	-0.22997500
C	-2.54750300	3.86637800	2.68811100
H	-1.54537500	2.61867000	4.14057300
H	-3.57374900	4.84296500	1.05458000
H	-2.39972700	4.78855200	3.25641600
C	3.44073300	2.16989400	-0.63921000

H	3.91353700	2.84204000	-1.39649000
H	2.54026800	1.76883200	-1.17712500
C	4.38013200	0.99582700	-0.43729000
C	4.42630300	-0.07151600	-1.34404000
C	5.23519600	0.96230400	0.67003800
C	5.30332400	-1.13904100	-1.15018200
H	3.75164600	-0.07064500	-2.20773000
C	6.10723300	-0.10710100	0.87401700
H	5.18966700	1.79397700	1.37835400
C	6.14396500	-1.16556500	-0.03526900
H	5.32624200	-1.96201400	-1.87012100
H	6.76310300	-0.11722500	1.74948800
H	6.82329800	-2.00699900	0.12287200
O	3.10875700	2.84466500	0.50395100
H	2.00686200	2.27266500	0.84774000

Int8

C	0.80803300	-1.10429900	0.02476600
C	1.10995600	-0.12642500	0.97271900
C	1.88606500	-3.41404600	-0.22642300
H	0.91158400	-3.92120200	-0.22410300
H	2.52665200	-3.96077600	0.47662200
C	2.49494100	-3.38756900	-1.60558600
H	2.67611700	-4.41418400	-1.94809000
H	1.83770900	-2.90442800	-2.33763100
H	3.45539000	-2.85495200	-1.60253800
C	2.73132000	0.05641400	2.88387400
H	3.00510200	1.07844500	2.56180800
H	2.03111200	0.09383700	3.72886500
H	3.62100100	-0.51135200	3.16944100
C	0.56689000	1.22892800	1.18918900
H	-0.12644300	1.24264400	2.05012100

C	-0.09184100	1.73237000	-0.03026900
C	-0.58449800	1.05667400	-1.14695500
C	-1.47170100	2.05605500	-3.32134600
H	-1.24536500	1.05828700	-3.71366400
H	-0.89378900	2.77354100	-3.91951700
C	-2.94744000	2.36853400	-3.37938000
H	-3.29917200	2.32751100	-4.41783200
H	-3.53973800	1.64714900	-2.80031000
H	-3.15355800	3.37520900	-2.99149200
C	0.24464800	4.14771400	0.57125400
H	-0.39295800	4.20606300	1.46301700
H	1.29179100	3.94389800	0.86976000
H	0.16672000	5.07967600	0.00518100
C	-2.13515800	-0.92644600	-2.49806000
C	0.44073400	-0.82646400	-2.69075700
C	-0.88047500	-2.77630800	-1.58201400
Mn	-0.73629200	-0.99965300	-1.38649600
N	1.70365800	-2.09099000	0.36455300
N	2.48406600	-1.81772200	1.40643300
N	2.10655900	-0.61176600	1.76120400
N	-0.20447400	3.06300900	-0.27793400
N	-0.72274600	3.31373400	-1.45986300
N	-0.93083100	2.10106700	-1.96603600
O	-3.03281400	-0.97368200	-3.24014100
O	1.20799400	-0.64420900	-3.55100300
O	-1.01686900	-3.90447700	-1.84411800
H	-3.14414800	0.53959000	-0.94069000
C	-1.74042300	-3.82711400	2.43739900
C	-1.51923600	-2.86466700	1.46333700
C	-2.40859100	-1.76854000	1.24131000
C	-3.54141100	-1.74045700	2.12170400
C	-3.75056100	-2.71582000	3.09239600

C	-2.86302200	-3.77937100	3.27330000
H	-1.01316600	-4.63912100	2.54538400
H	-0.63371900	-2.93646600	0.83714000
H	-4.26005200	-0.92115300	2.05966200
H	-4.63651600	-2.63314200	3.73120300
H	-3.03472100	-4.53885300	4.03923100
N	-2.16752500	-0.84212700	0.27638000
C	-3.18643300	0.16543700	0.09233400
H	-4.20859000	-0.26712700	0.17251200
C	-3.13466600	1.38973000	0.98033700
C	-2.61248500	1.36374100	2.28009900
C	-3.63054600	2.61017500	0.49721800
C	-2.59352100	2.51452700	3.07021000
H	-2.20109000	0.42903800	2.66855200
C	-3.61025900	3.76394100	1.27956900
H	-4.03125600	2.65442900	-0.52113800
C	-3.09320000	3.72000700	2.57564100
H	-2.17859100	2.46908700	4.08092500
H	-3.99321400	4.70382000	0.87288400
H	-3.07249500	4.62150600	3.19304600
C	3.34177900	2.31655200	0.02891200
H	3.74113900	3.08533300	-0.70256800
H	2.43472000	1.93121500	-0.53682800
C	4.34034300	1.16647300	-0.03960800
C	4.35825300	0.26924500	-1.11670500
C	5.27861800	0.98789200	0.98259000
C	5.29107400	-0.76607000	-1.17612000
H	3.61606300	0.38129600	-1.91712500
C	6.20451900	-0.05510700	0.93842200
H	5.24336000	1.68460100	1.82560600
C	6.21617200	-0.93838700	-0.14282300
H	5.29440600	-1.45017000	-2.02996300

H	6.92505700	-0.18201600	1.75220000
H	6.93942800	-1.75715100	-0.18087400
O	3.08241400	2.80325300	1.25180700
H	1.41994100	1.91157300	1.44433900

Int9

C	-2.02050000	-0.28780000	-0.07860000
C	-2.00940000	0.07230000	1.26720000
C	-3.76380000	0.18920000	-1.84160000
H	-3.34690000	-0.71260000	-2.30820000
H	-3.33910000	1.04740000	-2.38800000
C	-5.26950000	0.20120000	-1.88540000
H	-5.60460000	0.12800000	-2.92700000
H	-5.69450000	-0.64740000	-1.33310000
H	-5.68410000	1.12290000	-1.46020000
C	-3.48780000	1.53040000	2.69430000
H	-4.49910000	1.92550000	2.56880000
H	-3.45850000	0.87590000	3.57370000
H	-2.78400000	2.36390000	2.82870000
C	-0.98030000	-0.19990000	2.30000000
H	-1.45490000	-0.35050000	3.28580000
H	-0.31740000	0.68690000	2.41870000
C	-0.19790000	-1.40100000	1.91740000
C	0.05150000	-1.92450000	0.64830000
C	1.37200000	-4.00330000	0.02680000
H	0.90770000	-3.80020000	-0.94550000
H	1.03760000	-5.00080000	0.34600000
C	2.87840000	-3.91770000	-0.02950000
H	3.26770000	-4.63860000	-0.75890000
H	3.21330000	-2.92010000	-0.34150000
H	3.32470000	-4.14570000	0.94730000
C	0.48280000	-2.06540000	4.25400000

H	-0.51310000	-2.20370000	4.69450000
H	1.15900000	-2.82850000	4.64770000
H	0.85990000	-1.07060000	4.52130000
C	1.18030000	-1.42330000	-1.88860000
C	-1.25190000	-2.35970000	-1.76150000
C	-0.71260000	0.03020000	-2.58570000
Mn	-0.37870000	-0.95910000	-1.11870000
N	-3.22570000	0.22080000	-0.48400000
N	-3.90130000	0.89430000	0.43750000
N	-3.13720000	0.79220000	1.50440000
N	0.42840000	-2.20440000	2.81810000
N	1.04430000	-3.22140000	2.24690000
N	0.79340000	-3.03440000	0.95460000
O	2.17250000	-1.71420000	-2.41550000
O	-1.83630000	-3.28280000	-2.16430000
O	-0.89210000	0.61520000	-3.57190000
H	2.17870000	-0.68240000	0.27300000
C	-1.59420000	3.62560000	-0.06190000
C	-0.97790000	2.44950000	-0.45840000
C	0.15940000	1.91170000	0.21220000
C	0.68330000	2.73740000	1.25740000
C	0.05300000	3.91870000	1.64560000
C	-1.10710000	4.37270000	1.01850000
H	-2.46740000	3.97740000	-0.62140000
H	-1.36390000	1.91500000	-1.32520000
H	1.60860000	2.46090000	1.76530000
H	0.49700000	4.50500000	2.45640000
H	-1.58670000	5.30520000	1.32330000
N	0.65950000	0.68230000	-0.10340000
C	1.94530000	0.37840000	0.46760000
H	1.95480000	0.44610000	1.58480000
C	3.10840000	1.19220000	-0.05800000

C	3.05100000	1.80040000	-1.31670000
C	4.28690000	1.31120000	0.68800000
C	4.14650000	2.50210000	-1.81680000
H	2.12800000	1.72120000	-1.89800000
C	5.38460000	2.01080000	0.19040000
H	4.34060000	0.84940000	1.68040000
C	5.31770000	2.60980000	-1.06760000
H	4.08320000	2.97230000	-2.80140000
H	6.29490000	2.09500000	0.78990000
H	6.17430000	3.16350000	-1.45960000

TS5

C	-2.08566600	-0.15581200	-0.36068200
C	-2.37549700	-0.17566700	1.00959900
C	-3.57220900	0.48785000	-2.30535500
H	-2.61422700	0.51346700	-2.83857500
H	-4.03984900	1.47569600	-2.41840200
C	-4.46696200	-0.61233100	-2.82454800
H	-4.67874900	-0.45441400	-3.88906600
H	-3.99116400	-1.59583800	-2.71672000
H	-5.42403600	-0.63098200	-2.28723400
C	-4.35413700	0.55503700	2.37842800
H	-3.95298700	1.44508300	2.88073500
H	-5.40910800	0.71544800	2.14308600
H	-4.25410200	-0.30770600	3.04659600
C	-1.41738100	-0.54246700	2.03959000
H	-1.75120700	-0.48048500	3.08014500
H	-0.43970100	0.31169500	1.58986500
C	-0.69230400	-1.74166400	1.64528700
C	-0.12664900	-1.94508300	0.37792700
C	1.32517800	-3.85551000	-0.43490300
H	1.09919000	-3.40912600	-1.41051800

H	0.97605900	-4.89674900	-0.46569700
C	2.79560500	-3.77555500	-0.09941200
H	3.38850900	-4.26677800	-0.88074700
H	3.13356300	-2.73298200	-0.02837700
H	3.01065000	-4.27369800	0.85487500
C	-0.64733700	-3.03470400	3.80270800
H	-1.71377900	-2.84980700	3.97453100
H	-0.41249500	-4.07400900	4.04485200
H	-0.06091100	-2.36912300	4.44853300
C	1.51140300	-0.83120300	-1.59319500
C	-0.78809600	-1.51097200	-2.30382600
C	-0.20055800	1.01202100	-2.08867300
Mn	-0.16379000	-0.45436400	-1.05013600
N	-3.24829700	0.31427300	-0.89337000
N	-4.19967200	0.61427700	-0.01353400
N	-3.63853100	0.30205600	1.15151800
N	-0.33363900	-2.80196700	2.41313900
N	0.40969900	-3.68228900	1.75018400
N	0.50267800	-3.14346700	0.53845800
O	2.52039000	-1.15336300	-2.07498500
O	-1.22134900	-2.21702800	-3.12395200
O	-0.19207300	1.94872100	-2.77663800
H	1.71714500	-0.63779200	1.42473000
C	-1.29083300	4.16528600	0.54025900
C	-0.97862100	2.81857800	0.38952800
C	0.22757200	2.27102000	0.88579700
C	1.10288400	3.17435300	1.53737200
C	0.78191000	4.52250200	1.68352000
C	-0.41735100	5.03779800	1.19316800
H	-2.23471400	4.53738700	0.13196800
H	-1.67052500	2.17169700	-0.14660200
H	2.06257900	2.83092600	1.92473100

H	1.49387200	5.18116000	2.18901200
H	-0.66270000	6.09560600	1.30898200
N	0.47519100	0.89517500	0.78108000
C	1.75272800	0.45886100	1.31860600
H	1.88969500	0.82287300	2.35881400
C	2.99361100	0.80111800	0.52925900
C	2.98644200	1.67081300	-0.56717700
C	4.20893500	0.20618100	0.89633400
C	4.15784000	1.93384300	-1.27787600
H	2.04796600	2.13169000	-0.88340000
C	5.37977500	0.46147600	0.18588200
H	4.23016200	-0.48142800	1.74840600
C	5.35851000	1.32959500	-0.90774500
H	4.12768400	2.61265400	-2.13391200
H	6.31333600	-0.02202300	0.48453900
H	6.27409600	1.53146500	-1.46871300

Int10

C	1.95741300	-0.83846500	0.14570200
C	2.10018700	-1.22732000	-1.21538400
C	3.72393200	0.12233200	1.71292300
H	3.13490700	-0.37251100	2.49452000
H	3.46089200	1.19072900	1.75046900
C	5.20051800	-0.08513200	1.92460200
H	5.47467300	0.28222200	2.92082200
H	5.46972400	-1.14802800	1.86921200
H	5.80347900	0.45736800	1.18725400
C	4.02601700	-1.18213300	-2.83991500
H	3.52646900	-0.59310500	-3.62019600
H	5.07490300	-0.88262400	-2.77529800
H	3.96556500	-2.24542300	-3.10648500
C	1.13996200	-1.77732000	-2.07471000

H	1.37121600	-2.00455300	-3.11494500
H	0.33998200	0.50206500	-1.46845900
C	-0.08930600	-2.13339600	-1.50790000
C	-0.57353000	-1.94544600	-0.17577500
C	-2.79190500	-2.79468400	0.79873600
H	-2.24936900	-2.76404400	1.74881400
H	-3.15993300	-3.82158500	0.67494800
C	-3.92346800	-1.79795300	0.74165600
H	-4.61340900	-1.96246800	1.57913100
H	-3.55657900	-0.76532500	0.80603900
H	-4.49080600	-1.90101000	-0.19330000
C	-1.06489800	-3.18752900	-3.57952100
H	-0.23814100	-3.88734600	-3.75970100
H	-2.01277500	-3.67672300	-3.81701300
H	-0.93326100	-2.31162600	-4.22881400
C	-1.21497800	-0.67734400	2.26679200
C	0.98764800	-1.89471800	2.49828400
C	0.79477300	0.66630300	2.10333800
Mn	0.28598300	-0.84237300	1.29886100
N	3.23751900	-0.41316300	0.44001200
N	4.10243400	-0.46071900	-0.55378000
N	3.40084900	-0.95251100	-1.56412900
N	-1.08703100	-2.78261300	-2.19921500
N	-2.12647200	-3.06891800	-1.43189500
N	-1.80197700	-2.57986400	-0.25559500
O	-2.09535500	-0.50595600	3.01195700
O	1.47493600	-2.61880800	3.27326000
O	1.02853900	1.69159500	2.60550600
H	-1.71741800	0.12851200	-2.16332500
C	2.41790400	3.75610600	-0.74990900
C	1.76152700	2.54725400	-0.94398200
C	0.36390800	2.50467400	-1.13254000

C	-0.34125700	3.72438800	-1.14772700
C	0.33207100	4.93018800	-0.95693900
C	1.71104400	4.96237000	-0.74955300
H	3.50144500	3.75433700	-0.60289000
H	2.32661300	1.61230300	-0.95494400
H	-1.42292800	3.72905100	-1.29838300
H	-0.23975100	5.86183100	-0.96982500
H	2.22920200	5.91106900	-0.59645100
N	-0.27139300	1.28806600	-1.27262300
C	-1.60805100	1.16003500	-1.78996400
H	-1.75205700	1.80237800	-2.68288700
C	-2.72250800	1.43845400	-0.81022700
C	-2.47762800	1.78702600	0.52076200
C	-4.05083700	1.34450800	-1.24764200
C	-3.53621800	2.02663000	1.39897100
H	-1.44500200	1.86694700	0.87025400
C	-5.10850300	1.57980900	-0.37283000
H	-4.25442300	1.07614300	-2.28920700
C	-4.85393900	1.92171800	0.95763400
H	-3.32457800	2.29051300	2.43793100
H	-6.13807800	1.49461900	-0.72930000
H	-5.68188500	2.10686900	1.64599800

TS1B

C	0.71549100	1.45100400	0.51941300
C	1.94619300	1.95296100	0.11201100
C	-1.58920100	2.45907700	0.31952600
H	-1.82695700	2.72826800	1.35998300
H	-1.88017100	1.41271300	0.15862200
C	-2.28420700	3.36683200	-0.65852900
H	-3.36918400	3.25997500	-0.53806800
H	-2.02627700	4.42186400	-0.50546000

H	-2.03125000	3.09694700	-1.69264000
C	2.67809500	4.10201900	-1.00416000
H	2.14964500	5.01901000	-1.27347300
H	3.47024700	4.33399700	-0.28419900
H	3.12009200	3.66103800	-1.90543000
C	3.24597500	1.22676100	0.08181100
H	3.75423600	1.27906600	1.06150800
H	3.94032500	1.69195300	-0.63193500
C	2.95860900	-0.18573400	-0.29915000
C	1.83469800	-0.94855400	0.02375300
C	1.14553100	-3.20574200	-0.93186900
H	0.43818900	-3.20196100	-0.09582600
H	1.73913800	-4.12800900	-0.86326900
C	0.42902700	-3.07093800	-2.25466700
H	-0.30437300	-3.88004400	-2.36469700
H	-0.09235400	-2.10009800	-2.29312900
H	1.13559500	-3.13754400	-3.09337900
C	4.97954400	-0.56608100	-1.76927400
H	5.72032100	-0.32335500	-0.99966800
H	5.32566100	-1.42140400	-2.35337900
H	4.84630600	0.29572500	-2.43336900
C	0.10740600	-2.04357000	1.99754300
C	1.61428400	-0.09481200	2.57011900
C	-0.89014600	0.28955600	2.47057600
Mn	0.38431000	-0.37173500	1.36970000
N	-0.13261200	2.46567800	0.17570500
N	0.44974800	3.51350900	-0.38538600
N	1.72393200	3.18623600	-0.41655600
N	3.71818800	-0.91968400	-1.15366700
N	3.17495200	-2.08689500	-1.41985000
N	2.05634400	-2.08450100	-0.70712600
O	-0.07904000	-3.09235200	2.45650600

O	2.45722600	0.10626700	3.34791900
O	-1.67017000	0.69762000	3.22553900
C	-1.72777700	-0.98769000	-0.57392200
H	-1.66343300	-0.76474600	0.56198700
H	-1.55139600	-2.10995500	-0.55383500
C	-3.21786400	-0.81663600	-0.85918100
C	-3.62419400	0.08464700	-1.84570900
C	-4.19780400	-1.51776200	-0.14373500
C	-4.97945100	0.28214700	-2.11969900
H	-2.83366000	0.61497000	-2.38525100
C	-5.55176200	-1.32330300	-0.40924100
H	-3.88726500	-2.22518700	0.63479800
C	-5.94830400	-0.41947400	-1.40125000
H	-5.28446300	0.98864500	-2.89770400
H	-6.30621700	-1.87690800	0.15692300
H	-7.01010600	-0.26510700	-1.61029900
O	-0.89389200	-0.27337000	-1.31222400

Int2B

C	0.12718100	1.18783000	0.37547100
C	1.20427700	1.79861500	-0.26737600
C	-2.12967100	2.19155200	0.97005400
H	-1.99513500	1.99025700	2.04217900
H	-2.65011400	1.32001500	0.54847700
C	-2.90747500	3.46091500	0.75476300
H	-3.90341400	3.34320700	1.19940300
H	-2.42378700	4.32534000	1.22676900
H	-3.03827600	3.68472200	-0.31123600
C	1.59781900	4.08123600	-1.27842300
H	1.01328400	5.00372600	-1.28839300
H	2.54590500	4.25496000	-0.75777700
H	1.79998100	3.76792500	-2.30949600

C	2.56162200	1.26648600	-0.55299600
H	3.25957700	1.55825000	0.25553500
H	2.97353000	1.72342900	-1.46539900
C	2.49930100	-0.20784600	-0.69459300
C	1.62706600	-1.10438300	-0.08596200
C	1.32734300	-3.58445800	-0.56251300
H	0.29844600	-3.36888500	-0.25463900
H	1.81089400	-4.10941700	0.27618700
C	1.34421000	-4.40023300	-1.82722400
H	0.78822300	-5.33141500	-1.66218600
H	0.85473900	-3.83747100	-2.63239100
H	2.36066700	-4.66562000	-2.14175100
C	4.30208200	-0.41758900	-2.45477400
H	5.09054700	0.07170000	-1.87236200
H	4.72204600	-1.26948500	-2.99391000
H	3.88552600	0.29568000	-3.17569100
C	0.19681900	-2.33647100	1.95673200
C	1.10490900	-0.01480600	2.52983400
C	-1.46529500	-0.53615900	2.06904900
Mn	0.11034500	-0.70506100	1.23753700
N	-0.79321300	2.21221300	0.37569400
N	-0.39485600	3.33354400	-0.20177800
N	0.83187600	3.06687600	-0.58738100
N	3.26170000	-0.90657500	-1.57714500
N	2.94444700	-2.18099900	-1.59809000
N	1.97019600	-2.27890700	-0.71195800
O	0.23551500	-3.36216100	2.50819000
O	1.76560700	0.46504400	3.36079100
O	-2.49400800	-0.55482000	2.61608200
C	-1.22999200	-1.55457000	-1.39136600
H	-0.76931700	-1.25602000	-0.22763400
H	-1.64969500	-2.55334800	-1.06684600

C	-2.36277400	-0.54713000	-1.46749800
C	-2.21222400	0.61284000	-2.23278600
C	-3.57481600	-0.77195700	-0.80598900
C	-3.24963700	1.53987600	-2.32483900
H	-1.26290900	0.76221700	-2.75634500
C	-4.61979000	0.14722100	-0.90346600
H	-3.69521300	-1.68113900	-0.20603900
C	-4.45807000	1.30871100	-1.66256200
H	-3.11978500	2.44913800	-2.91903600
H	-5.56461500	-0.04189200	-0.38717300
H	-5.27308000	2.03318700	-1.73769800
O	-0.32708000	-1.54324200	-2.28599000

TS2B

C	-0.66560700	0.91437100	0.72185900
C	-0.19426300	2.17869400	0.36496800
C	-3.06647300	0.17142200	1.07559100
H	-2.85541000	-0.16881900	2.09891500
H	-2.92109400	-0.69898600	0.41945400
C	-4.46280900	0.72070100	0.96809800
H	-5.17507000	-0.07694500	1.21317400
H	-4.63221400	1.55305900	1.66281900
H	-4.68819700	1.07396700	-0.04582400
C	-1.29084200	4.38661800	-0.19045500
H	-2.33081500	4.71919100	-0.21661300
H	-0.73424000	4.98528600	0.53959100
H	-0.84327200	4.51516700	-1.18289900
C	1.20336600	2.65988200	0.22016800
H	1.54244100	3.12989600	1.16389700
H	1.26207200	3.45929200	-0.53416500
C	2.08314800	1.52647500	-0.15261100
C	1.95251300	0.17891500	0.17152400

C	3.30938100	-1.78672400	-0.68984800
H	2.34847900	-2.31129500	-0.62680800
H	3.89546200	-2.09130600	0.19071500
C	4.02244800	-2.11302100	-1.97411300
H	4.17670200	-3.19741200	-2.03348700
H	3.41865900	-1.80156700	-2.83587000
H	5.00379100	-1.62831000	-2.04180200
C	3.66740800	2.84866200	-1.61303600
H	3.91499500	3.59495000	-0.85010700
H	4.56943100	2.58090300	-2.16762500
H	2.92852800	3.26774100	-2.30658600
C	1.53446900	-2.09153900	1.72525300
C	0.76893900	0.11598600	2.80445700
C	-0.87669500	-1.76518200	1.78993700
Mn	0.48007500	-0.73220900	1.26723200
N	-2.02436400	1.13724700	0.73101000
N	-2.40395200	2.36399900	0.40840800
N	-1.27089100	2.99110400	0.18994800
N	3.14631000	1.65153300	-0.99091600
N	3.71034000	0.49342900	-1.24571200
N	2.98729400	-0.36774200	-0.55060200
O	2.17528200	-2.99086000	2.10303100
O	0.94046100	0.69389700	3.80287900
O	-1.68402300	-2.55002200	2.09717400
C	0.06574000	-1.56202100	-1.78318100
H	0.17626800	-1.33843500	-0.29394300
H	0.34980600	-2.63867700	-1.62355500
C	-1.42663400	-1.39443700	-1.85362500
C	-1.97626900	-0.19094600	-2.30811900
C	-2.27926000	-2.45391800	-1.52403000
C	-3.35724600	-0.04496500	-2.41960400
H	-1.29396300	0.62250700	-2.57219300

C	-3.66142800	-2.31542900	-1.64598900
H	-1.84740800	-3.39581200	-1.16867800
C	-4.20356000	-1.10812700	-2.09267900
H	-3.78096100	0.90225900	-2.76512400
H	-4.31939900	-3.15025700	-1.39213400
H	-5.28672500	-0.99410700	-2.18365400
O	0.83376500	-0.80112400	-2.40302100

Int3B

C	-1.41738300	0.15016800	-0.23745100
C	-1.37331100	1.51624700	-0.52424800
C	-3.36752100	-1.18959600	0.69355400
H	-2.72858800	-2.03203200	0.40712500
H	-4.32983100	-1.30901200	0.17695000
C	-3.54589500	-1.11587800	2.19157800
H	-4.02121700	-2.03374600	2.55883300
H	-2.57926800	-1.00999300	2.70141400
H	-4.18215500	-0.26775500	2.47676200
C	-3.06355900	3.39213600	-0.46800300
H	-4.12343000	3.43551000	-0.20794500
H	-2.50085800	4.06531400	0.18893300
H	-2.92894400	3.70499200	-1.50951900
C	-0.19445200	2.30618600	-0.96610000
H	-0.32104700	3.37515200	-0.74174000
H	-0.08506100	2.23994400	-2.06632600
C	1.01595300	1.75350100	-0.30317900
C	1.28390800	0.41557300	-0.00062100
C	3.32119300	-0.54406000	1.18111700
H	2.67599800	-1.43060600	1.20081300
H	3.54483200	-0.27740300	2.22381300
C	4.58605400	-0.77875500	0.39114200
H	5.15805100	-1.60275200	0.83521200

H	4.36276000	-1.04606900	-0.64951600
H	5.22508700	0.11402600	0.39016400
C	2.24456800	3.93034000	0.05384500
H	1.46706100	4.44598700	0.62953700
H	3.22477300	4.17578500	0.46834800
H	2.19771400	4.25530900	-0.99136400
C	1.53312700	-2.12751700	-1.01696100
C	-0.00627100	-1.88498800	1.14853600
C	-0.98461400	-2.31580500	-1.32850200
Mn	0.11353000	-1.19226500	-0.49653800
N	-2.72363000	0.00661700	0.15947500
N	-3.45695500	1.11666100	0.12959800
N	-2.60898900	2.02950000	-0.29355600
N	2.06206000	2.49733600	0.13815000
N	2.99078900	1.76217600	0.71104200
N	2.50270800	0.52728500	0.62163100
O	2.45180400	-2.74292800	-1.39552200
O	-0.08737300	-2.37176200	2.21007600
O	-1.69318300	-3.02716100	-1.92569100
H	0.18323600	-0.48353400	-1.94154800

TS3B

C	-0.98874200	-0.45534900	-0.58683300
C	-1.59367300	0.78953300	-0.39984900
C	-1.95574600	-2.47720800	-1.80478300
H	-0.90960600	-2.75788200	-1.96016700
H	-2.42974800	-2.46830900	-2.79505700
C	-2.67074400	-3.40213900	-0.84892500
H	-2.59769000	-4.43846200	-1.20257200
H	-2.22987900	-3.35988100	0.15581800
H	-3.73516200	-3.14377300	-0.76625100
C	-3.78213100	1.82399800	-1.11747700

H	-4.61765900	1.45949200	-1.71983000
H	-4.14304000	2.10057300	-0.11995500
H	-3.34058400	2.70159700	-1.60583700
C	-1.08403700	1.97944200	0.32252700
H	-1.42392200	1.96508500	1.37702500
H	-1.51331700	2.90013500	-0.10324200
C	0.39700500	2.02227200	0.25651200
C	1.31400900	0.97844300	0.10228100
C	3.85479900	1.16819800	-0.07493400
H	4.00843100	0.38548700	0.67699500
H	4.52284200	1.99831400	0.18455100
C	4.11089600	0.66180700	-1.47199700
H	5.11853000	0.23354200	-1.54389700
H	3.38942700	-0.12367500	-1.73617000
H	4.02890700	1.47078500	-2.21126100
C	0.57237500	4.53072000	0.45050300
H	-0.03584600	4.60539100	1.35862600
H	1.41681000	5.22024500	0.51770300
H	-0.04324400	4.79248600	-0.41943400
C	2.55084700	-1.59397500	0.32552800
C	0.42001500	-0.94261000	1.79291900
C	0.53227200	-2.80549000	-0.04379000
Mn	0.87125600	-1.06012000	0.07454600
N	-1.94921800	-1.09935300	-1.32989100
N	-3.03607600	-0.38910100	-1.60019300
N	-2.80062300	0.76675600	-1.01965700
N	1.09074300	3.18784300	0.30925100
N	2.38644900	3.00110000	0.19876100
N	2.49731100	1.68011500	0.08706900
O	3.61443500	-2.06062700	0.45729400
O	0.11616800	-0.85968800	2.91613400
O	0.42661300	-3.96732400	-0.08155400

H	1.16608300	-1.09961600	-1.61913800
C	-1.92661500	2.20391900	-4.11782600
C	-1.53384500	0.87191500	-4.16619100
C	-0.28617700	0.45069800	-3.64869400
C	0.54942000	1.44404500	-3.08237700
C	0.15118300	2.77656300	-3.04061700
C	-1.08937400	3.17050500	-3.54870900
H	-2.90036100	2.49528400	-4.52188800
H	-2.19056800	0.10994600	-4.59481800
H	1.51314400	1.16640200	-2.65266000
H	0.82472600	3.52138100	-2.60431700
H	-1.39735000	4.21853400	-3.51038500
N	0.00006100	-0.89630000	-3.68246400
C	1.15952300	-1.31457800	-3.22183100
H	2.04891400	-0.65502600	-3.22599700
C	1.50429600	-2.75304200	-3.29808100
C	0.61911800	-3.69129300	-3.84595700
C	2.74141200	-3.19780200	-2.80964300
C	0.95544400	-5.04297400	-3.88498500
H	-0.33286100	-3.33443200	-4.24497800
C	3.07906600	-4.54758700	-2.84683800
H	3.43717100	-2.47088400	-2.37905100
C	2.18270000	-5.47691700	-3.37999600
H	0.25655100	-5.76464500	-4.31533400
H	4.04368000	-4.87866100	-2.45471500
H	2.44372100	-6.53752500	-3.40727700

Int4B

C	1.44367300	0.22141700	1.01210500
C	0.96104300	-0.92340200	1.65388800
C	3.89080700	0.95270200	0.77890300
H	3.81857200	1.06355100	-0.30979500

H	4.81187100	0.39040200	0.97580100
C	3.89385800	2.28086500	1.49263300
H	4.73679600	2.88850100	1.14113000
H	2.97263300	2.84663100	1.30663300
H	3.99991200	2.14880900	2.57774800
C	2.03906200	-2.87049100	2.83966200
H	3.07546000	-3.14221100	3.05283600
H	1.48204100	-2.78652500	3.77996200
H	1.57704100	-3.64450600	2.21490900
C	-0.41441400	-1.42854900	1.87656900
H	-0.53728900	-1.70891300	2.93864400
H	-0.57897200	-2.35200600	1.28874900
C	-1.42101100	-0.40723300	1.51604500
C	-1.28782400	0.81223900	0.85308500
C	-3.07365100	2.62138000	0.53627300
H	-2.19524700	3.22448400	0.28279600
H	-3.53140800	3.08229800	1.42266400
C	-4.05781500	2.53730200	-0.60330200
H	-4.41061300	3.54369600	-0.86070400
H	-3.60077200	2.09882000	-1.49860200
H	-4.93086300	1.93043900	-0.32939300
C	-3.34049300	-1.62426400	2.60231400
H	-3.02595800	-1.61909100	3.65329200
H	-4.42529300	-1.50726900	2.54440200
H	-3.05046700	-2.57165700	2.13404600
C	-0.65468800	2.30432900	-1.40970300
C	0.39138600	2.96374500	0.92788600
C	1.75460900	2.13297600	-1.09424500
Mn	0.35379300	1.55868500	-0.13591900
N	2.80166600	0.07645200	1.20657900
N	3.16817400	-1.00301600	1.87701500
N	2.03399000	-1.60627200	2.13916500

N	-2.72291200	-0.52873600	1.88835600
N	-3.43613300	0.52153900	1.55787500
N	-2.56551400	1.31495700	0.95576500
O	-1.24072400	2.77429100	-2.29957900
O	0.38419900	3.89248700	1.63174900
O	2.58535100	2.62160300	-1.74948700
H	0.15053200	-0.01418600	-1.18350100
C	-4.11507600	-2.88091000	-0.75391600
C	-2.73017000	-2.92006100	-0.69572000
C	-1.91925000	-1.89330800	-1.26475200
C	-2.61633000	-0.81203600	-1.88307600
C	-4.00795800	-0.78976100	-1.93696300
C	-4.77645700	-1.81479200	-1.37922900
H	-4.69511900	-3.69436400	-0.30657900
H	-2.21645400	-3.75984700	-0.21570600
H	-2.05469900	0.01679900	-2.32284300
H	-4.50833000	0.05065000	-2.42815600
H	-5.86716200	-1.78543200	-1.42989600
N	-0.57532500	-2.01160300	-1.15117600
C	0.22122900	-1.07035600	-1.75420800
H	-0.09544500	-0.71367900	-2.76298700
C	1.68974000	-1.39945100	-1.76771000
C	2.20485100	-2.45078700	-1.00307300
C	2.57428500	-0.63251900	-2.53594800
C	3.57482900	-2.71250300	-0.98332300
H	1.50020500	-3.05549900	-0.42686700
C	3.94346400	-0.89425800	-2.52489100
H	2.17939600	0.18357200	-3.14931600
C	4.45069700	-1.93216200	-1.73970600
H	3.96307100	-3.53146800	-0.37077500
H	4.61898000	-0.28369500	-3.12982700
H	5.52414100	-2.13641200	-1.72478500

TS4B

C	-0.19176700	-1.08315700	1.26528300
C	-1.54119600	-1.41986700	1.15655300
C	1.74207800	-2.63976000	1.91168700
H	2.36841600	-2.14326900	1.16096500
H	1.83335900	-3.71762900	1.73040500
C	2.12201400	-2.29132500	3.33121200
H	3.18841500	-2.48688500	3.49720500
H	1.93576000	-1.23302100	3.55698000
H	1.54757100	-2.89329300	4.04783100
C	-2.86477900	-3.54288500	1.41860600
H	-3.30697500	-3.50089500	0.41458800
H	-2.59975300	-4.57378400	1.66381500
H	-3.58625800	-3.16334600	2.15132100
C	-2.72438400	-0.62872600	0.76945000
H	-3.45006600	-0.58208900	1.60386100
H	-3.25967100	-1.14355300	-0.05389000
C	-2.37447300	0.73362300	0.32853600
C	-1.16328900	1.42461000	0.30812100
C	-0.77305600	3.82085900	-0.55266900
H	-0.08890000	3.99387900	0.28397100
H	-1.48363300	4.65600900	-0.55443800
C	-0.06062500	3.71113400	-1.87828300
H	0.54277200	4.60988200	-2.05530900
H	0.61144300	2.84269000	-1.91151400
H	-0.77885200	3.61239800	-2.70317900
C	-4.72980700	1.25154200	-0.39137300
H	-5.22377900	1.10093700	0.57569400
H	-5.18960100	2.09492100	-0.91140800
H	-4.82459500	0.34192500	-0.99913200
C	1.57025000	2.28587400	0.55627700

C	0.38880400	1.24370200	2.57862500
C	2.37494600	0.22307600	1.35837700
Mn	0.69859000	0.75057300	0.93546500
N	0.35402700	-2.29961700	1.61640900
N	-0.50767600	-3.29709300	1.71903100
N	-1.66249400	-2.74287300	1.44363200
N	-3.32818400	1.54713400	-0.19285600
N	-2.84210500	2.71052000	-0.55253400
N	-1.56168600	2.63036700	-0.23664700
O	2.25503200	3.21264200	0.41315500
O	0.14322700	1.53816000	3.67486500
O	3.48180100	0.07019200	1.66770500
H	0.78471100	0.78290800	-1.55605300
C	-2.90412700	-2.68072500	-2.13365700
C	-1.56231500	-2.56574900	-1.81339400
C	-0.81128700	-1.35921000	-2.04294700
C	-1.56964100	-0.28197500	-2.62630100
C	-2.92222400	-0.41203200	-2.92439400
C	-3.62239400	-1.60043400	-2.68171900
H	-3.41522600	-3.63461700	-1.95844800
H	-1.02145400	-3.41488200	-1.37979400
H	-1.07992800	0.67634000	-2.82357600
H	-3.45157000	0.44653400	-3.35632300
H	-4.68032900	-1.69615700	-2.93758500
N	0.48291000	-1.32676000	-1.71893500
C	1.18951100	-0.14048200	-2.05621700
H	1.08904800	0.14002000	-3.13861800
C	2.66692300	-0.20649900	-1.75494100
C	3.29043500	-1.41379600	-1.42122300
C	3.45185000	0.95348900	-1.81831800
C	4.65849000	-1.45867800	-1.14807500
H	2.66618900	-2.30895700	-1.37520100

C	4.81745300	0.91355600	-1.54506800
H	2.97857900	1.90611900	-2.08106000
C	5.42776000	-0.29661100	-1.20607200
H	5.12835800	-2.41031600	-0.88336500
H	5.40909700	1.83156600	-1.59307700
H	6.49785400	-0.33068200	-0.98683900

PhCH2OH

C	-1.90250000	-0.62030000	0.00000000
H	-2.11790000	-1.25800000	-0.88120000
H	-2.11790000	-1.25790000	0.88120000
C	-0.44200000	-0.27440000	0.00000000
C	0.51470000	-1.29900000	0.00000000
C	-0.00620000	1.05370000	0.00000000
C	1.87520000	-1.00250000	0.00000000
H	0.18480000	-2.34260000	0.00000000
C	1.35820000	1.35160000	0.00000000
H	-0.74800000	1.85390000	0.00000000
C	2.30340000	0.32730000	0.00000000
H	2.60750000	-1.81380000	0.00000000
H	1.68360000	2.39500000	0.00000000
H	3.37060000	0.56120000	0.00000000
O	-2.68240000	0.54540000	0.00000000
H	-3.60820000	0.28090000	0.00000000

PhCHO

C	-0.53680000	0.21140000	0.00000000
C	0.35690000	1.29100000	0.00000000
C	-0.04350000	-1.10300000	0.00000000
C	1.73050000	1.06250000	0.00000000
H	-0.03660000	2.31200000	0.00000000
C	1.32700000	-1.33000000	0.00000000

H	-0.75780000	-1.93000000	0.00000000
C	2.21340000	-0.24730000	0.00000000
H	2.42760000	1.90290000	0.00000000
H	1.71440000	-2.35110000	0.00000000
H	3.29080000	-0.42870000	0.00000000
C	-1.98680000	0.46510000	0.00000000
H	-2.26240000	1.55270000	0.00000000
O	-2.84260000	-0.39450000	0.00000000

PhCH2NHPh

C	3.80270000	-0.24150000	0.59140000
C	2.83730000	0.75110000	0.70480000
C	1.60780000	0.64470000	0.01970000
C	1.38970000	-0.49510000	-0.78150000
C	2.36910000	-1.48160000	-0.88410000
C	3.58130000	-1.37070000	-0.20310000
H	4.74570000	-0.13000000	1.13310000
H	3.02220000	1.63000000	1.32900000
H	0.44790000	-0.61280000	-1.32170000
H	2.17380000	-2.35520000	-1.51180000
H	4.34180000	-2.14930000	-0.28840000
N	0.65800000	1.62950000	0.15480000
H	0.94540000	2.47860000	0.62060000
C	-0.55450000	1.66960000	-0.61400000
H	-0.96760000	2.68610000	-0.52330000
H	-0.34830000	1.54120000	-1.69470000
C	-1.61060000	0.67040000	-0.19580000
C	-1.62180000	0.11340000	1.08690000
C	-2.61870000	0.30400000	-1.09630000
C	-2.62080000	-0.78430000	1.46390000
H	-0.83040000	0.38650000	1.79000000
C	-3.62070000	-0.58940000	-0.72090000

H	-2.61440000	0.72340000	-2.10730000
C	-3.62500000	-1.13790000	0.56260000
H	-2.61260000	-1.21300000	2.46920000
H	-4.39900000	-0.86460000	-1.43700000
H	-4.40590000	-1.84310000	0.85670000

PhCHNPh

C	-4.07360000	-1.01000000	-0.41120000
C	-2.70210000	-1.23770000	-0.36090000
C	-1.82280000	-0.22140000	0.05380000
C	-2.36140000	1.01520000	0.45780000
C	-3.73680000	1.22910000	0.42280000
C	-4.59870000	0.22360000	-0.01910000
H	-4.74120000	-1.80600000	-0.74970000
H	-2.28050000	-2.20170000	-0.65440000
H	-1.69490000	1.79700000	0.83090000
H	-4.14100000	2.18950000	0.75200000
H	-5.67690000	0.39570000	-0.04390000
N	-0.45920000	-0.50680000	0.08880000
C	0.40270000	0.40400000	-0.17450000
H	0.09680000	1.41930000	-0.50290000
C	1.84240000	0.18170000	-0.08510000
C	2.37020000	-1.05850000	0.31680000
C	2.72440000	1.22650000	-0.40520000
C	3.74460000	-1.24330000	0.39450000
H	1.67630000	-1.86560000	0.56220000
C	4.10230000	1.03960000	-0.32510000
H	2.31740000	2.19220000	-0.71830000
C	4.61460000	-0.19510000	0.07450000
H	4.14790000	-2.20940000	0.70670000
H	4.77960000	1.85890000	-0.57560000
H	5.69500000	-0.34390000	0.13750000

9 References

(1) S. Friães, C. S. B. Gomes, B. Royo, *Organometallics* **2023**, *42*, 1803–1809.

(2) L. J. Farrugia, *J. Appl. Crystallogr.* **2012**, *45*, 849–854.

(3) C. B. Hübschle, G. M. Sheldrick, B. Dittrich, *J. Appl. Crystallogr.* **2011**, *44*, 1281–1284.

(4) G. M. Sheldrick, *Acta Crystallogr. Sect. C Struct. Chem.* **2015**, *71*, 3–8.

(5) A. L. Spek, *Acta Cryst*. **2015**, *C71*, 9-18.

(6) C. F. Macrae, I. Sovago, S. J. Cottrell, P. T. A. Galek, P. McCabe, E. Pidcock, M. Platings, G. P. Shields, J. S. Stevens, M. Towler, P. A. Wood, *J. Appl. Cryst*. **2020**, *53*, 226-235.

(7) Gaussian 16, M. J. Frisch, G. W. Trucks, H. B. Schlegel, G. E. Scuseria, M. A. Robb, J. R. Cheeseman, G. Scalmani, V. Barone, G. A. Petersson, H. Nakatsuji, X. Li, M. Caricato, A. V. Marenich, J. Bloino, B. G. Janesko, R. Gomperts, B. Mennucci, H. P. Hratchian, J. V. Ortiz, A. F. Izmaylov, J. L. Sonnenberg, D. Williams-Young, F. Ding, F. Lipparini, F. Egidi, J. Goings, B. Peng, A. Petrone, T. Henderson, D. Ranasinghe, V. G. Zakrzewski, J. Gao, N. Rega, G. Zheng, W. Liang, M. Hada, M. Ehara, K. Toyota, R. Fukuda, J. Hasegawa, M. Ishida, T. Nakajima, Y. Honda, O. Kitao, H. Nakai, T. Vreven, K. Throssell, J. A. Montgomery, Jr., J. E. Peralta, F. Ogliaro, M. J. Bearpark, J. J. Heyd, E. N. Brothers, K. N. Kudin, V. N. Staroverov, T. A. Keith, R. Kobayashi, J. Normand, K. Raghavachari, A. P. Rendell, J. C. Burant, S. S. Iyengar, J. Tomasi, M. Cossi, J. M. Millam, M. Klene, C. Adamo, R. Cammi, J. W. Ochterski, R. L. Martin, K. Morokuma, O. Farkas, J. B. Foresman, and D. J. Fox, Gaussian, Inc., Wallingford CT, 2016.

(8) J.-D. C. M. Head-Gordon, *J. Chem. Phys*. **2008**, *128*, 084106.

(9) H. B. Hratchian, H. P.; Schlegel, Theory and Applications of Computational Chemistry, Elsevier: Amsterdam, 2005.

(10) P. J. Stephen, F. J. Devlin, C. F. Chabalowski, M. J. Frisch, *J. Phys. Chem*. **1994**, *98*, 11623–11627.
